# Cembranoids from a Chinese Collection of the Soft Coral *Lobophytum crassum*

**DOI:** 10.3390/md14060111

**Published:** 2016-06-03

**Authors:** Min Zhao, Shimiao Cheng, Weiping Yuan, Yiyuan Xi, Xiubao Li, Jianyong Dong, Kexin Huang, Kirk R. Gustafson, Pengcheng Yan

**Affiliations:** 1 School of Pharmaceutical Sciences, Wenzhou Medical University, Wenzhou 325035, China; miniezhao09@163.com (M.Z.); yuan0525@126.com (W.Y.); xiyiyuan2@163.com (Y.X.); jianyd@wzmc.edu.cn (J.D.); hkx@wzmc.edu.cn (K.H.); 2 The Fifth Affiliated Hospital, Wenzhou Medical University, Lishui 323000, China; lschengshimiao@163.com; 3 Key Laboratory of Marine Bio-Resources Sustainable Utilization, South China Sea Institute of Oceanology, Chinese Academy of Sciences, Guangzhou 510301, China; lixiubao@scsio.ac.cn; 4 Molecular Targets Laboratory, Center for Cancer Research, National Cancer Institute, Frederick, MD 21702-1201, USA

**Keywords:** soft coral, *Lobophytum crassum*, cembrane-based diterpenes, NO inhibition

## Abstract

Ten new cembrane-based diterpenes, locrassumins A–G (**1**–**7**), (–)-laevigatol B (**8**), (–)-isosarcophine (**9**), and (–)-7*R*,8*S*-dihydroxydeepoxysarcophytoxide (**10**), were isolated from a South China Sea collection of the soft coral *Lobophytum crassum*, together with eight known analogues (**11**–**18**). The structures of the new compounds were determined by extensive spectroscopic analysis and by comparison with previously reported data. Locrassumin C (**3**) possesses an unprecedented tetradecahydrobenzo[3,4]cyclobuta[1,2][8]annulene ring system. Compounds **1**, **7**, **12**, **13**, and **17** exhibited moderate inhibition against lipopolysaccharide (LPS)-induced nitric oxide (NO) production with IC_50_ values of 8–24 μM.

## 1. Introduction

**Figure 1:**
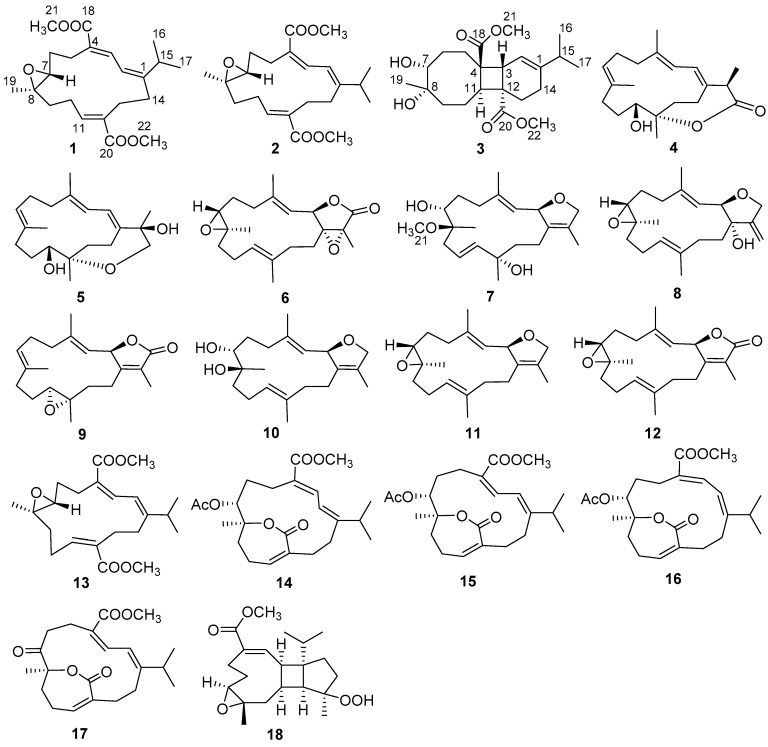
Structures of compounds **1**–**18**.

Soft corals of the genus *Lobophytum* (family Alcyoniidae) have proven to be a rich source of structurally diverse diterpenes, especially macrocyclic cembranoids characterized by their 14-membered carbocyclic skeleton. To date, numerous marine cembranoids and novel derivatives (mainly formed by dimerization, cyclic addition, or ring rearrangement) have been isolated from *Lobophytum* species and other genera including *Sinularia* and *Sarcophyton* [1]. Some of these metabolites merit further study because of their significant ecological and pharmacological bioactivities, such as antifouling, antifeeding, cytotoxic, antibacterial, antiviral, and anti-inflammatory properties [2,3,4,5,6,7,8,9,10,11,12,13,14,15,16,17,18,19,20,21,22,23,24]. Species *L. crassum* is widely distributed in the tropical waters of the world and is well known to produce a variety of oxygenated cembranoids, the structural variety of which is often correlated with geographic variation and environmental conditions [3,4,5,6,7,8,9,10,11,12,13,14,15,16,17,18,19,20,21,22,23,24]. However, the soft coral *L. crassum* in the South China Sea has been rarely examined chemically [22,23,24]. In the course of our investigation of bioactive substances produced by marine invertebrates from the South China Sea [25,26,27], a specimen of *L. crassum* was collected. Chemical examination of this specimen led to the isolation of 18 cembrane-based diterpenes, including nine new cembranoids (**1**, **2**, and **4**–**10**), an unprecedented diterpene possessing a tetradecahydrobenzo[3,4]cyclobuta[1,2][8]annulene ring system (**3**), and eight known analogues (**11**–**18**) (Figure 1). All compounds were tested for their inhibitory effects on lipopolysaccharide (LPS)-induced nitric oxide (NO) production in mouse peritoneal macrophages (PEMФ). This paper reports details of the isolation, structure elucidation, and biological evaluation of these compounds.

## 2. Results and Discussion

**Figure 2:**
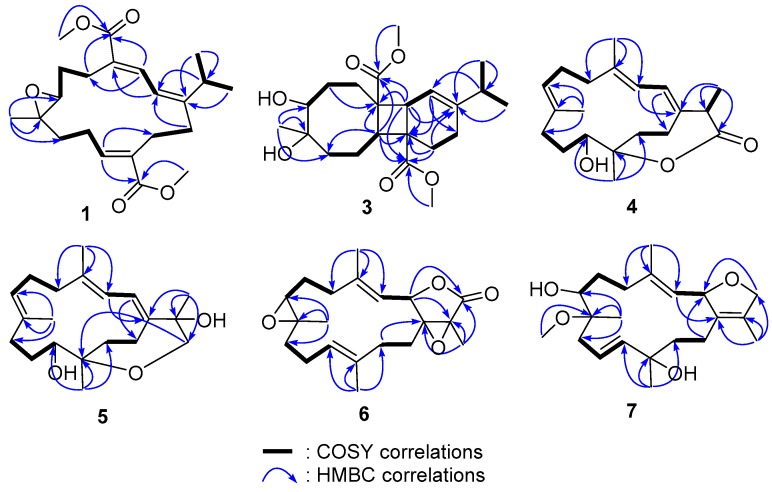
Key COSY and HMBC correlations for **1** and **3**–**7**.

**Figure 3:**
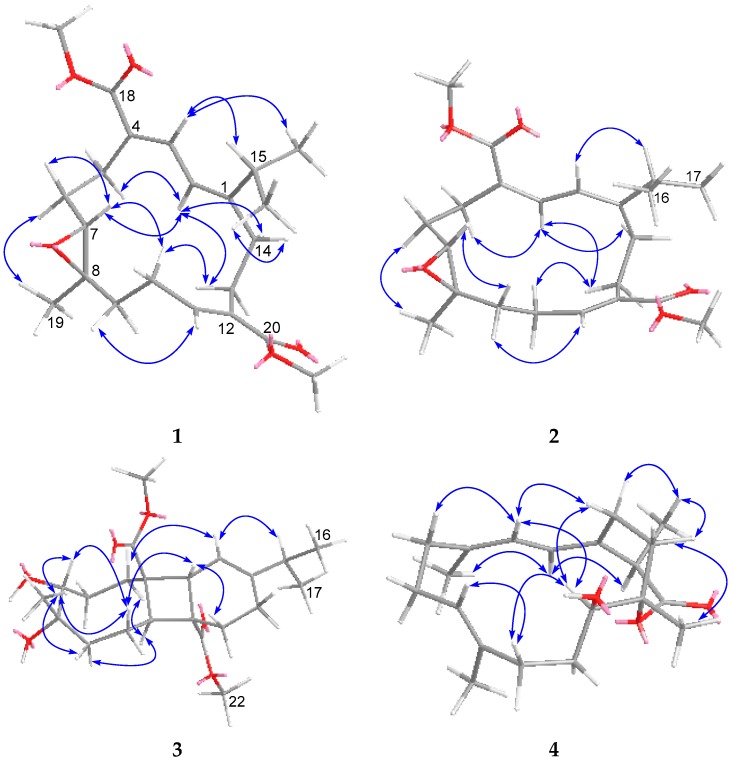

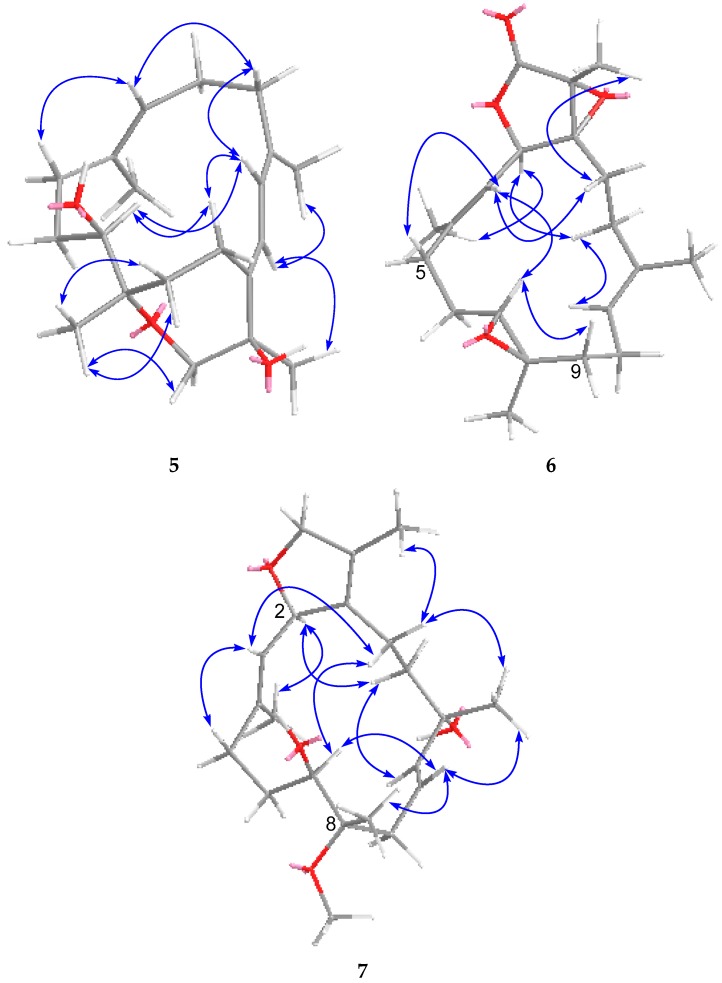
Key NOE correlations and computer-generated models using MM2 force field calculations for **1**–**7**.

Locrassumin A (**1**) was assigned a molecular formula of C_22_H_32_O_5_ according to its HRESIMS (*m*/*z* 399.2134 [M + Na]^+^, calcd for C_22_H_32_O_5_Na, 399.2147) and NMR data (Figures S1–S7). The ^1^H NMR spectrum showed signals for three olefinic protons (δ_H_ 7.70 d, *J* = 12.0 Hz, H-3; 6.80 t, *J* = 7.2 Hz, H-11; 6.24 d, *J* = 12.0 Hz, H-2), two methoxy groups (δ_H_ 3.77 s, H_3_-21; 3.75 s, H_3_-22), and three additional methyls (δ_H_ 1.18 s, H_3_-19; 1.00 d, *J* = 6.6 Hz, H_3_-16; 0.95 d, *J* = 6.6 Hz, H_3_-17), while the ^13^C NMR spectrum exhibited 22 carbon signals including two ester carbonyls, six olefinic carbons, and two carbons indicative of an epoxide (Table 1 and Table 2). These NMR data were very similar to those of the known cembranoids sarcrassin A [28] and sarcophytonolides B [29] and O (**13**) [30]. Detailed analysis of COSY and HMBC correlations (Figure 2) confirmed that **1** shared the same planar structure with those three analogues. The NOE correlations of H-3/H-15 (δ_H_ 3.20 m), H-2/H-5b (δ_H_ 2.55 m), H-2/H-14a (δ_H_ 2.42 m), and H_3_-19/H-6b (δ_H_ 1.57 m) (Figure 3) revealed that the geometries of the C-1/C-2 and C-3/C-4 double bonds and configurations of the 7,8-epoxy ring in **1** were identical to those in sarcrassin A [28]. In addition, the NOE correlation of H-10b (δ_H_ 2.15 m)/H-13a (δ_H_ 2.63 m) and the lack of an NOE correlation between H-11 and H_2_-13 revealed *E* geometry for the C-11/C-12 double bond. Thus, **1** was established as the 11*E* isomer of sarcrassin A [28].

Locrassumin B (**2**) was also isomeric with sarcrassin A [28] and sarcophytonolides B [29] and O (**13**) [30] based on the compatible HRESIMS (*m/z* 399.2135 [M + Na]^+^, calcd for C_22_H_32_O_5_Na, 399.2147) and 1D and 2D NMR data (Figures S8–S14). The NOE correlations of H-2 (δ_H_ 6.23 d, *J* = 12.0 Hz)/H-15 (δ_H_ 2.17 m), H-3 (δ_H_ 7.62 d, *J* = 12.0 Hz)/H-5a (δ_H_ 2.66 m), H-3/H-14a (δ_H_ 2.83 m), and H_3_-19 (δ_H_ 1.12 s)/H-6b (δ_H_ 1.71 m) were indicative of 1*E*, 3*Z*, 7R*, and 8R* configurations (Figure 3), consistent with those of sarcophytonolide O (**13**) [30], while the NOE correlation of H-10a (δ_H_ 2.12 m)/H-13a (δ_H_ 2.69 m) and the lack of NOE correlation between H-11 and H_2_-13 allowed for the assignment of 11*E*, instead of 11*Z* as in **13**. Thus, **2** was elucidated as the 11*E* isomer of sarcophytonolide O (**13**) [30].

Locrassumin C (**3**) had a molecular formula of C_22_H_34_O_6_ as determined by HRESIMS (*m*/*z* 417.2251 [M + Na]^+^, calcd for C_22_H_34_O_6_Na, 417.2253) and NMR data, requiring six degrees of unsaturation (Figures S15–S21). The IR absorptions at 3450 and 1726 cm^−1^ indicated the presence of hydroxy and carbonyl functionalities. The ^13^C NMR spectrum showed 22 carbon signals including two ester carbonyls (δ_C_ 177.4 and 176.7) and two olefinic carbons (δ_C_ 148.3, C; 113.8, CH) (Table 2), which accounted for three of the six degrees of unsaturation. Thus, **3** had to be tricyclic. COSY correlations established the subunits from C-2 to C-3, C-5 to C-7, C-9 to C-11, and C-13 to C-14, while their connectivities were completed by detailed analysis of HMBC correlations (Figure 2). The HMBC correlations from the olefinic proton H-2 (δ_H_ 5.31 d, *J* = 3.0 Hz), the aliphatic methine proton H-3 (δ_H_ 3.17 d, *J* = 3.0 Hz), and H_2_-13 (δ_H_ 2.08 m; 1.78 m) to the non-protonated carbon C-12 (δ_C_ 45.2), from H-3 and H_2_-13 to the non-protonated olefinic carbon C-1 (δ_C_ 148.3), and from H-2 to C-14 (δ_C_ 23.2, CH_2_) led to the establishment of a cyclohexene ring. The HMBC correlations from H_3_-19 (δ_H_ 1.14 s) to C-9 (δ_C_ 39.3, CH_2_) and two oxygenated carbons C-7 (δ_C_ 73.7, CH) and C-8 (δ_C_ 74.9, C) and from the aliphatic methine proton H-11 (δ_H_ 3.24 dd, *J* = 7.2, 5.4 Hz) and H_2_-6 (δ_H_ 1.74 m; 1.57 m) to another non-protonated carbon C-4 (δ_C_ 50.0) constructed a cyclooctane ring and revealed that C-7 and C-8 were hydroxylated and C-8 was also substituted by a methyl group. In addition, the HMBC correlations from H-3 to C-4 and from H-11 to C-12 finally connected the cyclohexene and cyclooctane rings to form an 8,4,6-tricarbocyclic nucleus. Further HMBC correlations from H-3 and H-11 to the two ester carbonyl carbons C-18 (δ_C_ 177.4) and C-20 (δ_C_ 176.7), from the methoxy protons H_3_-21 (δ_H_ 3.76 s) and H_3_-22 (δ_H_ 3.68 s) to C-18 and C-20, respectively, and from H_2_-13 to C-20 disclosed that C-4 and C-12 were substituted by methyl esters. Direct linkage of an isopropyl group to C-1 was inferred by the HMBC correlations from H_3_-16 (δ_H_ 1.00 d, *J* = 6.6 Hz) and H_3_-17 (δ_H_ 1.01 d, *J* = 6.6 Hz) to the non-protonated olefinic carbon C-1. Thus, the planar structure of **3** was established as depicted in Figure 1.

**Figure 4:**
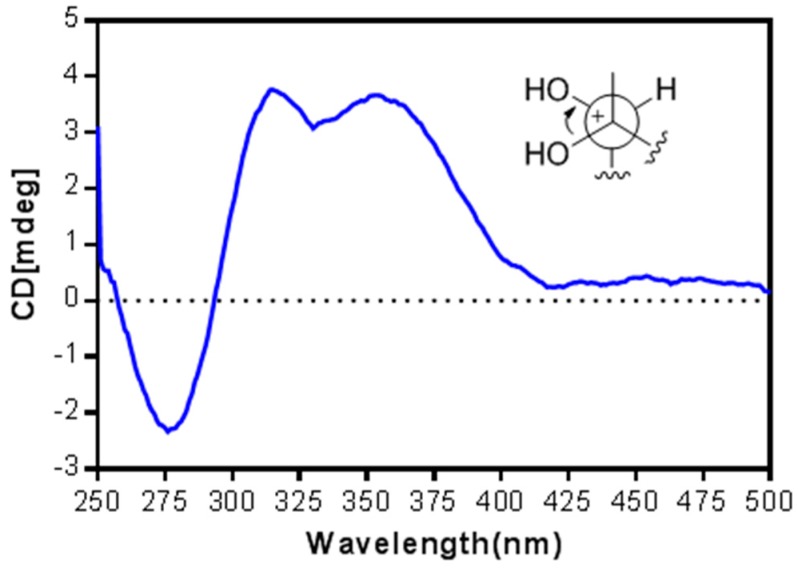
ICD curve of **3** induced by Mo_2_(OAc)_4_ in DMSO.

The relative configuration of **3** was determined on the basis of coupling constant and NOESY analysis (Figure 3). The NOE correlations of H-2/H-5a (δ_H_ 1.92 m), H-3/H-13b (δ_H_ 1.78 m), H-3/H-10a (δ_H_ 1.87 m), and H-11/H-5b (δ_H_ 1.75 m) suggested the *trans* fusions of the ring system and the opposite orientation of H-3 and H-11. In addition, the coupling pattern of H-7 (δ_H_ 3.58 d, *J* = 10.8 Hz) indicated its axial orientation (one large coupling due to dihedral angles of approximately 180° and 90° with the H_2_-6 protons), while the NOE correlations of H-7/H_3_-19, H-7/H-10a, and H_3_-19/H-10a suggested the same orientation of H-7, H_3_-19, and H-10a. Furthermore, the absolute configuration of the 7,8-diol was determined by an *in situ* dimolybdenum CD method [31,32], based on which the sign of the induced CD (ICD) bands at 310, 350, and 400 nm reflected the O-C-C-O torsion angle. After addition of dimolybdenum tetraacteate [Mo_2_(OAc)_4_] into a DMSO solution of **3**, a metal complex was generated immediately and the ICD spectrum was acquired. The positive CD effects observed at 318 and 357 nm (Figure 4) allowed for the assignment of the 7*R* and 8*S* configurations. Accordingly, the configurations of the remaining chiral centers of **3** were assigned as 3*R*, 4*R*, 11*R*, and 12*R*.

It is interesting to note that **3** represents an unprecedented diterpenoid with a tetradecahydrobenzo[3,4]cyclobuta[1,2][8]annulene ring system, which could be derived from the coisolated cembranoids locrassumins A (**1**), B (**2**), or sarcophytonolide O (**13**) [30] via a series of isomerization, intramolecuar [2 + 2] cycloaddition between C-3/C-4 and C-11/C-12 double bonds [2], and hydrolysis reactions.

The molecular formula of locrassumin D (**4**) was determined to be C_20_H_30_O_3_ on the basis of HRESIMS (*m/z* 341.2094 [M + Na]^+^, calcd for C_20_H_30_O_3_Na, 341.2093) and NMR data, implying six degrees of unsaturation (Figures S22–S28). The IR absorption at 1707 cm^−1^ indicated the presence of a carbonyl functionality. A carbonyl carbon (δ_C_ 177.8) and six olefinic carbons were evident by ^13^C NMR data (Table 2), requiring a bicyclic structure for the remaining two degrees of unsaturation. A conjugated diene was easily recognized by a COSY correlation between the two olefinic protons H-2 (δ_H_ 6.02 dd, *J* = 11.4, 1.8 Hz) and H-3 (δ_H_ 5.87 d, *J* = 11.4 Hz) as well as the HMBC correlations from H_3_-18 (δ_H_ 1.79 s) to two olefinic carbons C-3 (δ_C_ 121.7, CH) and C-4 (δ_C_ 138.2, C) and from H-3 to C-1 (δ_C_ 133.7, C). Further COSY correlations established the other four subunits from C-5 to C-7, C-9 to C-11, C-13 to C-14, and C-15 to C-16, while HMBC correlations from H_3_-18 to a methylene carbon C-5 (δ_C_ 40.4, CH_2_), from H_3_-19 (δ_H_ 1.30 s) to two olefinic carbons C-7 (δ_C_ 125.0, CH) and C-8 (δ_C_ 136.3, C) and a methylene carbon C-9 (δ_C_ 35.5, CH_2_), from H_3_-20 (δ_H_ 1.28 s) to two oxygenated carbons C-11 (δ_C_ 67.9, CH) and C-12 (δ_C_ 87.0, C) and a methylene carbon C-13 (δ_C_ 32.9, CH_2_), from H_3_-16 (δ_H_ 1.47 d, *J* = 7.2 Hz) to the ester carbonyl C-17 (δ_C_ 177.8) and C-1, and from H-2 to a methylene carbon C-14 (δ_C_ 22.1, CH_2_) (Figure 2) finally connected the subunits to form a cembrane skeleton, in which C-1/C-2, C-3/C-4, and C-7/C-8 formed double bonds and C-11 was hydroxylated. In addition, an ester linkage between C-12 and C-17 could be inferred by the chemical shifts of C-12 and C-17 and the remaining one degree of unsaturation in the molecule. The geometries of 3*E* and 7*E* were indicated by the diagnostic chemical shifts of C-18 and C-19 (<20 ppm) [33,34] and confirmed by the NOE correlations of H-3/H-5b (δ_H_ 1.98 m) and H-7 (δ_H_ 5.11 dd, *J* = 10.2, 5.4 Hz)/H-9a (δ_H_ 2.25 m), respectively. The coupling constant value of *J*_H-2/H-3_ (11.4 Hz) and NOE correlations of H-2/H_3_-18 and H-3/H-14a (δ_H_ 2.98 m) suggested the *trans*-axial orientation of H-2 and H-3, and 1*E* geometry. An NOE correlation of H-2/H-15 (δ_H_ 3.43 q, *J* = 7.2 Hz) established that these two protons were on the same face of the molecule and thus H-15 had an α-orientation (Figure 3). Furthermore, the significant NOE correlations of H-11 (δ_H_ 3.79 br d, *J* = 10.8 Hz)/H-3 and H-11/H-14a, and the lack of an NOE correlation between H-11 and H_3_-20 were in agreement with the 11R* and 12S* configurations. Thus, 4 was elucidated as (11R*,12S*,15R*,1*E*,3*E*,7*E*)-11-hydroxycembra-1,3,7-trien-17,12-olide.

Locrassumin E (**5**) had a molecular formula of C_20_H_32_O_3_ as determined by HRESIMS (*m/z* 343.2247 [M + Na]^+^, calcd for C_20_H_32_O_3_Na, 343.2249) and NMR data, indicating five degrees of unsaturation (Figures S29–S35). The preliminary analysis of 1D NMR data revealed that compound **5** had a structure closely related to that of **4**. The only difference was loss of signals for a methyl doublet, an ester carbonyl, and an aliphatic methine and the appearance of signals for an additional methyl singlet (δ_H_ 1.49 s, H_3_-16) and two additional oxygenated sp^3^ carbons (δ_C_ 77.6, C, C-15; 73.6, CH_2_, C-17). Thus, **5** should be a 17-deoxo-15-hydroxy derivative of **4**. This assumption was supported by the HMBC correlations from the methyl singlet H_3_-16 to an olefinic carbon C-1 (δ_C_ 140.9, C) and the two oxygenated carbons C-15 and C-17 (Figure 2). The ether linkage between C-12 and C-17 was confirmed by HMBC correlations from the oxymethylene protons H_2_-17 (δ_H_ 3.82 d, *J* = 12.6 Hz; 3.44 d, *J* = 12.6 Hz) to the oxygenated carbon C-12 (δ_C_ 80.2, C). The geometries of C-3/C-4 and C-7/C-8 double bonds and relative configurations at C-11 and C-12 were consistent with those in **4** as indicated by the similar chemical shifts of C-18 and C-19 and compatible NOE relationships [H-3 (δ_H_ 5.96 d, *J* = 11.4 Hz)/H-5b (δ_H_ 2.01 m), H-7 (δ_H_ 5.09dd, *J* = 10.2, 4.8 Hz)/H-9a (δ_H_ 2.20 m), and H-11 (δ_H_ 3.44 br d, *J* = 10.8 Hz)/H-3], as well as the lack of NOE correlation between H_3_-20 and H-11 (Figure 3). In addition, the coupling constant value of *J*_H-2/H-3_ (11.4 Hz) and the NOE correlations of H-3/H-14a (δ_H_ 2.84 ddd, *J* = 15.0, 11.4, 6.0 Hz), H-2 (δ_H_ 6.06 dd, *J* = 11.4, 1.8 Hz)/H_3_-18 (δ_H_ 1.79 s), and H-2/H_3_-16 allowed the assignment of 1*E* and α-orientation of H_3_-16. Thus, **5** was determined as (11R*,12S*,15R*,1*E*,3*E*,7*E*)-12,17-epoxycembra-1,3,7-trien-11,15-diol.

The molecular formula of locrassumin F (**6**) was determined to be C_20_H_28_O_4_ by its HRESIMS (*m/z* 355.1883 [M + Na]^+^, calcd for C_20_H_28_O_4_Na, 355.1885) and NMR data, requiring seven degrees of unsaturation (Figures S36–S42). The NMR data of **6** (Table 1 and Table 2) were found to be very similar to those of the co-occurring analogue *ent*-sarcophine (**12**) [35]. The only difference was attributed to the absence of a tetrasubstituted double bond, while presenting an additional tetrasubstituted epoxy (δ_C_ 71.7, C, C-1; 60.7, C, C-15), indicating that **6** was probably a C-1/C-15 epoxylation derivative of **12**. The presence of an α,β-epoxy-γ-lactone was further supported by the HMBC correlations from H_3_-17 (δ_H_ 1.55 s) to the carbonyl carbon C-16 (δ_C_ 172.8) and two oxygenated carbons C-1 and C-15 and from the oxymethine proton H-2 (δ_H_ 5.29 d, *J* = 10.8 Hz) to C-16, while the substructure from C-3 to C-13 was established as identical to that in **12** based on the HMBC and COSY correlations as depicted in Figure 2. The relative configurations at C-1, C-7, and C-8, as well as the geometries of the two double bonds were assigned similarly to those in **12** by the compatible NMR data including the NOE relationships of H-2/H_3_-18 (δ_H_ 1.88 s), H-3 (δ_H_ 5.19 d, *J* = 10.8 Hz)/H-5b (δ_H_ 2.37 m), H-3/H-7 (δ_H_ 2.64 br t, *J* = 3.6 Hz), H-7/H-9b (δ_H_ 0.95 t, *J* = 13.2 Hz), H-11 (δ_H_ 5.12 dd, *J* = 9.0, 6.6 Hz)/H-13b (δ_H_ 1.99 m), and the lack of NOE correlations of H-7/H_3_-19 and H-11/H_3_-20. In addition, the NOE correlations of H-3/H-14a (δ_H_ 1.91 m) and the lack of an NOE correlation between H-2 and H_2_-14 suggested the *α*-orientation of C-1/C-15 epoxy ring. Thus, **6** was elucidated as (1R*,2R*,7R*,8R*,15R*,3*E*,11*E*)-7,8:1,15-diepoxycembra-3,11-dien-16,2-olide.

Locrassumin G (**7**) was assigned a molecular formula of C_21_H_34_O_4_ on the basis of HRESIMS (*m/z* 373.2351 [M + Na]^+^, calcd for C_21_H_34_O_4_Na, 373.2355) and NMR data, implying five degrees of unsaturation (Figures S43–S49). The ^13^C NMR spectrum showed six olefinic carbon signals (Table 2), requiring **7** to be a bicyclic molecule according to the remaining two degrees of unsaturation. A methyl-bearing dihydrofuranring was indicated by the ^13^C NMR signals at δ_C_ 132.4 (C, C-1), 127.9 (C, C-15), 84.0 (CH, C-2), 78.5 (CH_2_, C-16), 10.1 (CH_3_, C-17) and ^1^H NMR signals at δ_H_ 5.44 (br d, *J* = 9.6 Hz, H-2), 4.51 (d, *J* = 11.4 Hz, H-16a), 4.46 (d, *J* = 11.4 Hz, H-16b), 1.65 (s, H_3_-17), and further confirmed by the HMBC correlations from H_3_-17 to C-1, C-15, and C-16 and from the oxymethylene protons H_2_-16 to C-2 (Figure 2). The COSY correlation between H-2 and an olefinic proton H-3 (δ_H_ 5.08 d, *J* = 9.6 Hz) revealed that C-3/C-4 was located by a double bond. The other two olefinic carbons at δ_C_ 138.9 (CH, C-11) and 124.4 (CH, C-10) and two olefinic proton signals at δ_H_ 5.53 (m, H-10) and 5.52 (d, *J* = 18.6 Hz, H-11) were attributed to a 1,2-disubstituted double bond. These NMR data were similar to those of the known cembranoid (2S*,7S*,8S*,12R*,1*Z*,3*E*,10*E*)-7,8:2,16-diepoxycembra-1(15),3,10-trien-12-ol [27]. The difference arose from the absence of a trisubstituted epoxy in **7**. The HMBC correlations from H_3_-19 (δ_H_ 1.15 s) to two oxygenated carbons C-7 (δ_C_ 71.2, CH) and C-8 (δ_C_ 78.4, C) and a methylene carbon C-9 (δ_C_ 36.7, CH_2_) and from the methoxy protons (δ_H_ 3.23 s, H_3_-21) to C-8 disclosed that C-7 and C-8 were substituted by a hydroxy and a methoxy group, respectively. Further HMBC correlations from H_3_-20 (δ_H_ 1.33 s) to an olefinic carbon C-11, an oxygenated carbon C-12 (δ_C_ 73.0, C), and a methylene carbon C-13 (δ_C_ 41.3, CH_2_) confirmed the location of a double bond at C-10/C-11 and C-12 was hydroxylated. The coupling constant value of *J*_H-2/H-3_ (9.6 Hz) and the NOE correlations of H-2/H_3_-18 (δ_H_ 1.74 s) and H-3/H-5a (δ_H_ 2.32 m) suggested the *trans*-axial orientation of H-2 and H-3 and 3*E*, which was also implied by the chemical shifts of C-18 (<20 ppm) [23,24], while the coupling constant value of *J*_H-10/H-11_ (18.6 Hz) indicated the 10*E* geometry. In addition, NOE correlations of H-3/H-14b (δ_H_ 1.69 m), H-7 (δ_H_ 3.34 d, *J* = 10.8 Hz)/H-14b, H-7/H-10, and H_3_-19/H-10, H_3_-20/H-10, and H_3_-20/H-14a (δ_H_ 2.14 m) were in agreement with the relative configurations of 7R*, 8S*, and 12S* (Figure 3). Thus, **7** was defined as (2R*,7R*,8S*,12S*,1*Z*,3*E*,10*E*)-8-methoxy-2,16-epoxycembra-1(15),3,10-trien-7,12-diol.

The spectroscopic data analysis and comparison of NMR and HRESIMS data revealed that the structures of compounds **8**–**10** were identical to the known cembranoids laevigatol B [36], (+)-isosarcophine [37], and (+)-7*S*,8*R*-dihydroxydeepoxysarcophytoxide [38], respectively. However, the antipodal specific rotations of **8**–**10** (${\left[\alpha \right]}_{D}^{25}$ −17 (*c* 0.10, CHCl_3_); −263 (*c* 0.10, CHCl_3_); −99 (*c* 0.10, CHCl_3_), respectively) in comparison with those of the three known analogues (${\left[\alpha \right]}_{D}^{25}$ +7.7 (*c* 1.00, CH_2_Cl_2_); +235.3 (*c* 0.14, CHCl_3_); +140.0 (*c* 0.48, CHCl_3_), respectively) suggested **8**–**10** to be enantiomers of the previously reported analogues, and named (–)-laevigatol B, (–)-isosarcophine, and (–)-7*R*,8*S*-dihydroxydeepoxysarcophytoxide, respectively.

Eight known compounds were also isolated from the *L. crassum* extract and identified as (–)-sarcophytoxide (**11**) [39], *ent*-sarcophine (**12**) [35], sarcophytonolide O (**13**) [30], sartrolide G (**14**) [40], emblide (**15**) [41], sarcrassin D (**16**) [28], ketoemblide (**17**) [42], and methyl sarcotroate B (**18**) [2], by comparison of their ^1^H and ^13^C NMR, MS spectroscopic data (Figures S50–S68), and specific rotations, with those reported in the literature.

All compounds were tested for their *in vitro* anti-inflammatory activities [43]. Nitric oxide (NO) is an important signaling molecule that is involved in the regulation of diverse physiological and pathological processes. Overproduction of NO is associated with various human diseases, particularly acute and chronic inflammation, while the level of NO can reflect the degree of inflammation. In the primary assay, compounds **1**, **7**, **12**, **13**, and **17** showed moderate inhibition against lipopolysaccharide-induced NO production in mouse peritoneal macrophages with IC_50_ values of 8–24 μM, whereas no inhibitory effect was observed for the other compounds (IC_50_ > 30 μM) (Table 3).

## 3. Materials and Methods

### 3.1. General Experimental Procedures

^1^H and ^13^C NMR spectra were acquired with a Bruker Avance-600FT NMR spectrometer (Bruker, Munich, Germany) using TMS as an internal standard. HRESIMS data were recorded using a Thermo Scientific Q Exactive hybrid quadrupole-Orbitrap mass spectrometer (Thermo Fisher Scientific, Waltham, MA, USA). UV spectra were measured with a TU 1901 spectrometer (Puxi Ltd., Beijing, China). IR spectra were obtained using a Bruker Equinox 55 spectrometer (Bruker, Munich, Germany). Optical rotations were measured with a PoLAAR 3005 digital polarimeter (Optical Activity Ltd., Cambridgeshire, UK). Silica gel (200–300 mesh, Qingdao Marine Chemistry Co. Ltd., Qingdao, China), Sephadex LH-20 (GE Healthcare Bio-sciences AB, Uppsala, Sweden), and ODS (50 μm, YMC, Tokyo, Japan) were used for column chromatography. TLC analysis was carried out using silica gel GF_254_ (Qingdao Marine Chemistry Co. Ltd., Qingdao, China). Semipreparative HPLC was performed using an Agilent 1100 series instrument equipped with a VWD G1314A detector (Agilent, Palo Auto, Santa Clara, CA, USA) and a YMC-Pack C_18_ column (10 μm, 250 × 10 mm, YMC, Tokyo, Japan).

### 3.2. Animal Material

Specimens of the soft coral *Lobophytum crassum* von Marenzeller, 1886 were collected from the inner coral reef of Meishan, Hainan Province, China, in April 2014, at a depth of 6 m and frozen immediately after collection. The identification was carried out by one of the authors (X.L.). A voucher specimen (HS201404) was deposited at the Laboratory of Marine Natural Products Chemistry, Wenzhou Medical University, China.

### 3.3. Extraction and Isolation

The frozen soft coral *Lobophytum crassum* (wet weight: 1.06 kg) was homogenized and extracted with 95% EtOH at room temperature (r.t.). The concentrated extract was partitioned between EtOAc and H_2_O. Evaporation of EtOAc *in vacuo* afforded a dark residue of 30.0 g. The EtOAc fraction (15.0 g) was subjected to silica gel vacuum column chromatography, eluting with a gradient of EtOAc/petroleum ether (1:30, 1:10, 1:5, 1:3, and 1:2), to yield seven fractions (A1–A7). Fraction A3 (300.5 mg) was further fractionated on a silica gel column, eluting with a gradient of acetone/petroleum ether (1:15 and 1:10), to afford three fractions (A3a–A3c). Fraction A3b (30.5 mg) was purified by semipreparative HPLC, using MeOH/H_2_O (75:25) as eluent, to afford **9** (2.2 mg). Fraction A3c (62.1 mg) was purified by HPLC, eluting with MeOH/H_2_O (70:30), to yield **11** (15.8 mg). Fraction A4 (2.1 g) was chromatographed on a Sephadex LH-20 column, using CH_2_Cl_2_/MeOH (1:1) as a mobile phase, to obtain three fractions (A4a–A4c). Fraction A4b (1.1 g) was further subjected to an ODS column, eluting with a gradient of MeOH/H_2_O (70:30, 75:25, 80:20, 85:15, and 90:10), to afford six fractions (A4b1–A4b6). Fraction A4b1 (100.3 mg) was purified by HPLC (MeOH/H_2_O, 65:35) to obtain **6** (5.6 mg) and **12** (17.4 mg). In the same manner, fractionsA4b2 (120.0 mg) and A4b3 (98.2 mg) were eluted with MeOH/H_2_O (70:30) to yield **14** (6.0 mg), **17** (18.7 mg), **15** (9.1 mg), **4** (3.8 mg), and **16** (6.7 mg), while fraction A4b4 (103.5 mg) was purified with MeOH/H_2_O (75:25) to afford **1** (7.0 mg), **2** (8.0 mg), and **13** (28.7 mg). Fraction A5 (620.7 mg) was separated on a Sephadex LH-20 column (CH_2_Cl_2_/MeOH, 1:1) to yield three fractions (A5a–A5c). Fraction A5b (271.4 mg) was further fractionated on an ODS column, eluting with MeOH/H_2_O (70:30 and 75:25), to afford three fractions (A5b1–A5b3). Fraction A5b1 (58.9 mg) was purified by HPLC (MeOH/H_2_O, 65:35) to obtain **8** (3.3 mg) and **18** (9.6 mg). In the same manner, fraction A5b3 (16.8 mg) was eluted with MeOH/H_2_O (60:40) to afford **5** (3.2 mg). Fraction A6 (421.5 mg) was fractionated on a Sephadex LH-20 column (CH_2_Cl_2_/MeOH, 1:1) to yield four fractions (A6a–A6d). Fraction A6b (37.2 mg) was purified by HPLC (MeOH/H_2_O, 70:30) to afford **7** (3.5 mg) and **3** (3.8 mg). Following the same protocol as for fraction A6b, **10** (5.6 mg) was separated from fraction A6c (26.1 mg).

*Locrassumin A* (**1**)*:* colorless oil; ${\left[\alpha \right]}_{D}^{25}$ +9 (*c* 0.10, CHCl_3_); UV (MeOH) λ_max_ (log ε) 212 (4.08), 284 (4.25); IR (KBr) ν_max_ 2967, 2870, 1708, 1624, 1436, 1384, 1263, 1198, 1109, 1067, 866, 762 cm^−1^; ^1^H and ^13^C NMR data, Table 1 and Table 2; HRESIMS *m/z* 399.2134 [M + Na]^+^ (calcd. for C_22_H_32_O_5_Na, 399.2147).

*Locrassumin B* (**2**): colorless oil; ${\left[\alpha \right]}_{D}^{25}$ +385 (*c* 0.10, CHCl_3_); UV (MeOH) λ_max_ (log ε) 214 (4.05), 284 (4.22); IR (KBr) ν_max_ 2955, 2872, 1708, 1631, 1435, 1384, 1264, 1191, 1121, 1070 cm^−1^; ^1^H and ^13^C NMR data, Table 1 and Table 2; HRESIMS *m/z* 399.2135 [M + Na]^+^ (calcd. for C_22_H_32_O_5_Na, 399.2147).

*Locrassumin C* (**3**): colorless oil; ${\left[\alpha \right]}_{D}^{25}$ +44 (*c* 0.10, CHCl_3_); IR (KBr) ν_max_ 3450, 2966, 2871, 1726, 1459, 1379, 1222 cm^−1^; ^1^H and ^13^C NMR data, Table 1 and Table 2; HRESIMS *m/z* 417.2251 [M + Na]^+^ (calcd. for C_22_H_34_O_6_Na, 417.2253).

*Locrassumin D* (**4**): colorless oil; ${\left[\alpha \right]}_{D}^{25}$ −50 (*c* 0.06, CHCl_3_); UV (MeOH) λ_max_ (log ε) 204 (3.55), 243 (3.21); IR (KBr) ν_max_ 3442, 2930, 1707, 1646, 1460, 1383, 1081 cm^−1^; ^1^H and ^13^C NMR data, Table 1 and Table 2; HRESIMS *m/z* 341.2094 [M + Na]^+^ (calcd. for C_20_H_30_O_3_Na, 341.2093).

*Locrassumin E* (**5**): colorless oil; ${\left[\alpha \right]}_{D}^{25}$ +127 (*c* 0.06, CHCl_3_); UV (MeOH) λ_max_ (log ε) 203 (3.96), 251 (4.13); IR (KBr) ν_max_ 3449, 2924, 2858, 1742, 1446, 1378, 1271, 1085, 917, 873 cm^−1^; ^1^H and ^13^C NMR data, Table 1 and Table 2; HRESIMS *m/z* 343.2247 [M + Na]^+^ (calcd. for C_20_H_32_O_3_Na, 343.2249).

*Locrassumin F* (**6**): colorless oil; ${\left[\alpha \right]}_{D}^{25}$ −16 (*c* 0.10, CHCl_3_); IR (KBr) ν_max_ 2930, 1777, 1451, 1383, 1320, 1243, 1104, 975 cm^−1^; ^1^H and ^13^C NMR data, Table 1 and Table 2; HRESIMS *m/z* 355.1883 [M + Na]^+^ (calcd. for C_20_H_28_O_4_Na, 355.1885).

*Locrassumin G* (**7**): colorless oil; ${\left[\alpha \right]}_{D}^{25}$ −48 (*c* 0.07, CHCl_3_); IR (KBr) ν_max_ 3446, 2931, 2860, 1452, 1377, 1272, 1090, 979 cm^−1^; ^1^H and ^13^C NMR data, Table 1 and Table 2; HRESIMS *m/z* 373.2351 [M + Na]^+^ (calcd. for C_21_H_34_O_4_Na, 373.2355).

(*–*)*-Laevigatol B* (**8**): colorless oil; ${\left[\alpha \right]}_{D}^{25}$ −17 (*c* 0.10, CHCl_3_); IR (KBr) ν_max_ 3435, 2928, 2858, 1662, 1450, 1384, 1031 cm^−1^; ^1^H NMR (600 MHz, CDCl_3_) δ 5.23 (1H, t, *J* = 1.8 Hz, H-17a), 5.16 (1H, d, *J* = 10.2 Hz, H-3), 5.10 (1H, t, *J* = 1.8 Hz, H-17b), 5.06 (1H, t, *J* = 7.8 Hz, H-11), 4.78 (1H, d, *J* = 10.2 Hz, H-2), 4.64 (1H, dt, *J* = 13.2, 1.8 Hz, H-16a), 4.45 (1H, dt, *J* = 13.2, 1.8 Hz, H-16b), 2.69 (1H, t, *J* = 4.2 Hz, H-7), 2.32 (1H, m, H-13a), 2.30 (2H, m, H_2_-5), 2.27 (1H, m, H-10a), 2.17 (1H, m, H-14a), 2.12 (1H, ddd, *J* = 12.6, 4.2, 3.0 Hz, H-9a), 1.94 (1H, m, H-10b), 1.83 (1H, m, H-6a), 1.83 (3H, s, H_3_-18), 1.81 (1H, m, H-13b), 1.65 (1H, m, H-6b), 1.60 (3H, s, H_3_-20), 1.26 (1H, m, H-14b), 1.26 (3H, s, H_3_-19), 0.96 (1H, td, *J* = 13.2, 3.0 Hz, H-9b); ^13^C NMR (150 MHz, CDCl_3_) δ 152.5 (C, C-15), 139.4 (C, C-4), 136.2 (C, C-12), 123.4 (CH, C-11), 121.6 (CH, C-3), 106.2 (CH_2_, C-17), 84.5 (CH, C-2), 81.2 (C, C-1), 69.0 (CH_2_, C-16), 62.1 (CH, C-7), 59.8 (C, C-8), 40.0 (CH_2_, C-9), 37.6 (CH_2_, C-5), 34.4 (CH_2_, C-13), 32.7 (CH_2_, C-14), 25.5 (CH_2_, C-6), 23.7 (CH_2_, C-10), 16.6 (CH_3_, C-19), 16.3 (CH_3_, C-18), 15.2 (CH_3_, C-20); HRESIMS *m/z* 341.2090 [M + Na]^+^ (calcd. for C_20_H_30_O_3_Na, 341.2093).

(*–*)*-Isosarcophine* (**9**): colorless oil; ${\left[\alpha \right]}_{D}^{25}$ −263 (*c* 0.10, CHCl_3_); UV (MeOH) λ_max_ (log ε) 204 (4.24); IR (KBr) ν_max_ 2924, 2854, 1754, 1676, 1446, 1384, 1287, 1089, 998 cm^−1^; ^1^H NMR (600 MHz, CDCl_3_) δ 5.45 (1H, d, *J* = 9.6 Hz, H-2), 4.98 (1H, d, *J* = 9.0 Hz, H-7), 4.85 (1H, d, *J* = 9.6 Hz, H-3), 2.54 (1H, dd, *J* = 10.2, 2.4 Hz, H-11), 2.54 (1H, m, H-14a), 2.46 (1H, m, H-6a), 2.37 (1H, m, H-5a), 2.32 (1H, m, H-9a), 2.22 (1H, m, H-5b), 2.13 (1H, m, H-6b), 2.10 (1H, m, H-10a), 2.10 (1H, m, H-14b), 2.00 (1H, m, H-13a), 1.99 (1H, m, H-9b), 1.85 (3H, s, H_3_-17), 1.68 (3H, s, H_3_-18), 1.68 (3H, s, H_3_-19), 1.32 (3H, s, H_3_-20), 1.26 (1H, m, H-10b), 1.05 (1H, m, H-13b); ^13^C NMR (150 MHz, CDCl_3_) δ 174.6 (C, C-16), 161.1 (C, C-1), 144.9 (C, C-4), 133.7 (C, C-8), 125.2 (CH, C-7), 123.4 (C, C-15), 120.6 (CH, C-3), 78.4 (CH, C-2), 62.0 (CH, C-11), 60.8 (C, C-12), 38.7 (CH_2_, C-5), 37.1 (CH_2_, C-13), 36.7 (CH_2_, C-9), 24.2 (CH_2_, C-10), 23.9 (CH_2_, C-6), 23.6 (CH_2_, C-14), 15.9 (CH_3_, C-20), 15.1 (CH_3_, C-18), 14.8 (CH_3_, C-19), 8.8 (CH_3_, C-17); HRESIMS *m/z* 339.1931 [M + Na]^+^ (calcd. for C_20_H_28_O_3_Na, 339.1936).

(*–*)*-7R,8S-Dihydroxydeepoxysarcophytoxide* (**10**): colorless oil; ${\left[\alpha \right]}_{D}^{25}$ −99 (*c* 0.10, CHCl_3_); IR (KBr) ν_max_ 3443, 2925, 2857, 1446, 1379, 1000, 945 cm^−1^; ^1^H NMR (600 MHz, CDCl_3_) δ 5.54 (1H, m, H-2), 5.14 (1H, d, *J* = 10.2 Hz, H-3), 4.92 (1H, dd, *J* = 9.6, 3.6 Hz, H-11), 4.50 (2H, s, H_2_-16), 3.57 (1H, d, *J* = 10.8 Hz, H-7), 2.53 (1H, ddd, *J* = 13.8, 10.8, 8.4 Hz, H-14a),2.39 (H, td, *J* = 12.6, 2.4 Hz, H-5a), 2.23 (1H, m, H-10a), 2.18 (1H, m, H-5b), 2.11 (1H, m, H-10b), 1.93 (2H, m, H_2_-13),1.87 (1H, m, H-6a), 1.85 (3H, s, H_3_-18), 1.81 (1H, m, H-9a), 1.70 (1H, m, H-9b), 1.68 (1H, m, H-14b), 1.64 (3H, s, H_3_-17), 1.63 (3H, s, H_3_-20), 1.51 (1H, m, H-6b), 1.19 (3H, s, H_3_-19); ^13^C NMR (150 MHz, CDCl_3_) δ 139.1 (C, C-4), 135.9 (C, C-12), 133.3 (C, C-1), 127.9 (C, C-15), 126.8 (CH, C-3), 124.2 (CH, C-11), 84.0 (CH, C-2), 78.5 (CH_2_, C-16), 75.5 (C, C-8), 72.9 (CH, C-7), 37.0 (CH_2_, C-9), 36.7 (CH_2_, C-13), 35.7 (CH_2_, C-5), 26.7 (CH_2_, C-6), 25.3 (CH_2_, C-14), 24.3 (CH_3_, C-19), 23.7 (CH_2_, C-10), 16.0 (CH_3_, C-18), 15.4 (CH_3_, C-20), 10.2 (CH_3_, C-17); HRESIMS *m/z* 343.2240 [M + Na]^+^ (calcd. for C_20_H_32_O_3_Na, 343.2249).

### 3.4. Assay for Inhibition of Nitric Oxide Production

A previously established protocol [43] was followed except that 30 mM dexamethasone in DMSO was used as the positive control and each test compound (30 mM in DMSO) was diluted to 1–30 μM at r.t. before the experiment.

## 4. Conclusions

This is a further chemical examination on the soft coral *Lobophytum crassum* from the South China Sea, which presents ten new cembrane-based diterpenes (**1**–**10**) to enrich the chemical diversity of secondary metabolites from *Lobophytum* species. Compound **3** possesses an unprecedented tetradecahydrobenzo[3,4]cyclobuta[1,2][8]annulene skeleton which could be derived from **1**, **2**, or **13**. Compounds **1**, **7**, **12**, **13**, and **17** exhibited moderate inhibition against lipopolysaccharide-induced NO production in mouse peritoneal macrophages with IC_50_ values of 8–24 μM. These results add to a growing class of diverse cembranoid structures that are known to inhibit NO production.

## Figures and Tables

**Table 1:** ^1^H NMR data for **1**–**7** (CDCl_3_, 600 MHz) ^a^*.*

No.	1	2	3	4	5	6	7
2	6.24, d (12.0)	6.23, d (12.0)	5.31, d (3.0)	6.02, dd (11.4, 1.8)	6.06, dd (11.4, 1.8)	5.29, d (10.8)	5.44, br d (9.6)
3	7.70, d (12.0)	7.62, d (12.0)	3.17, d (3.0)	5.87, d (11.4)	5.96, d (11.4)	5.19, d (10.8)	5.08, d (9.6)
5	2.63, m	2.66, m	1.92, m	2.28, m	2.26, m	2.40, m	2.32, m
	2.55, m	2.63, m	1.75, m	1.98, m	2.01, m	2.37, m	2.15, m
6	2.07, m	1.91, m	1.74, m	2.27, m	2.28, m	1.95, m	1.86, td (13.8, 3.0)
	1.57, m	1.71, m	1.57, m	2.04, m	2.03, m	1.92, m	
							1.35, m
7	2.87, dd (9.6, 3.0)	2.64, m	3.58, d (10.8)	5.11, dd (10.2, 5.4)	5.09, dd (10.2, 4.8)	2.64, br t (3.6)	3.34, d (10.8)
9	1.82, m	2.05, m	1.97, m	2.25, m	2.20, m	2.15, m	2.31, m
	1.49, m	1.19, m	1.66, m	2.15, m	2.10, m	0.95, t (13.2)	
10	2.18, m	2.12, m	1.87, m	1.82, m	1.82, m	2.28, m	5.53, m
	2.15, m	2.00, m	1.69, m	1.35, m	1.20, m	1.90, m	
11	6.80, t (7.2)	6.60, dd (7.2, 4.2)	3.24, dd (7.2, 5.4)	3.79, br d (10.8)	3.44, br d (10.8)	5.12, dd (9.0, 6.6)	5.52, d (18.6)
13	2.63, m	2.69, m	2.08, m	2.26, m	2.08, m	2.29, m	1.67, m
	2.42, m	2.43, m	1.78, m	1.94, m	1.95, ddd (15.0, 6.0, 2.4)	1.99, m	1.53, m
						
						
14	2.42, m	2.83, m	1.94, m	2.98, m	2.84, ddd (15.0, 11.4, 6.0)	1.91, m	2.14, m
	2.28, m	2.37, m	1.72, m	2.17, m		1.71, m	1.69, m
						
					2.26, m		
15	3.20, m	2.17, m	2.22, m	3.43, q (7.2)			
16	1.00, d (6.6)	1.00, d (6.6)	1.00, d (6.6)	1.47,d (7.2)	1.49, s		4.51, d (11.4)
							4.46, d (11.4)
17	0.95, d (6.6)	1.08, d (6.6)	1.01, d (6.6)		3.82, d (12.6)	1.55, s	1.65, s
					3.44, d (12.6)		
18				1.79, s	1.79, s	1.88, s	1.74, s
19	1.18, s	1.12, s	1.14, s	1.30, s	1.34, s	1.28, s	1.15, s
20				1.28, s	0.99, s	1.58, s	1.33, s
21	3.77, s	3.77, s	3.76, s				3.23, s
22	3.75, s	3.76, s	3.68, s				

^a^ The coupling constants (*J*) are in parentheses and reported in Hz; chemical shifts are given in ppm.

**Table 2:** ^13^C NMR data for **1**–**7** (CDCl_3_, 150 MHz) ^a^*.*

No.	1	2	3	4	5	6	7
1	158.8, C	158.7, C	148.3, C	133.7, C	140.9, C	71.7, C	132.4, C
2	120.4, CH	118.8, CH	113.8, CH	125.7, CH	120.4, CH	78.0, CH	84.0, CH
3	134.3, CH	136.6, CH	42.0, CH	121.7, CH	122.8, CH	119.8, CH	126.8, CH
4	127.9, C	127.7, C	50.0, C	138.2, C	137.3, C	144.4, C	139.6, C
5	23.2, CH_2_	23.6, CH_2_	24.4, CH_2_	40.4, CH_2_	40.5, CH_2_	37.7, CH_2_	35.6, CH_2_
6	27.2, CH_2_	26.8, CH_2_	29.9, CH_2_	26.4, CH_2_	26.3, CH_2_	25.3, CH_2_	26.2, CH_2_
7	60.7, CH	62.7, CH	73.7, CH	125.0, CH	124.9, CH	61.7, CH	71.2, CH
8	60.9, C	61.1, C	74.9, C	136.3, C	136.5, C	59.7, C	78.4, C
9	36.2, CH_2_	36.3, CH_2_	39.3, CH_2_	35.5, CH_2_	36.3, CH_2_	40.0, CH_2_	36.7, CH_2_
10	24.3, CH_2_	24.0, CH_2_	20.4, CH_2_	26.3, CH_2_	27.0, CH_2_	23.7, CH_2_	124.4, CH
11	142.5, CH	144.4, CH	43.5, CH	67.9, CH	70.8, CH	124.5, CH	138.9, CH
12	132.4, C	130.1, C	45.2, C	87.0, C	80.2, C	135.2, C	73.0, C
13	27.7, CH_2_	27.0, CH_2_	25.3, CH_2_	32.9, CH_2_	30.9, CH_2_	34.8, CH_2_	41.3, CH_2_
14	30.0, CH_2_	28.9, CH_2_	23.2, CH_2_	22.1, CH_2_	22.6, CH_2_	27.0, CH_2_	21.8, CH_2_
15	29.8, CH	36.8, CH	35.6, CH	51.0, CH	77.6, C	60.7, C	127.9, C
16	21.2, CH_3_	22.3, CH_3_	21.4, CH_3_	16.3, CH_3_	28.0, CH_3_	172.8, C	78.5, CH_2_
17	20.4, CH_3_	20.8, CH_3_	20.7, CH_3_	177.8, C	73.6, CH_2_	9.9, CH_3_	10.1, CH_3_
18	168.8, C	168.5, C	177.4, C	16.5, CH_3_	16.3, CH_3_	16.2, CH_3_	15.5, CH_3_
19	17.9, CH_3_	16.9, CH_3_	21.1, CH_3_	14.8, CH_3_	14.9, CH_3_	16.7, CH_3_	17.8, CH_3_
20	168.0, C	167.8, C	176.7, C	23.1, CH_3_	19.0, CH_3_	14.8, CH_3_	26.9, CH_3_
21	51.8, CH_3_	51.7, CH_3_	52.1, CH_3_				49.2, CH_3_
22	51.8, CH_3_	51.8, CH_3_	52.0, CH_3_				

^a^ The assignments were based on HMQC, HMBC, and COSY spectra.

**Table 3:** Inhibitory Activity against LPS-Induced NO Production ^a^.

Compound	IC_50_ (μM)	CC_50_^b^ (μM)
**1**	17 ± 3	>60.0
**7**	13 ± 2	>60.0
**12**	24 ± 2	>60.0
**13**	8 ± 1	>60.0
**17**	12 ± 2	>60.0

^a^ The other compounds were inactive at 30 μM; ^b^ CC_50_: cytotoxicity against mouse peritoneal macrophages.

## Supplementary Materials

The following are available online at www.mdpi.com/1660-3397/14/6/111/s1, Figures S1–S68: ^1^H,^13^C NMR and MS spectroscopic data of compounds **1**–**10**.
