# Supplementary material for: Cembranoids from a Chinese Collection of the Soft Coral Lobophytum crassum
Source: Mar Drugs. 2016 Jun 3;14(6):111. doi: 10.3390/md14060111 (PMC4926070; doi:10.3390/md14060111)
Supplement: Supplementary file 1 [file marinedrugs-14-00111-s001.pdf]

Supplementary Materials: Cembranoids from a Chinese Collection of the Soft Coral *Lobophytum crassum*

Min Zhao, Shimiao Cheng, Weiping Yuan, Yiyuan Xi, Xiubao Li, Jianyong Dong, Kexin Huang, Kirk R. Gustafson and Pengcheng Yan

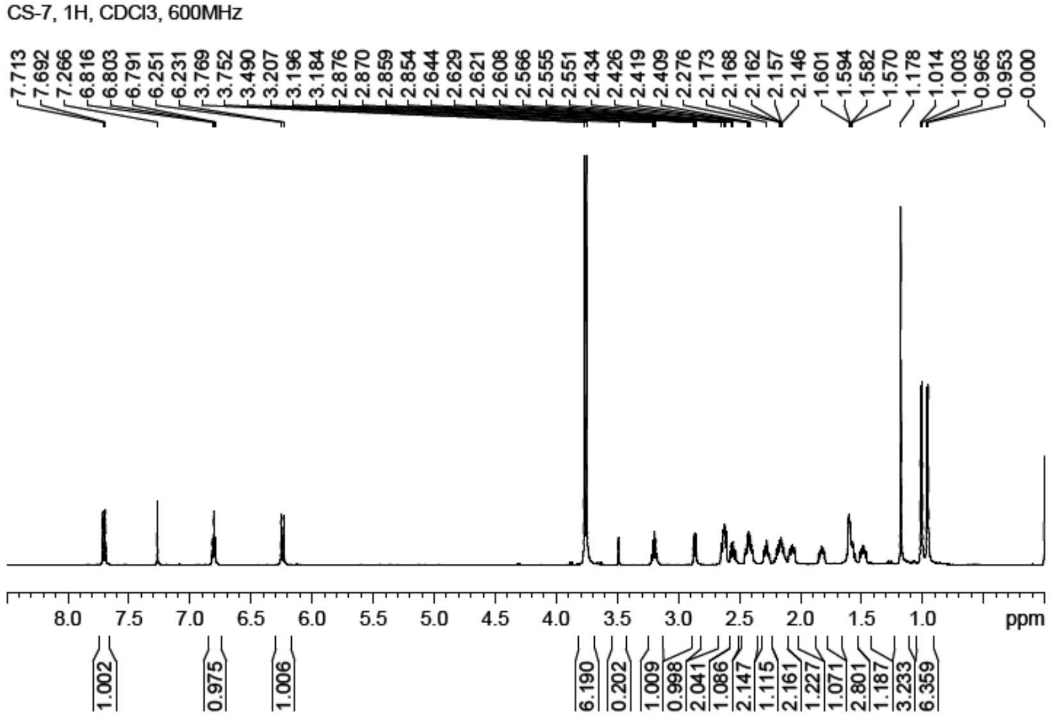

Figure S1. ^{1}H NMR spectrum (600 MHz) of compound **1** in $CDCl_3$.

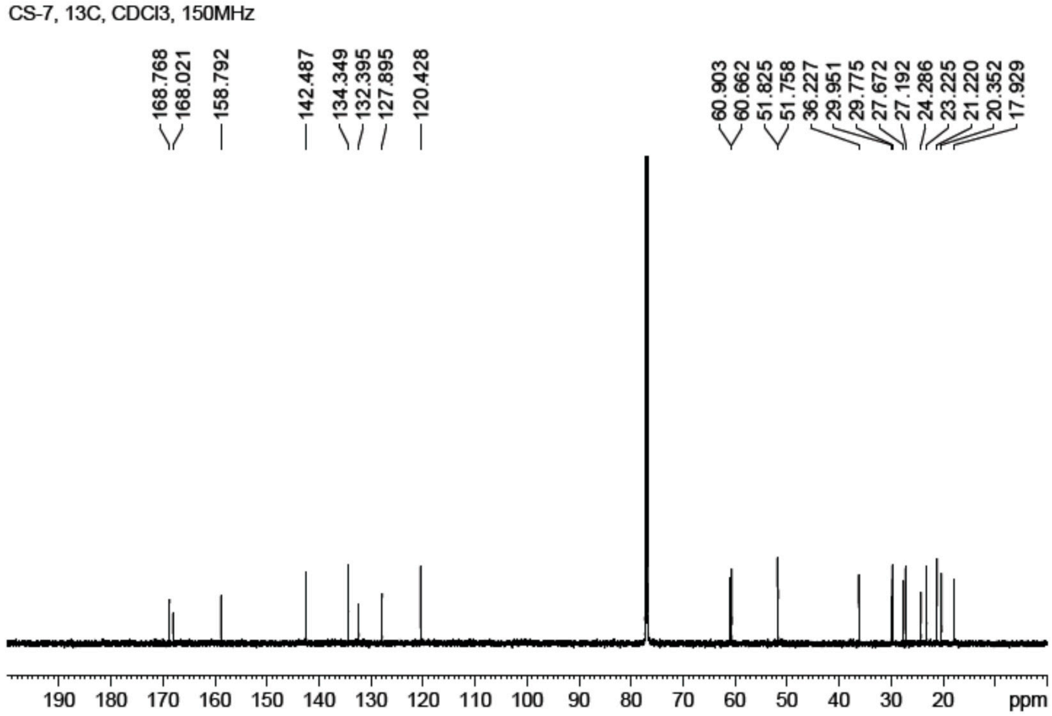

Figure S2. ^{13}C NMR spectrum (150 MHz) of compound **1** in $CDCl_3$.

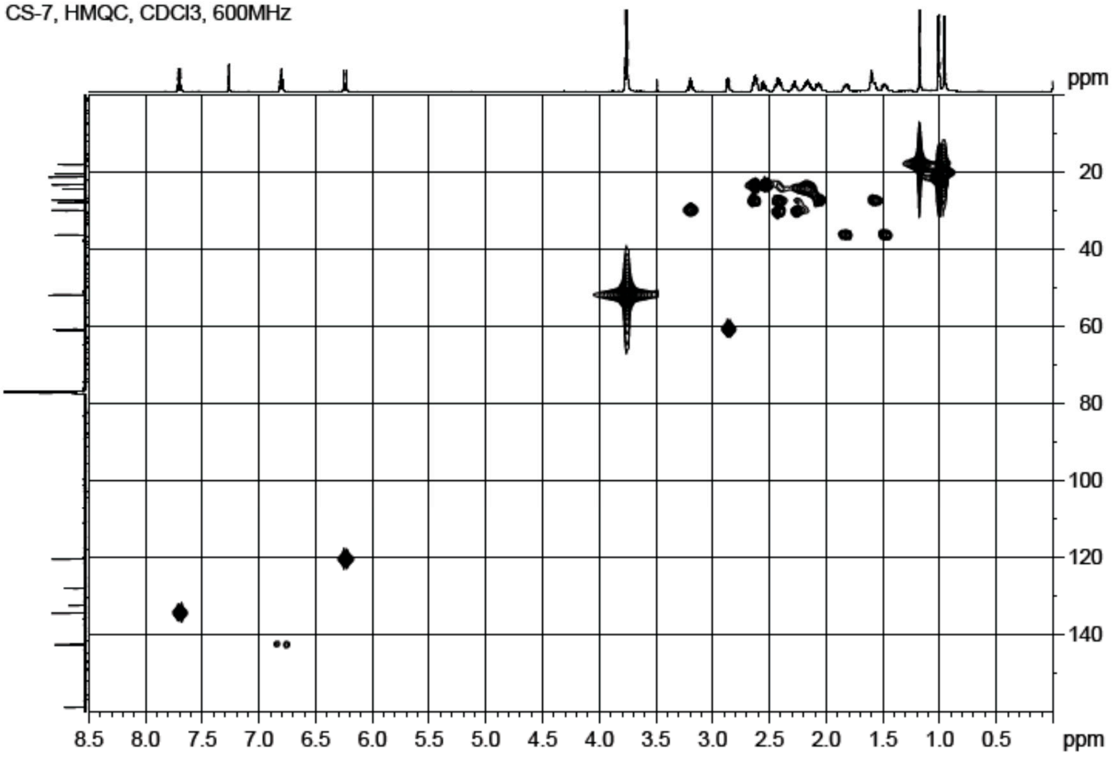

Figure S3. HMQC spectrum of compound **1** in $CDCl_3$.

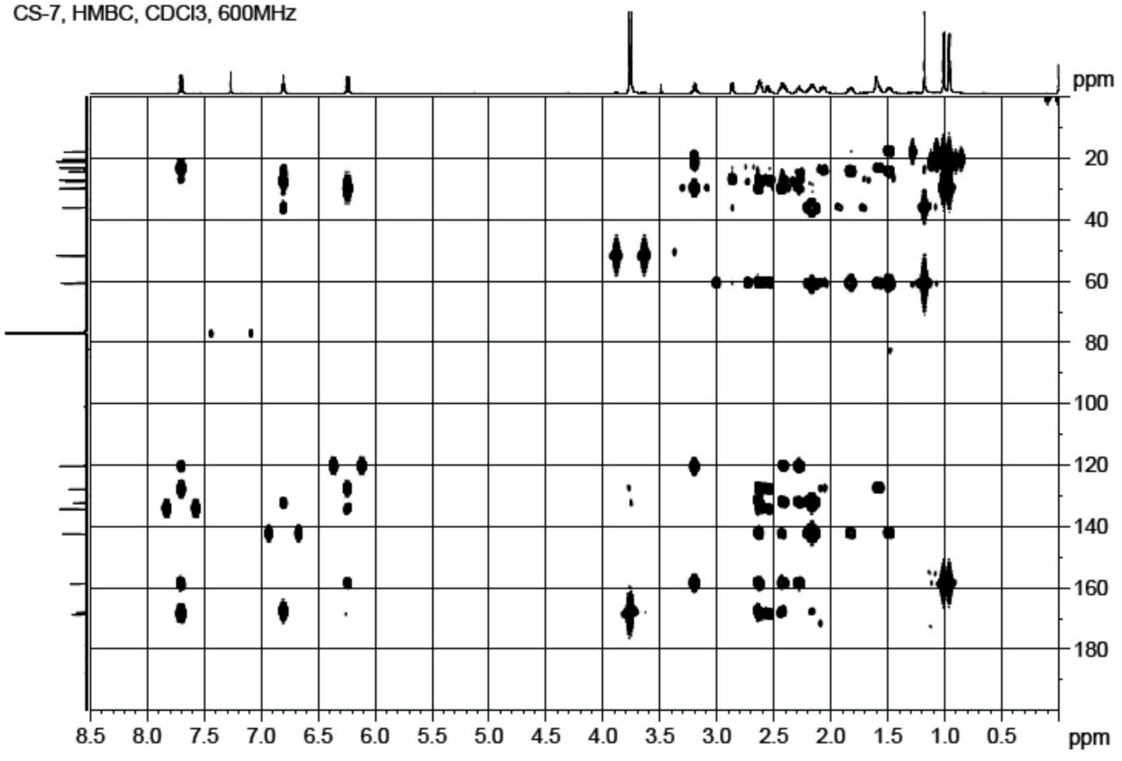

Figure S4. HMBC spectrum of compound **1** in $CDCl_3$.

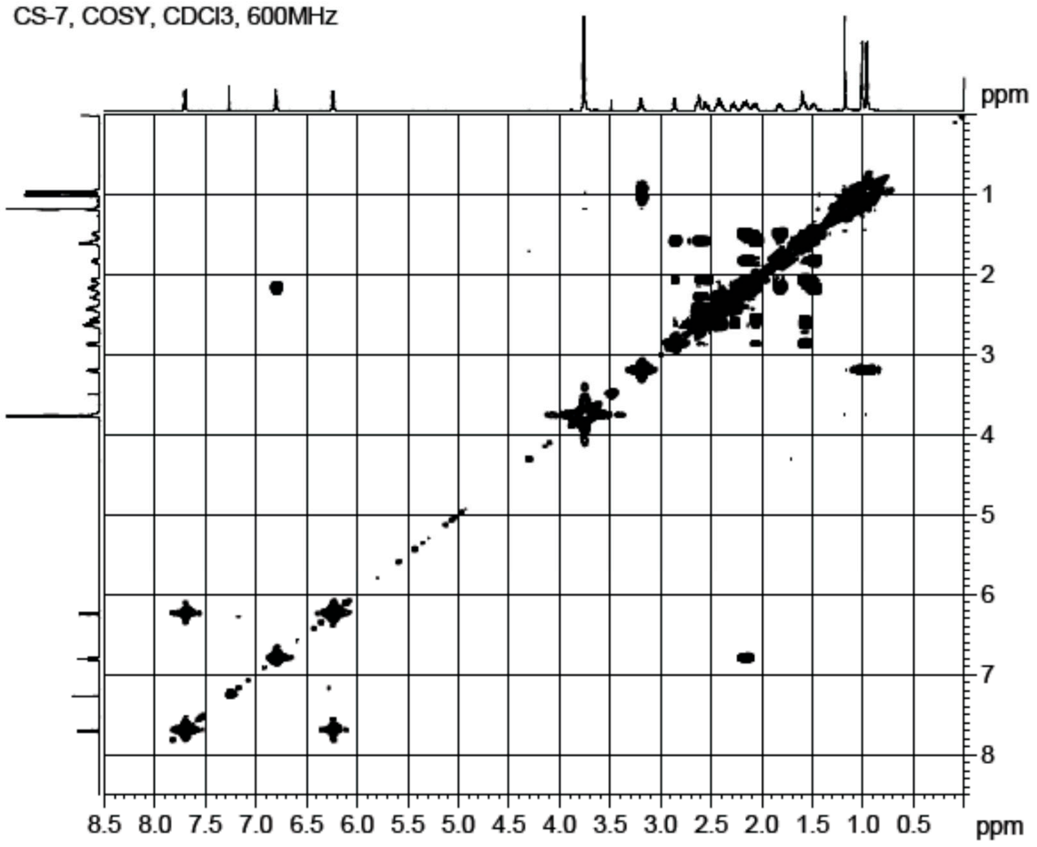

Figure S5. COSY spectrum of compound **1** in $CDCl_3$.

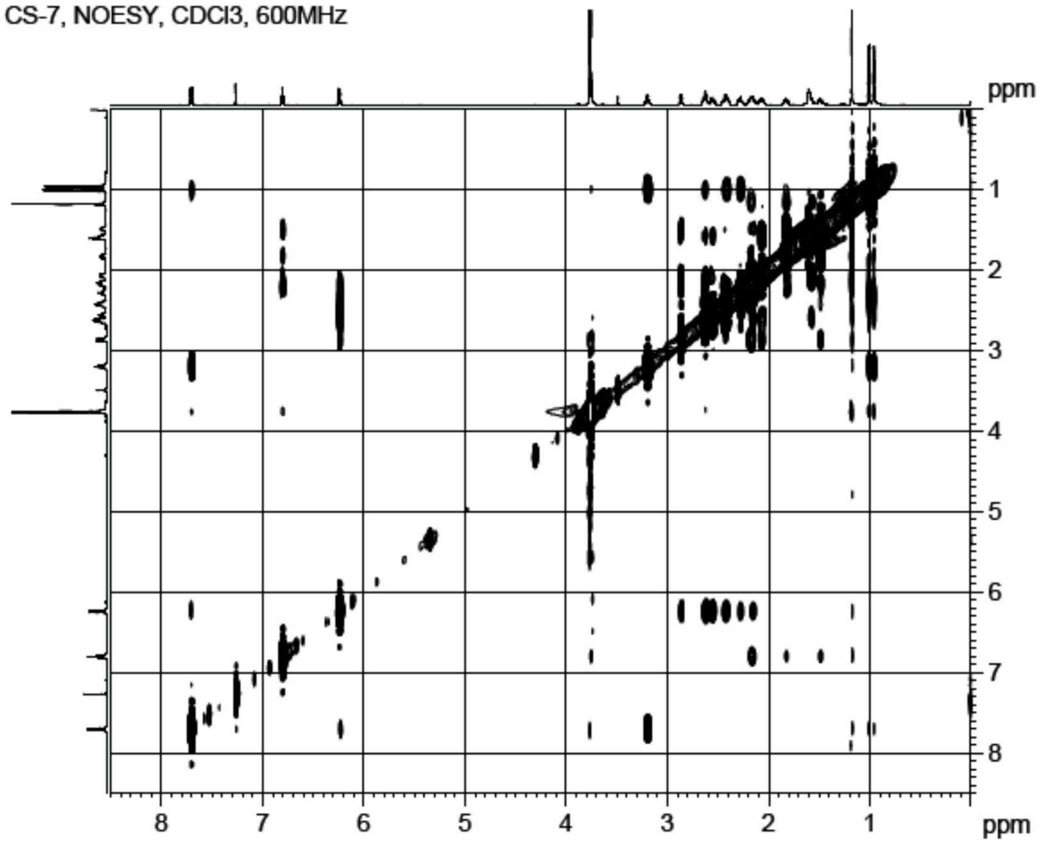

Figure S6. NOESY spectrum of compound **1** in $CDCl_3$.

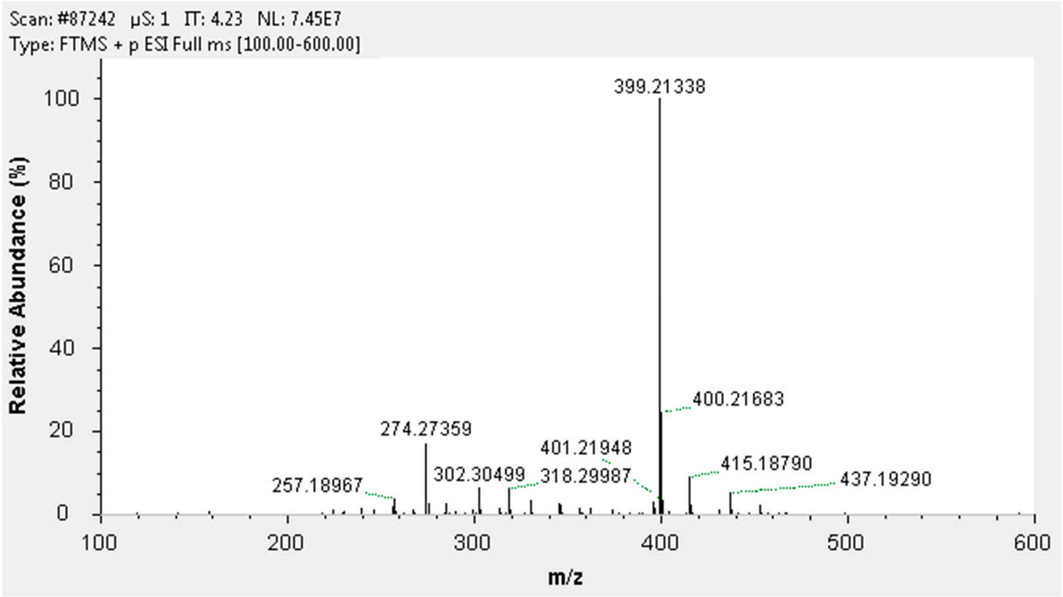

Figure S7. HRESIMS spectrum of compound **1**.

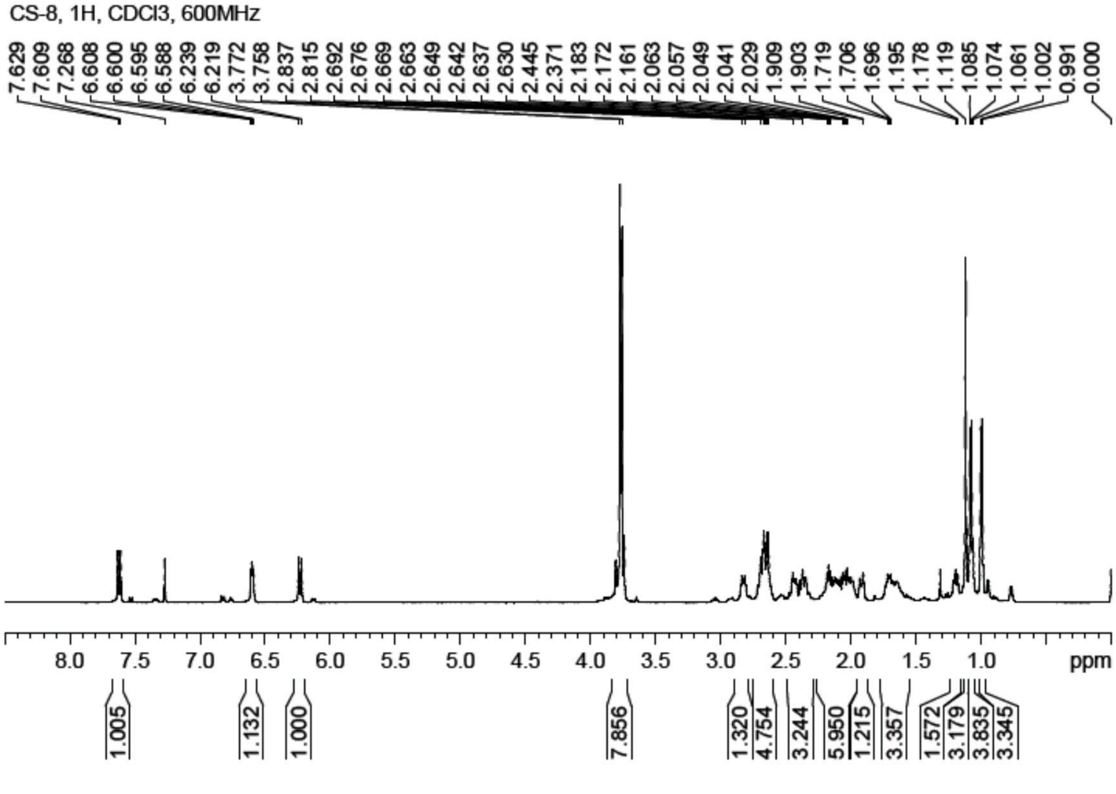

Figure S8. ^{1}H NMR spectrum (600 MHz) of compound **2** in $CDCl_3$.

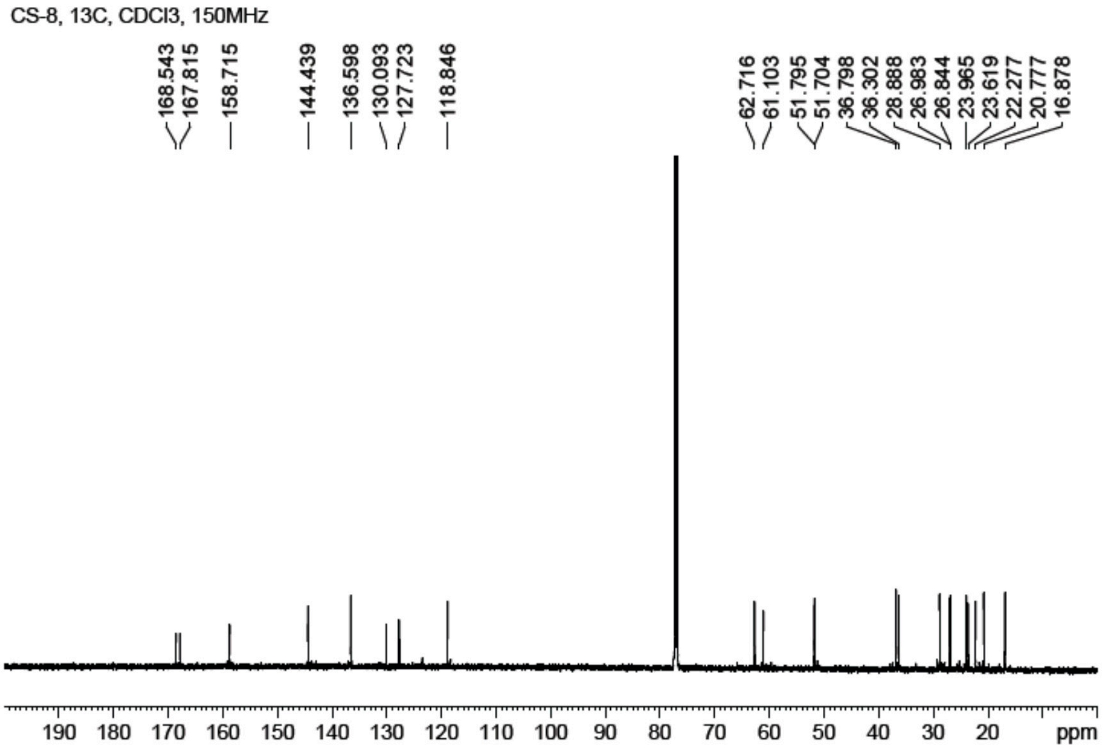

Figure S9. ^{13}C NMR spectrum (150 MHz) of compound **2** in $CDCl_3$.

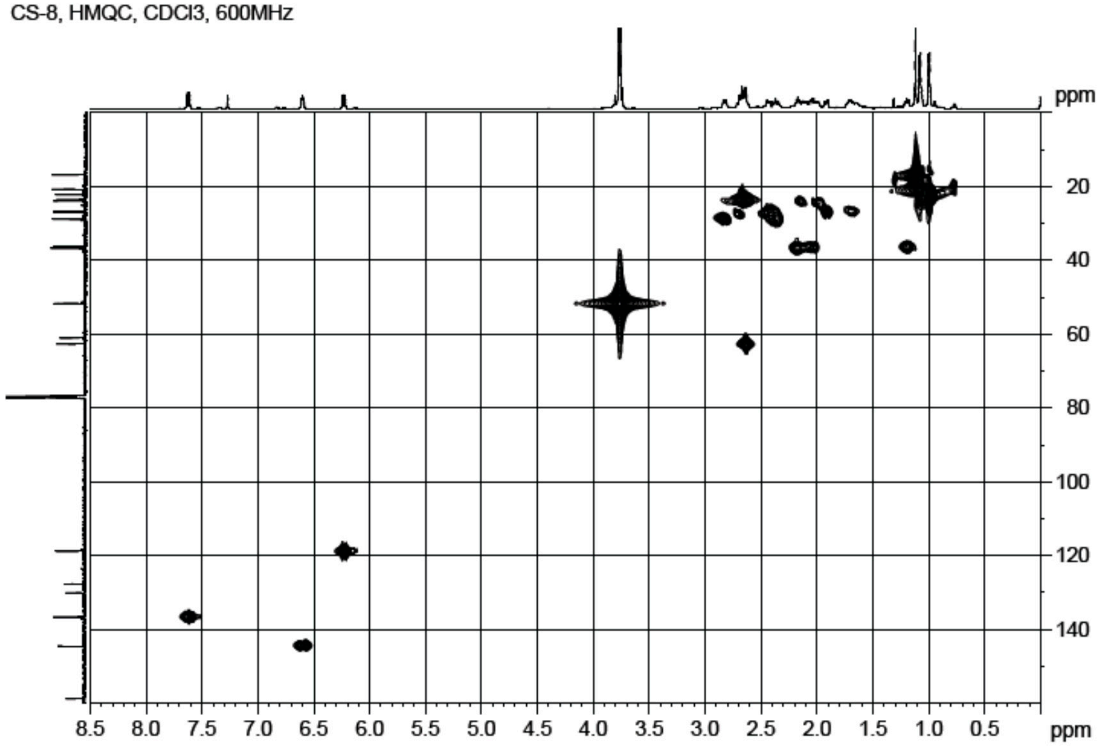

Figure S10. HMQC spectrum of compound **2** in $CDCl_3$.

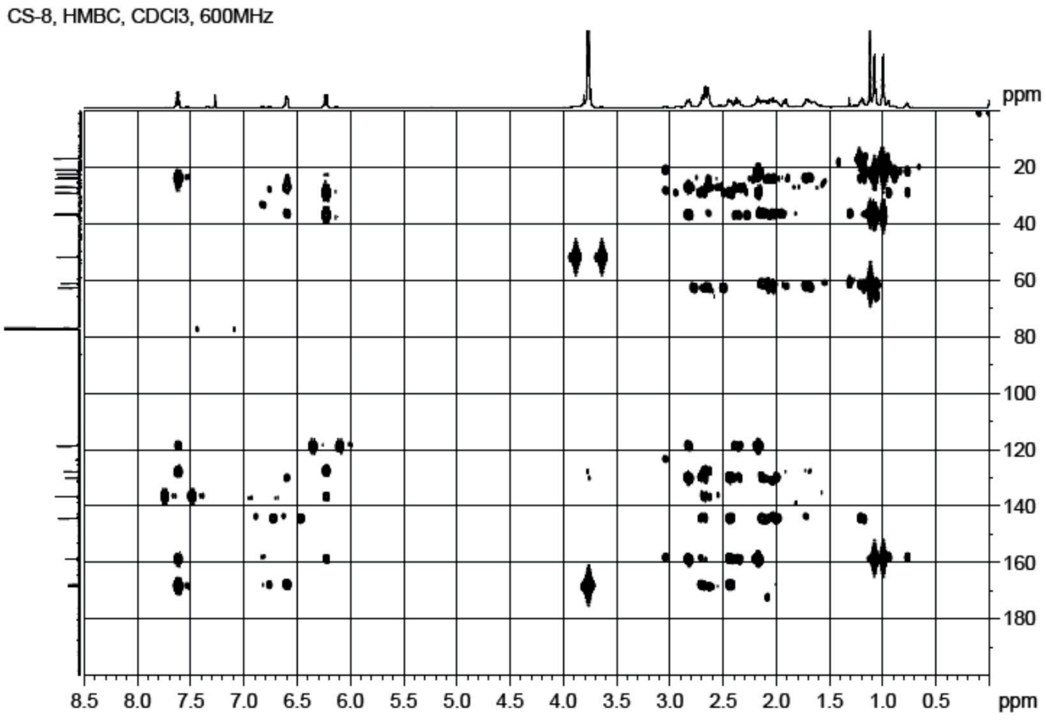

Figure S11. HMBC spectrum of compound **2** in $CDCl_3$.

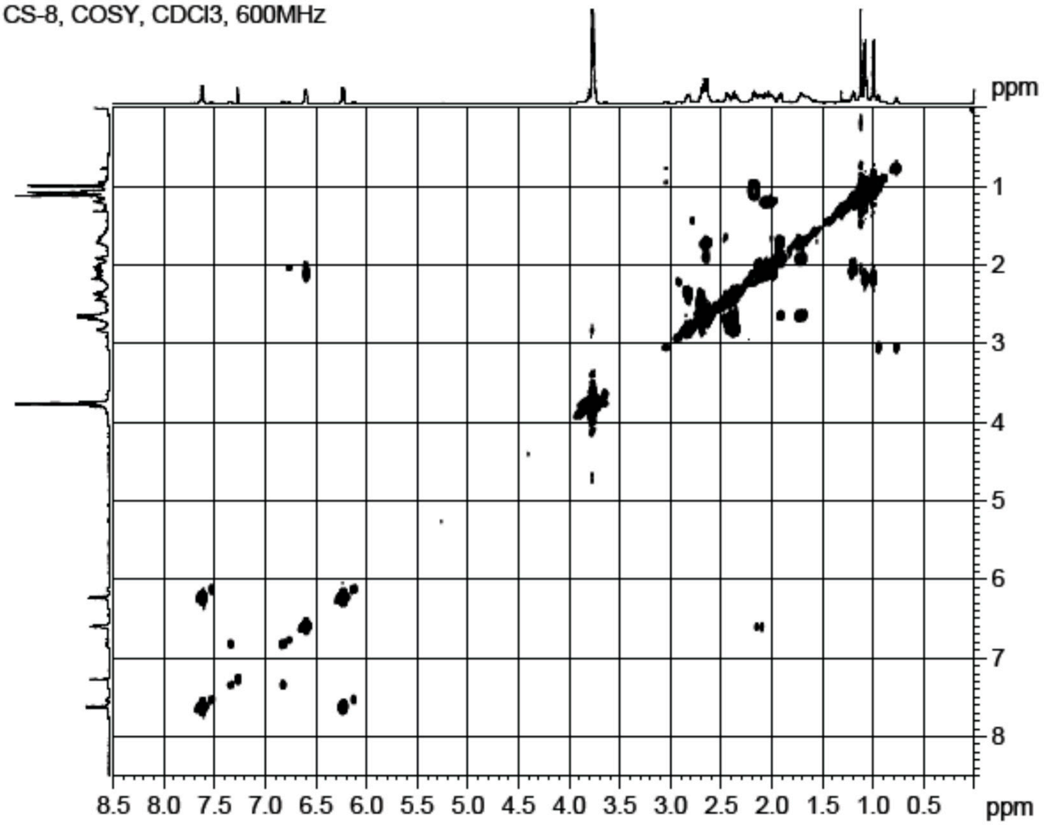

Figure S12. COSY spectrum of compound **2** in $CDCl_3$.

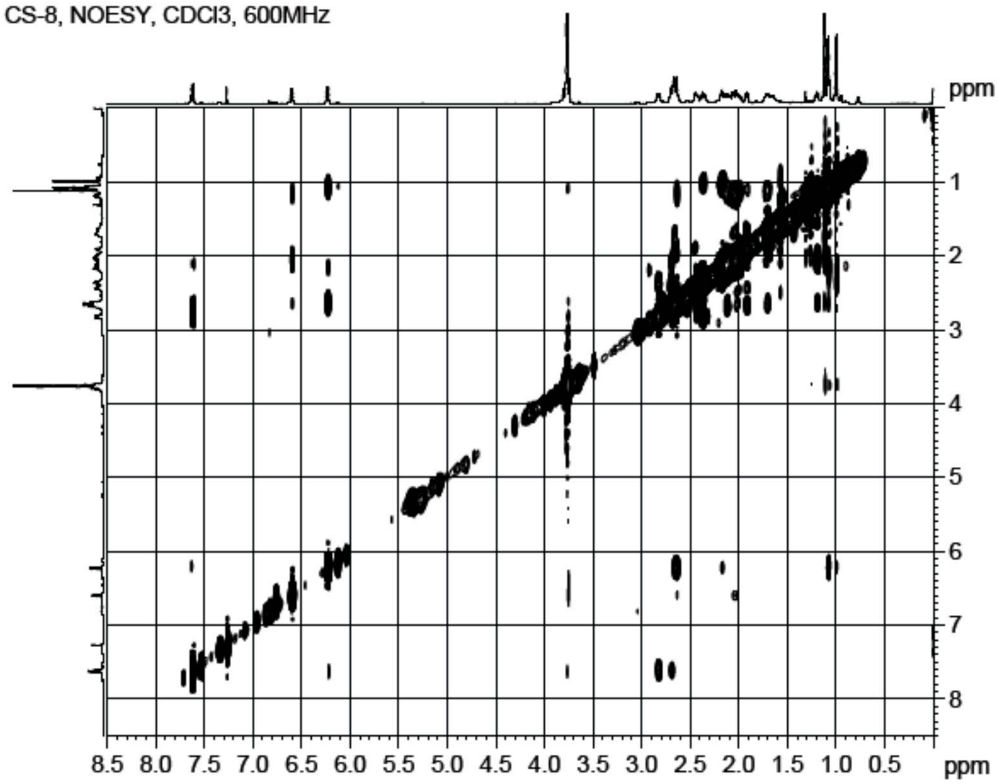

Figure S13. NOESY spectrum of compound **2** in $CDCl_3$.

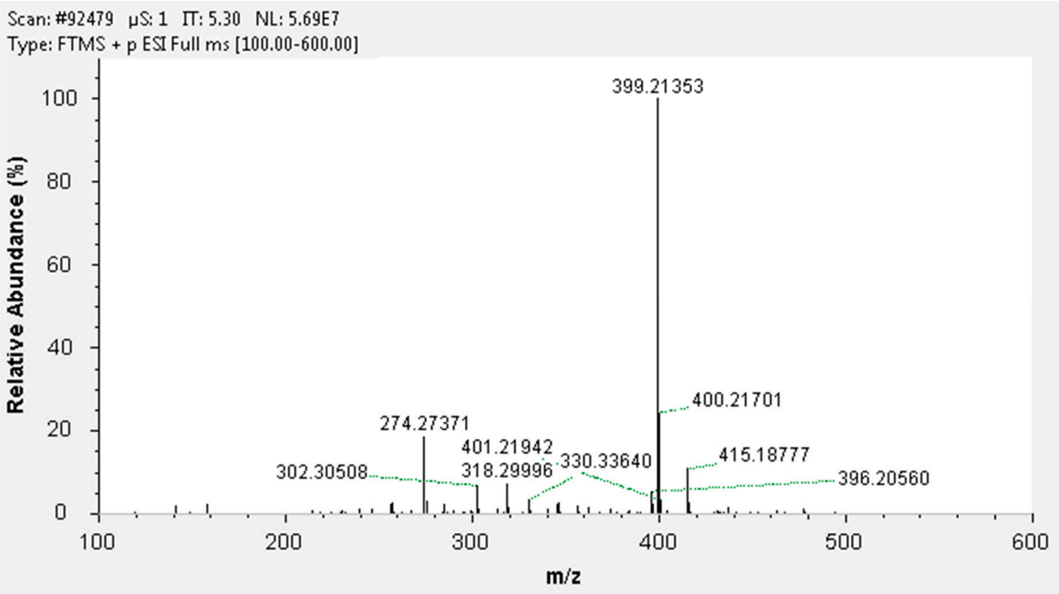

Figure S14. HRESIMS spectrum of compound **2**.

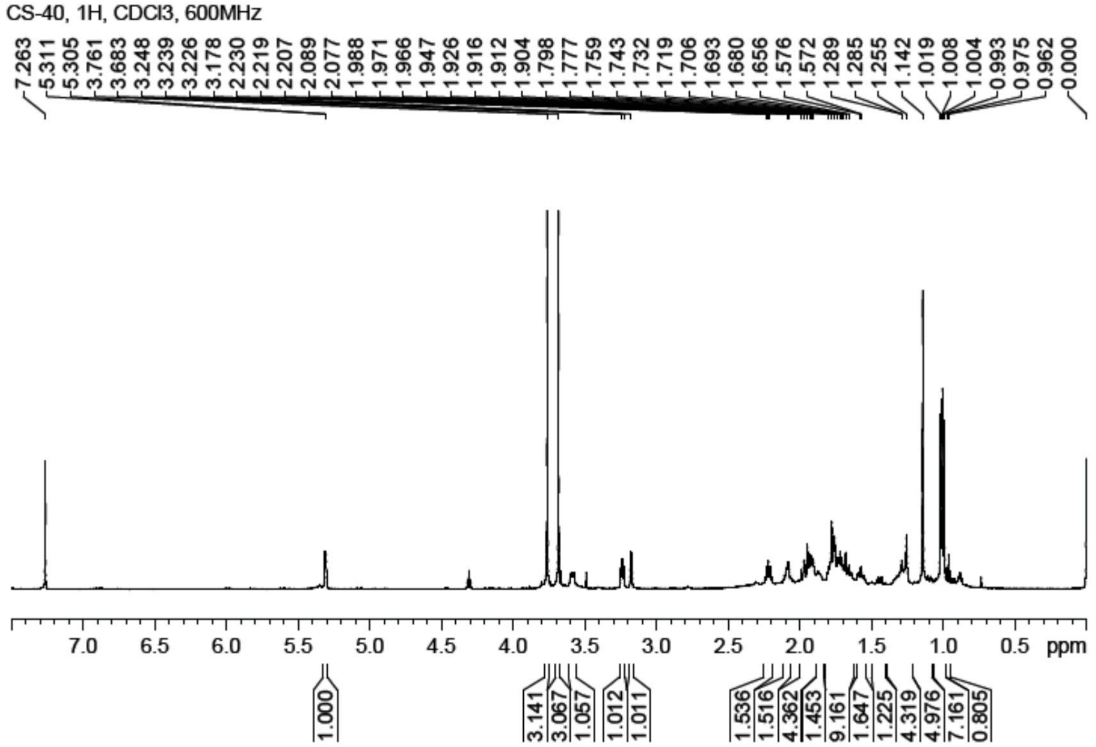

Figure S15. ^{1}H NMR spectrum (600 MHz) of compound **3** in $CDCl_3$.

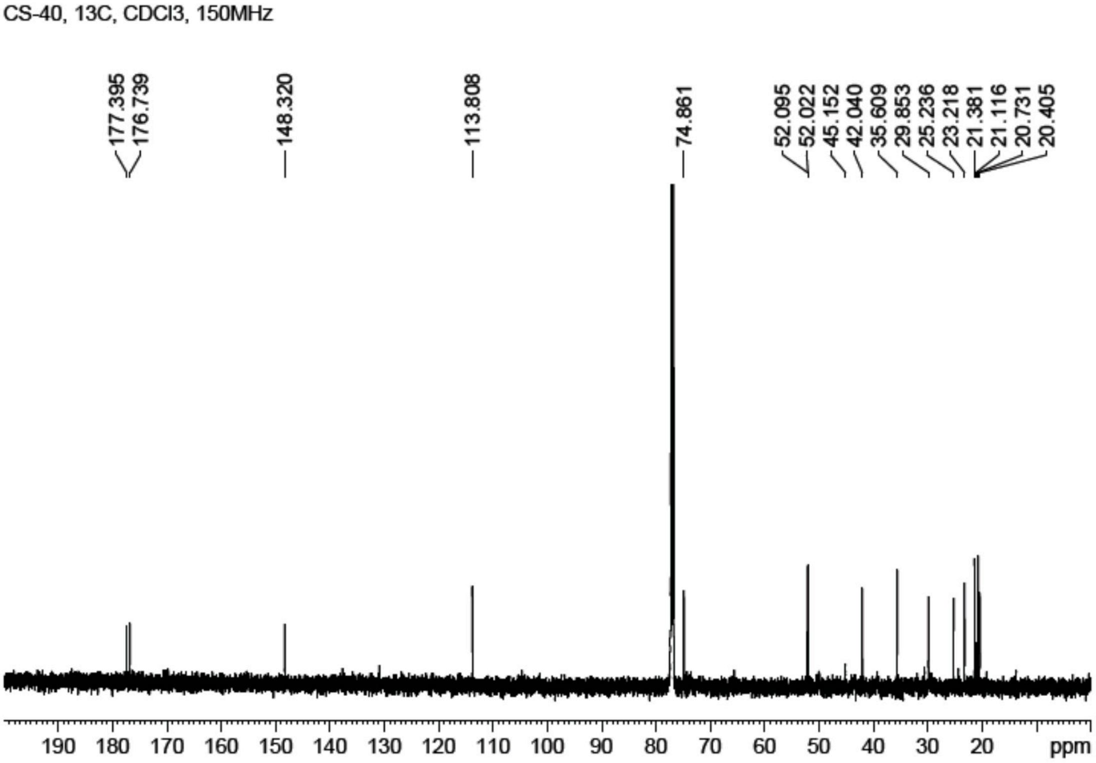

Figure S16. ^{13}C NMR spectrum (150 MHz) of compound **3** in $CDCl_3$.

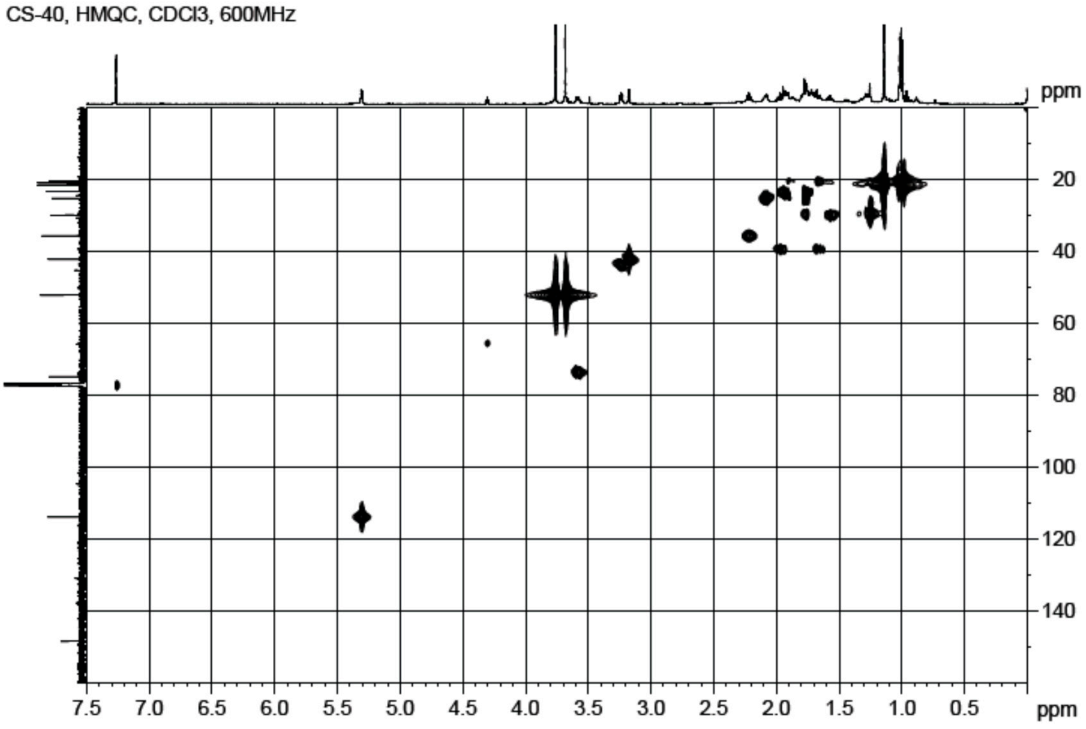

Figure S17. HMQC spectrum of compound **3** in $CDCl_3$.

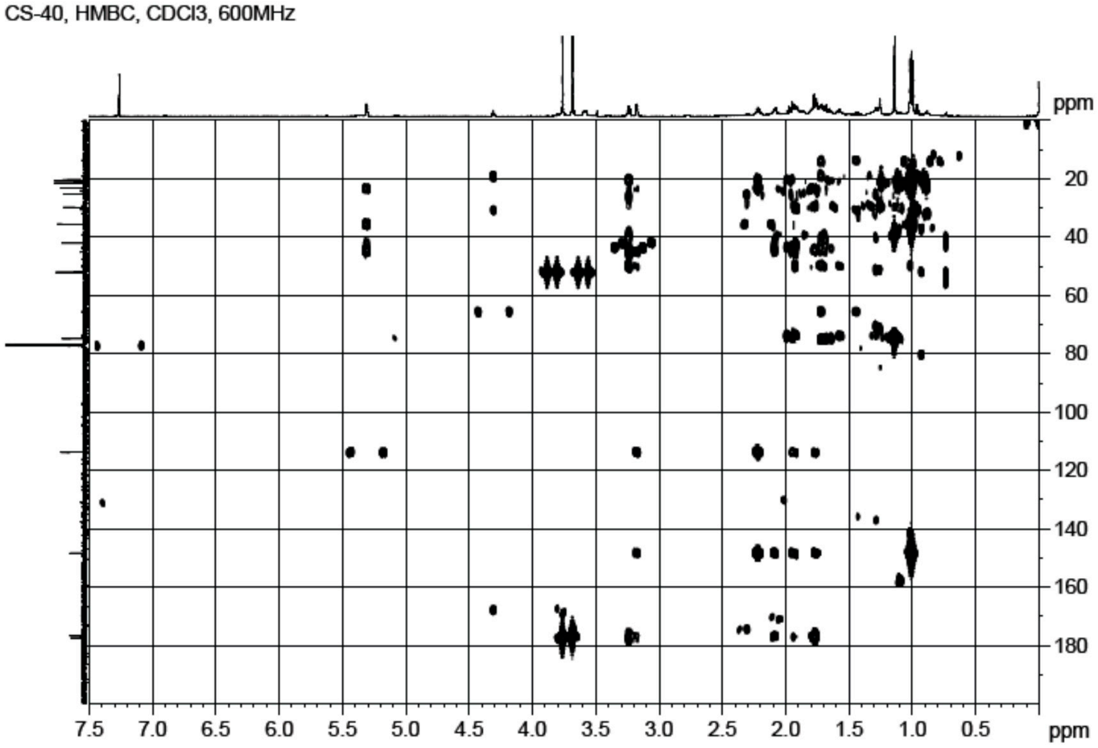

Figure S18. HMBC spectrum of compound **3** in $CDCl_3$.

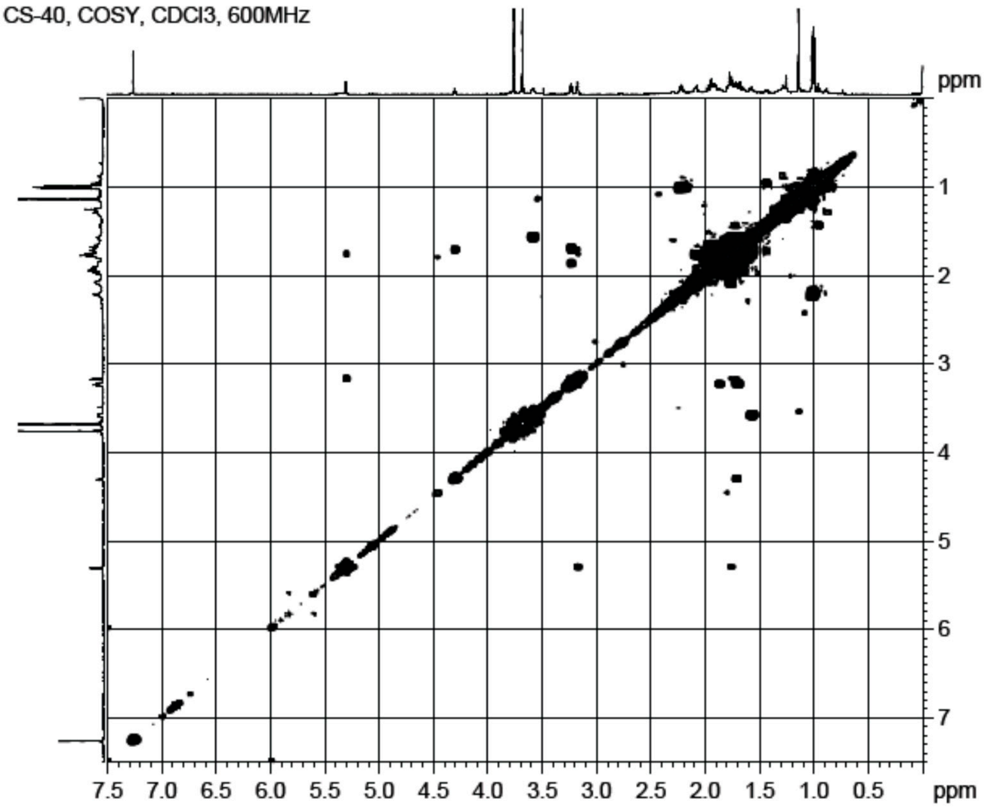

Figure S19. COSY spectrum of compound **3** in $CDCl_3$.

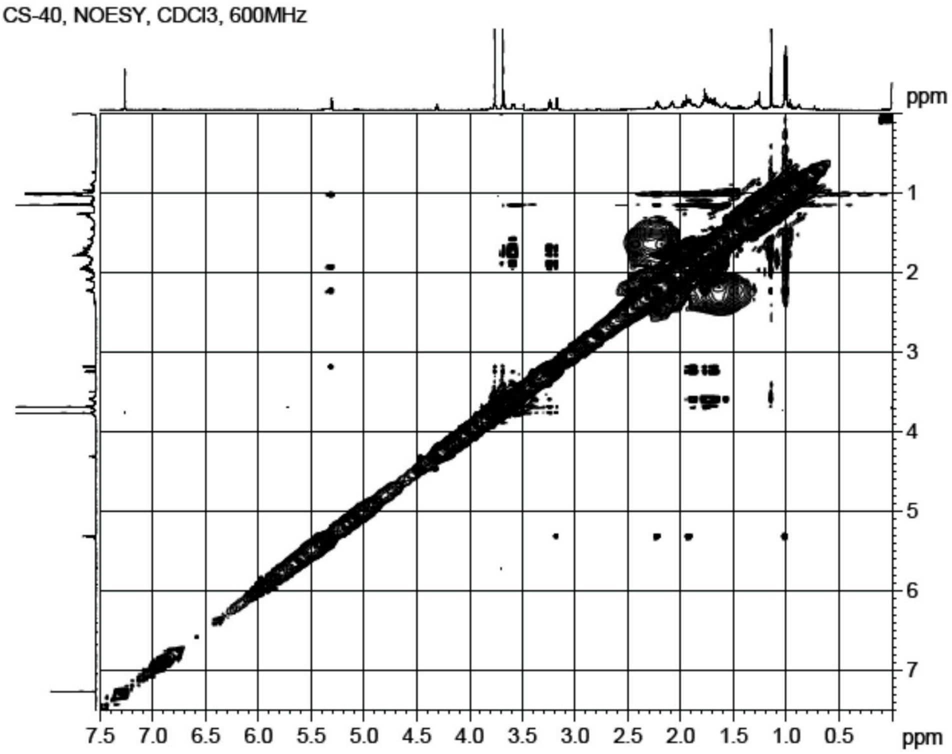

Figure S20. NOESY spectrum of compound **3** in $CDCl_3$.

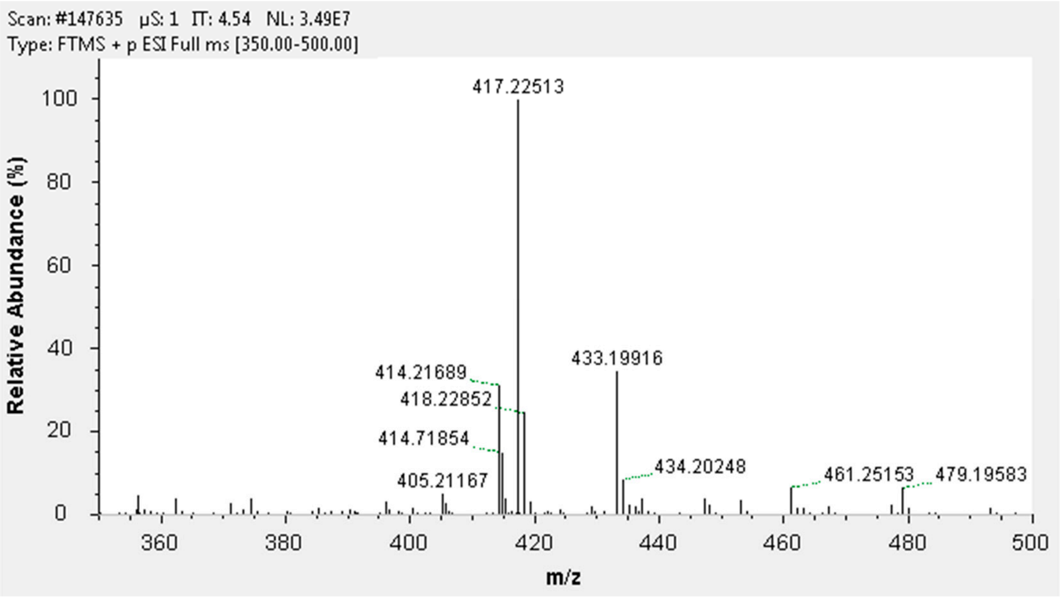

Figure S21. HRESIMS spectrum of compound **3**.

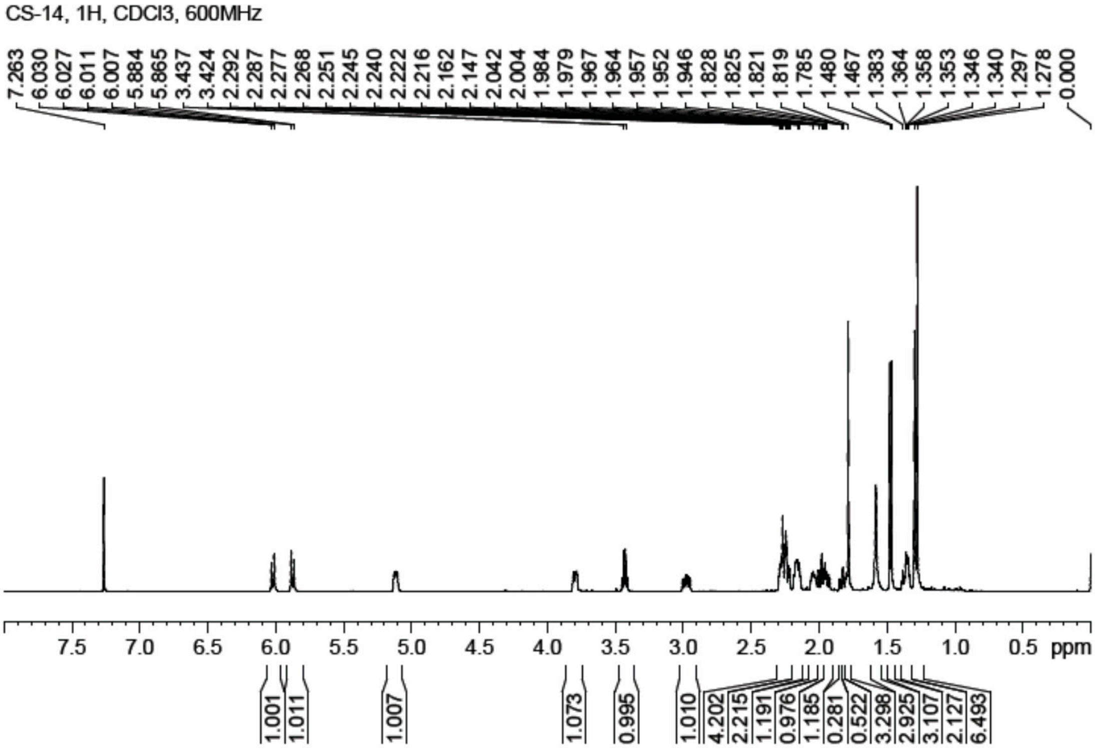

Figure S22. ^{1}H NMR spectrum (600 MHz) of compound **4** in $CDCl_3$.

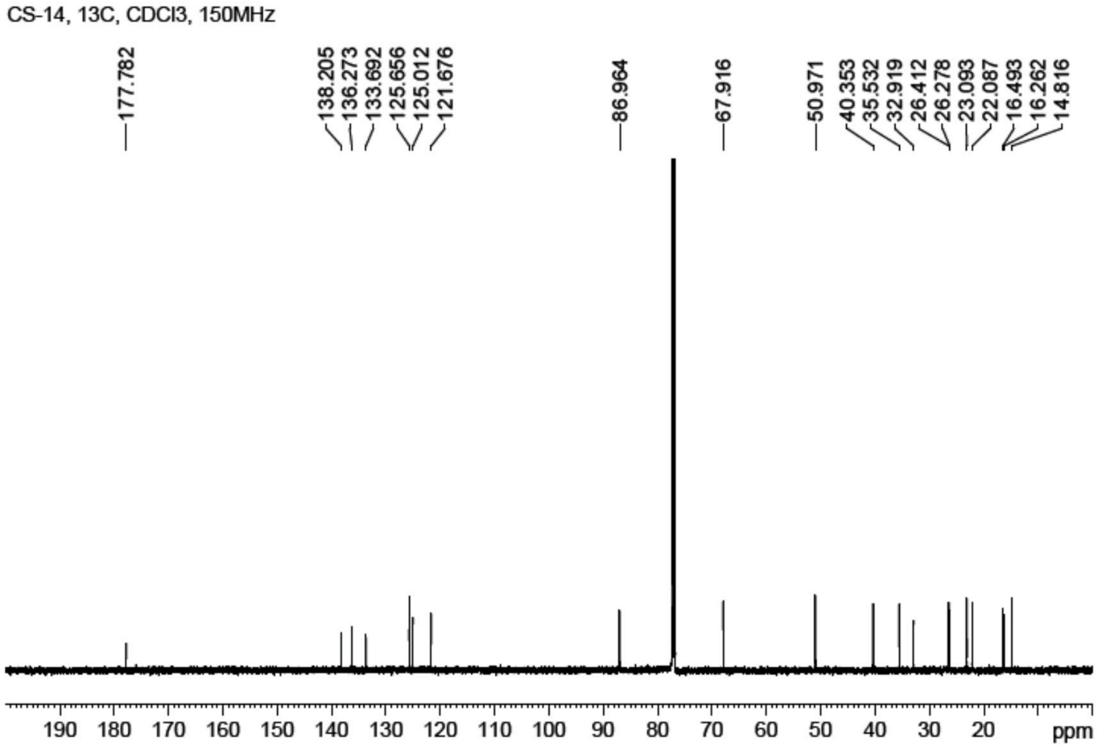

Figure S23. ^{13}C NMR spectrum (150 MHz) of compound **4** in $CDCl_3$.

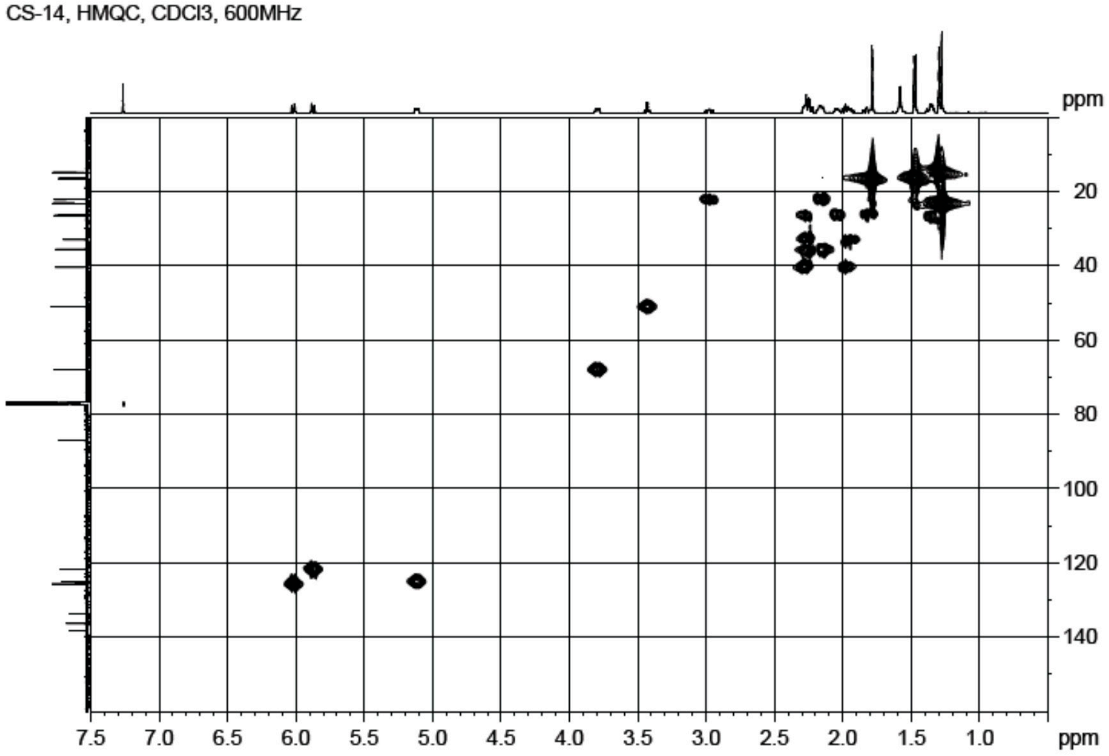

Figure S24. HMQC spectrum of compound **4** in $CDCl_3$.

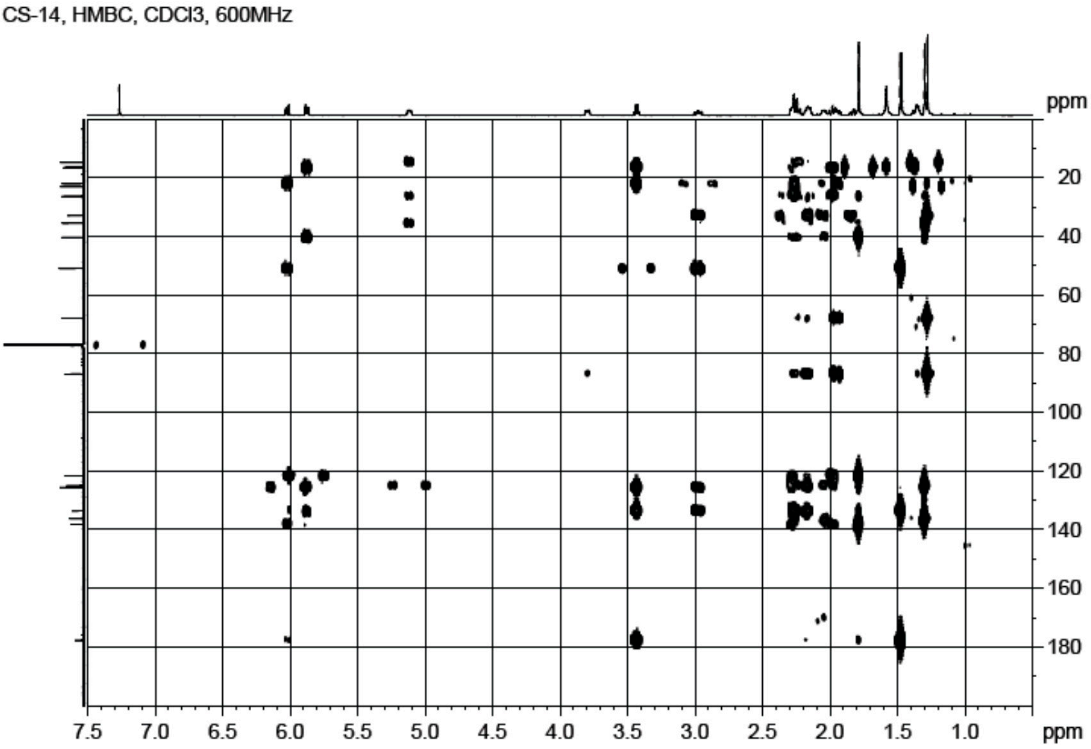

Figure S25. HMBC spectrum of compound **4** in $CDCl_3$.

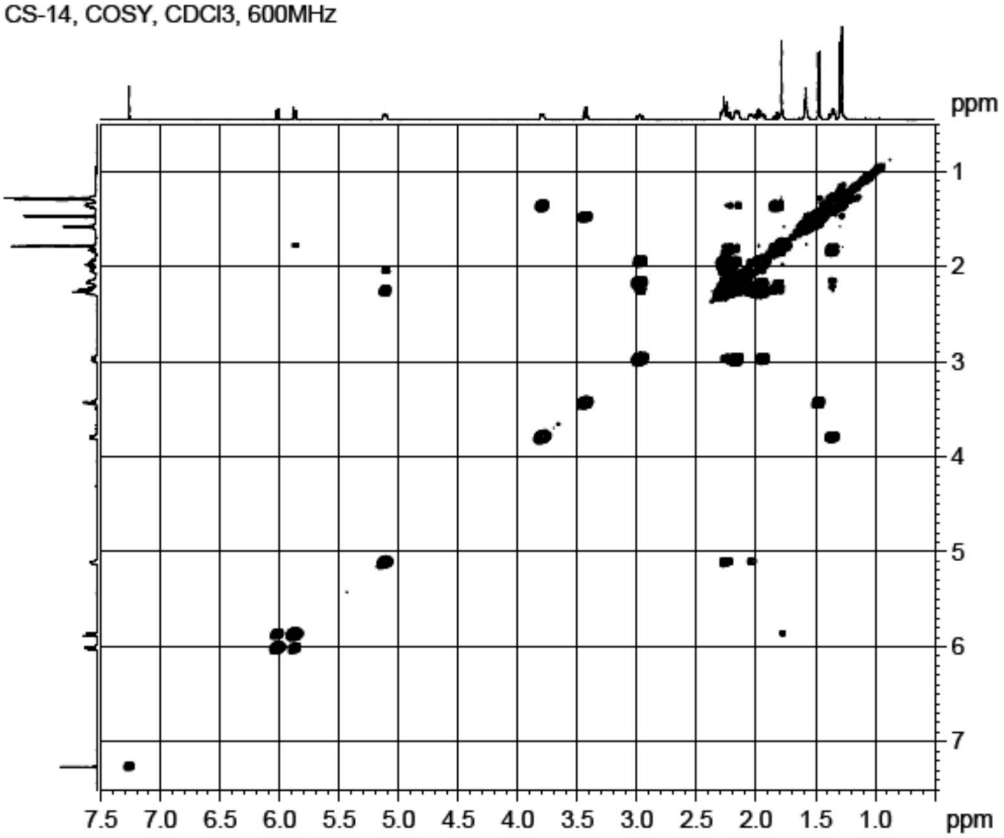

Figure S26. COSY spectrum of compound **4** in $CDCl_3$.

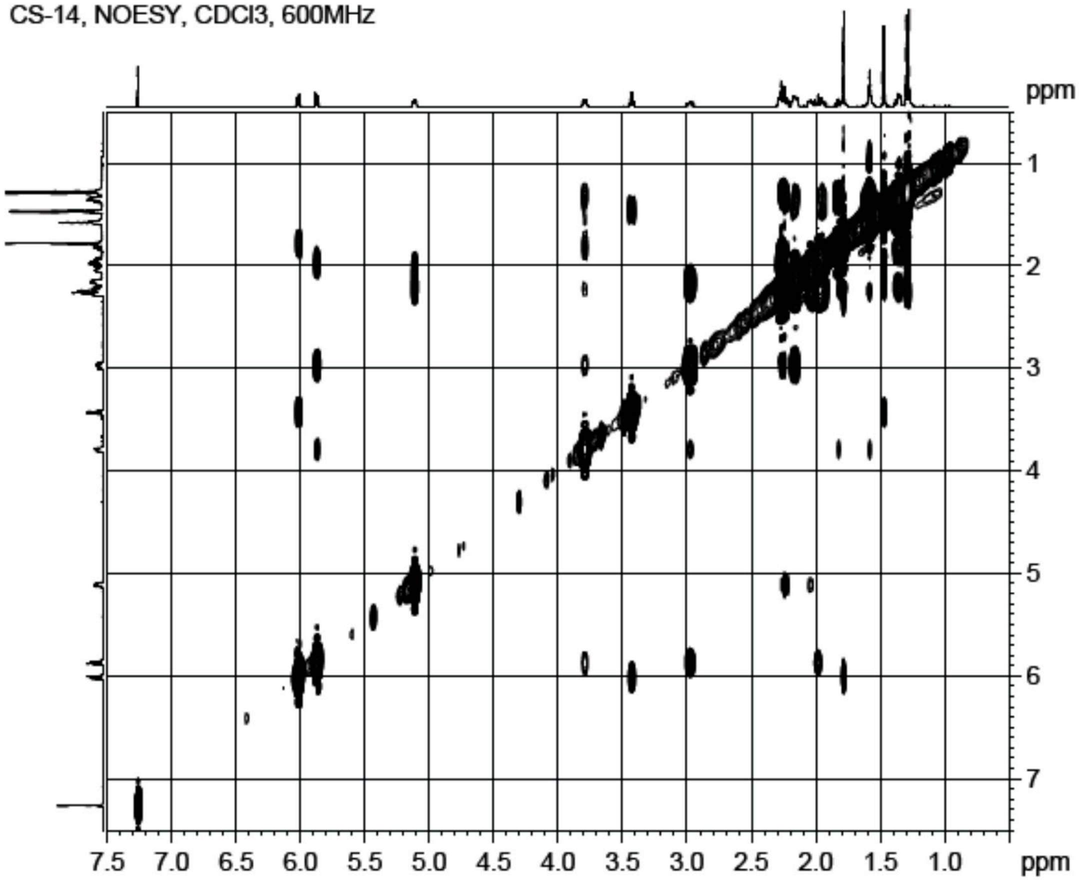

Figure S27. NOSY spectrum of compound **4** in $CDCl_3$.

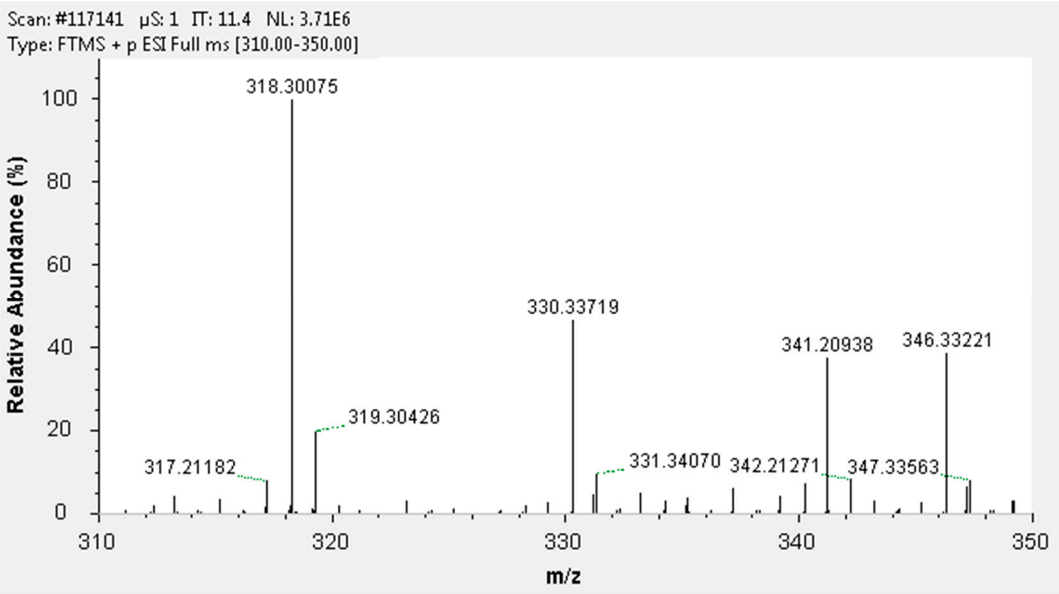

Figure S28. HRESIMS spectrum of compound **4**.

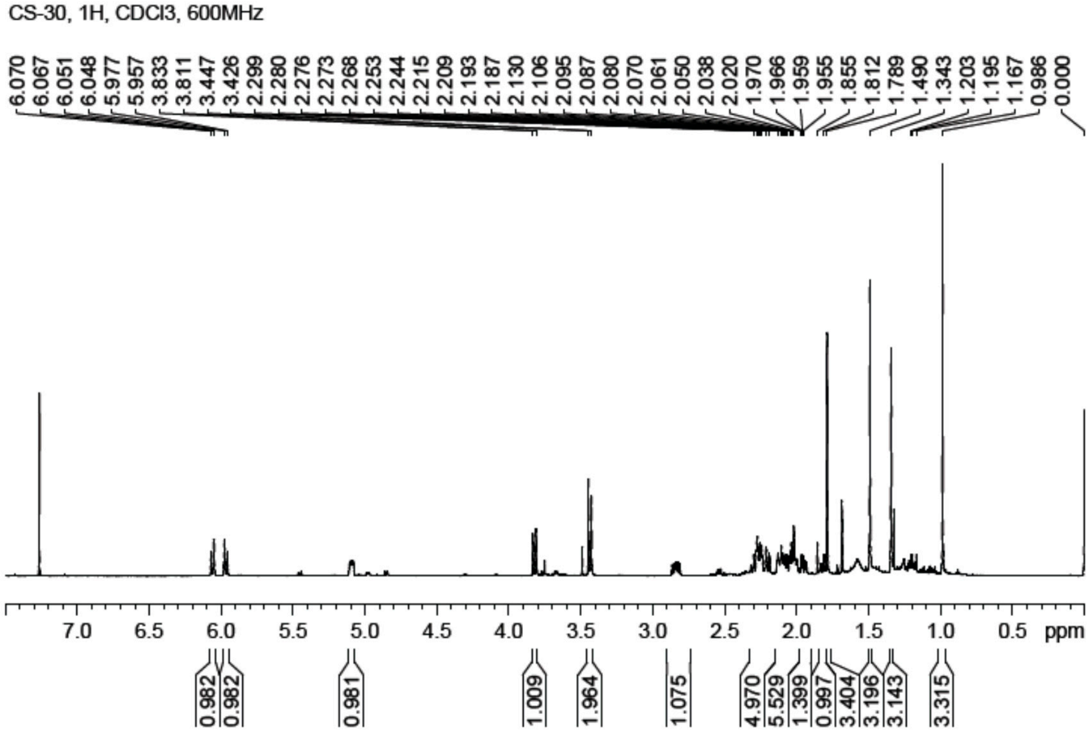

Figure S29. ^{1}H NMR spectrum (600 MHz) of compound **5** in $CDCl_3$.

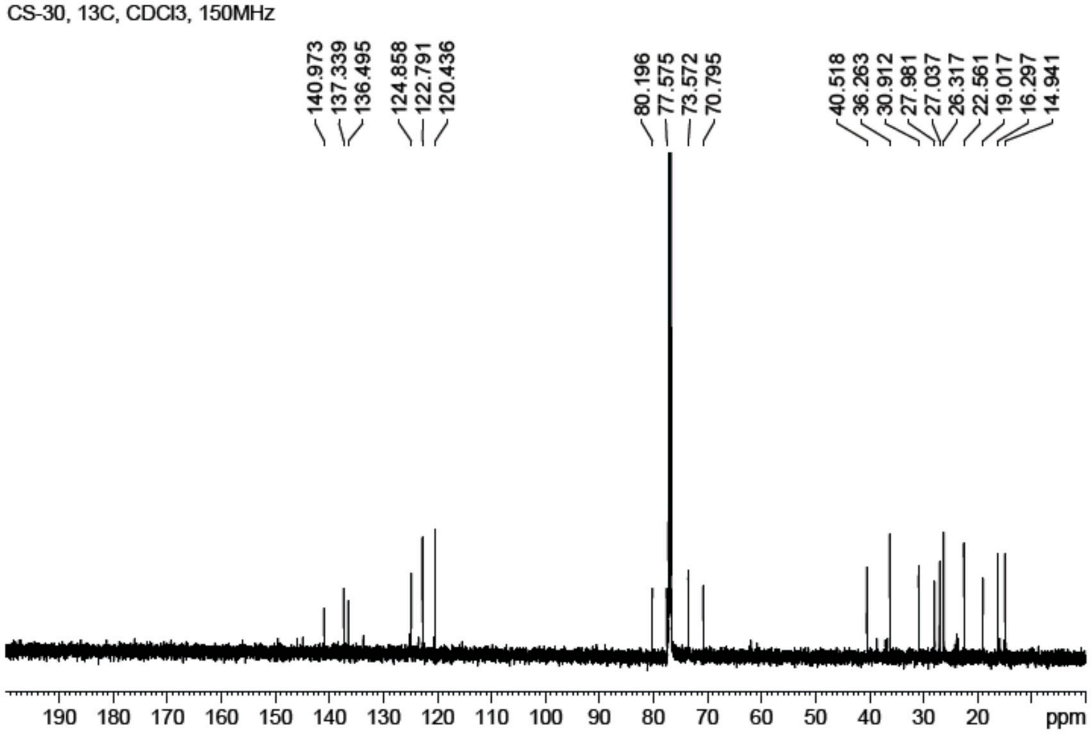

Figure S30. ^{13}C NMR spectrum (150 MHz) of compound **5** in $CDCl_3$.

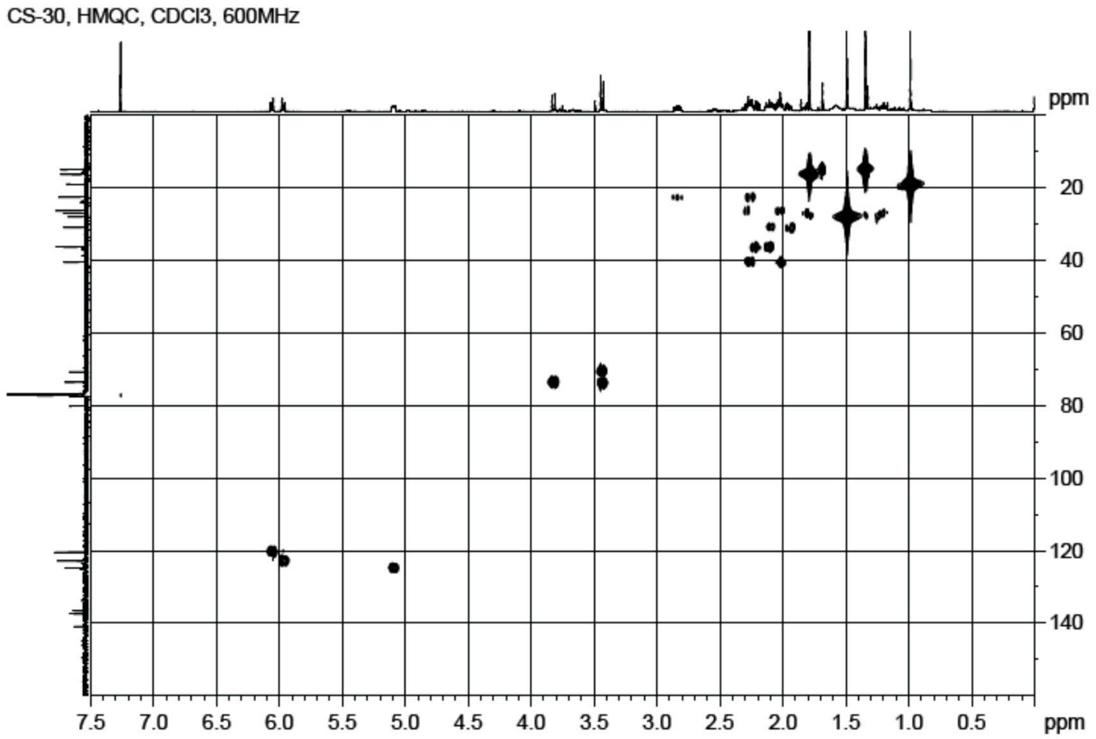

Figure S31. HMQC spectrum of compound **5** in $CDCl_3$.

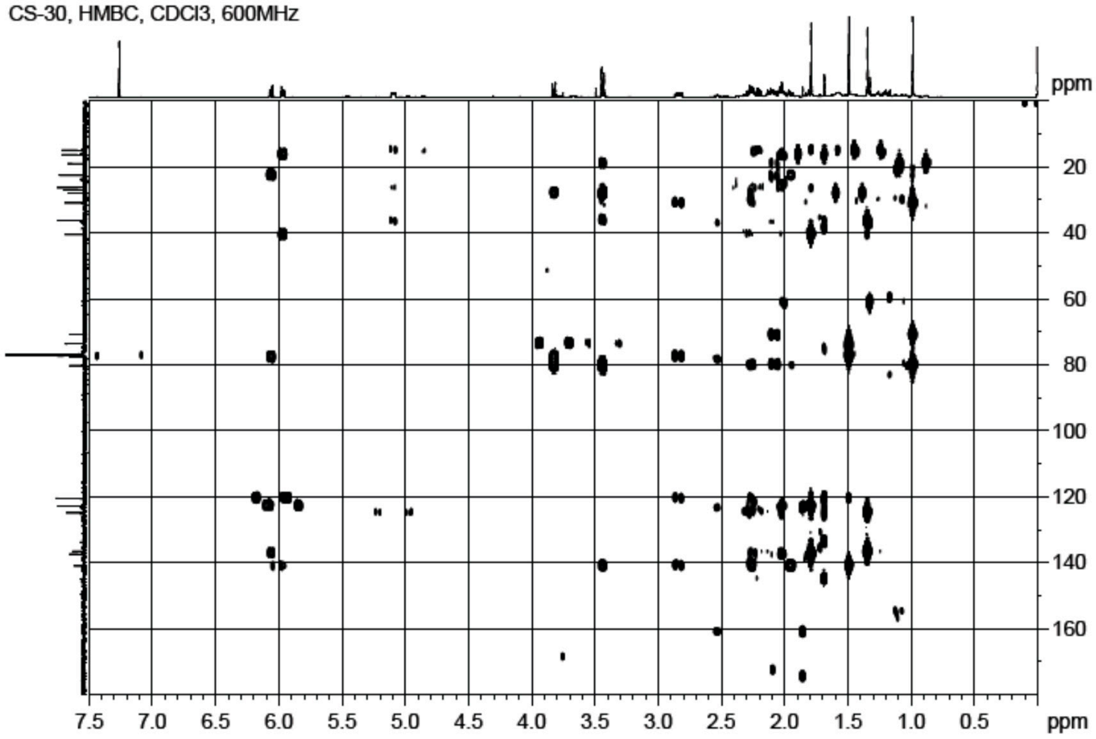

Figure S32. HMBC spectrum of compound **5** in $CDCl_3$.

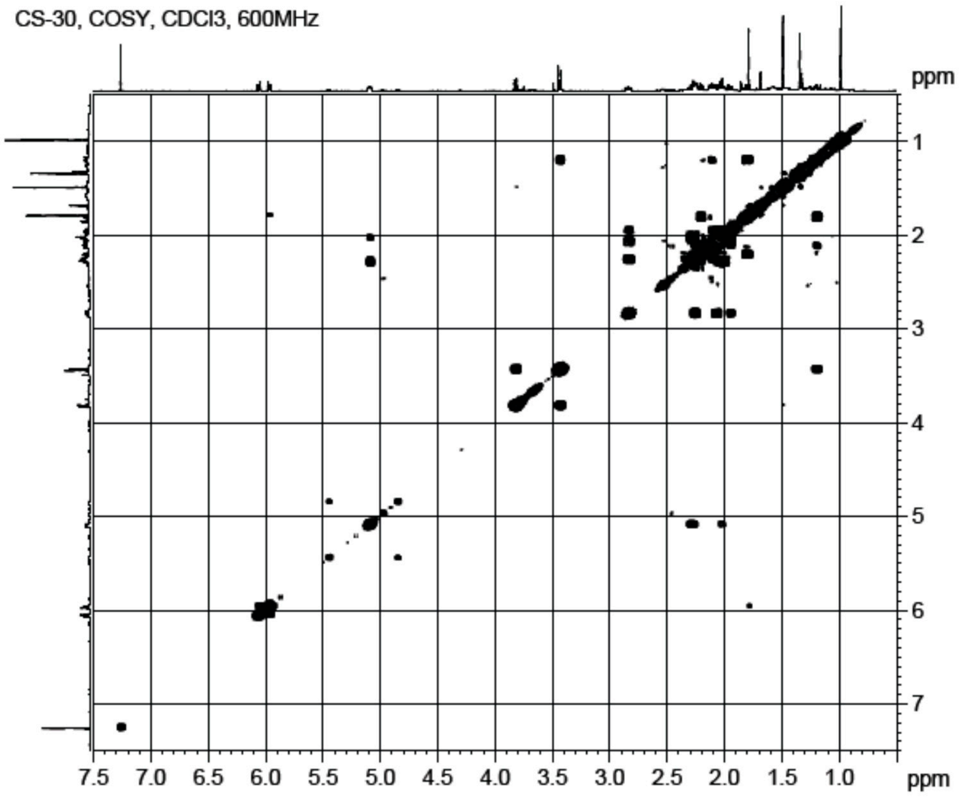

Figure S33. COSY spectrum of compound **5** in $CDCl_3$.

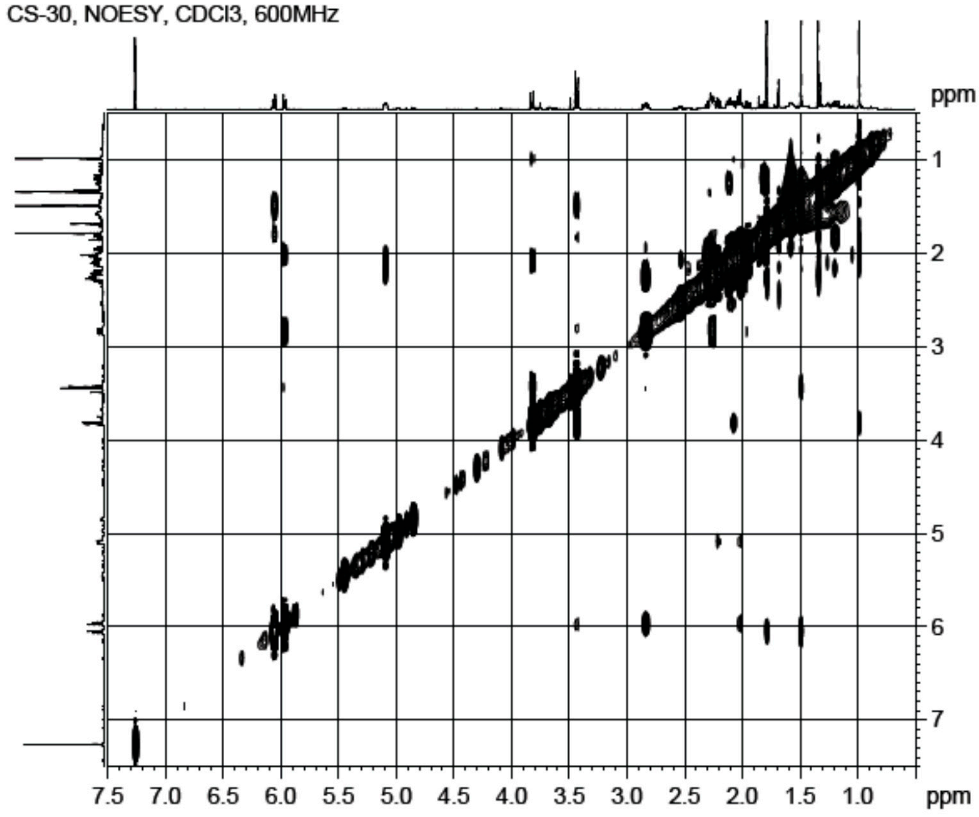

Figure S34. NOESY spectrum of compound **5** in $CDCl_3$.

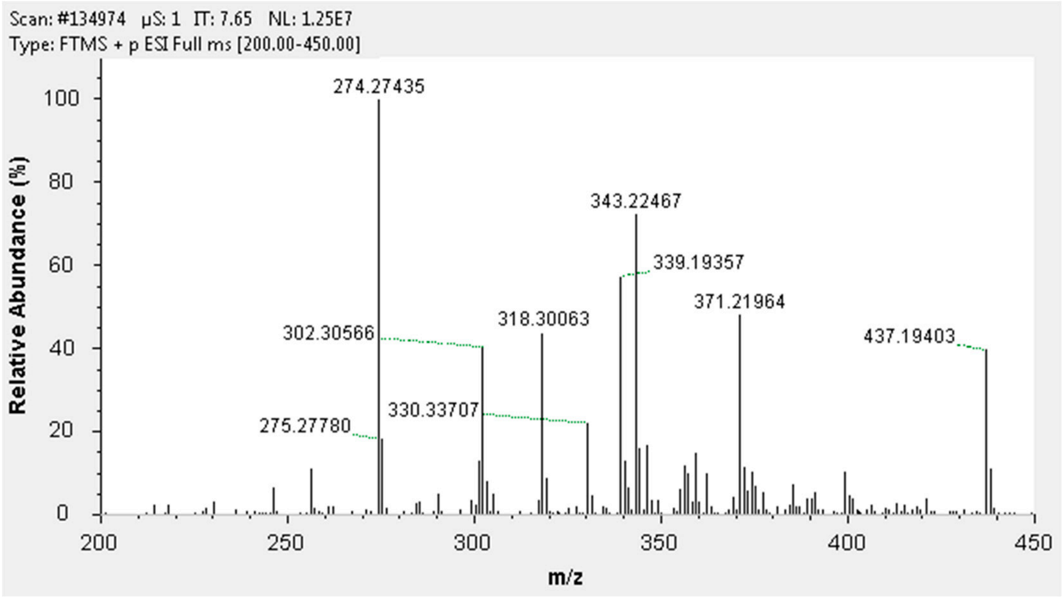

Figure S35. HRESIMS spectrum of compound **5**.

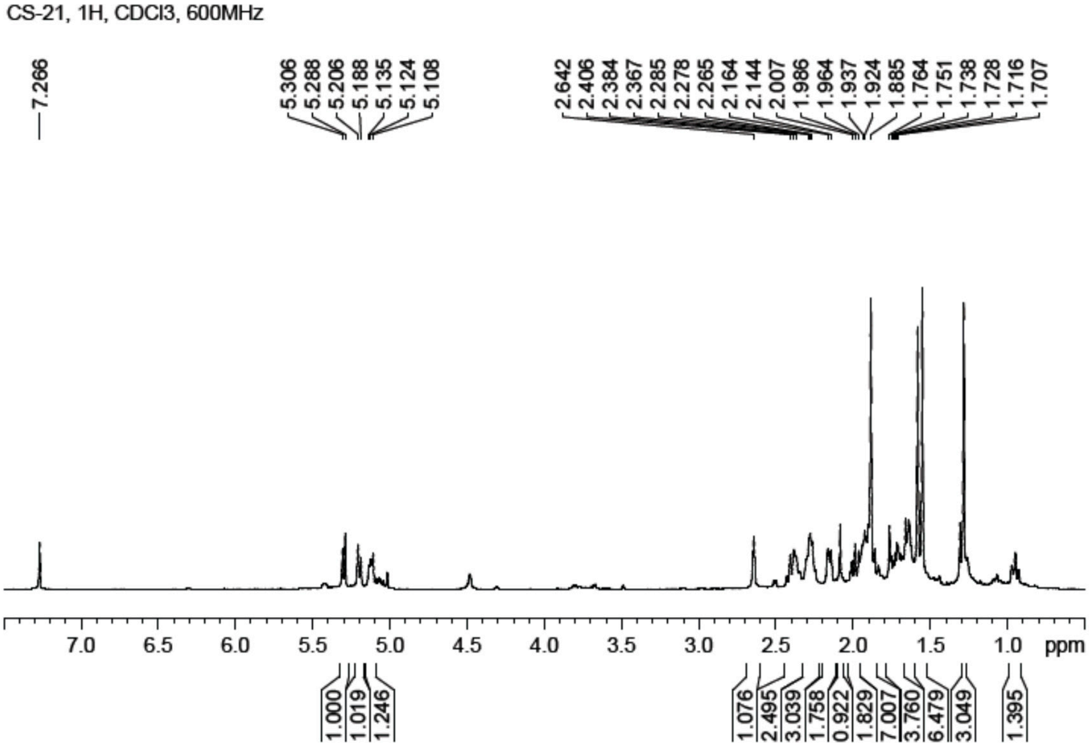

Figure S36. ^{1}H NMR spectrum (600 MHz) of compound **6** in $CDCl_3$.

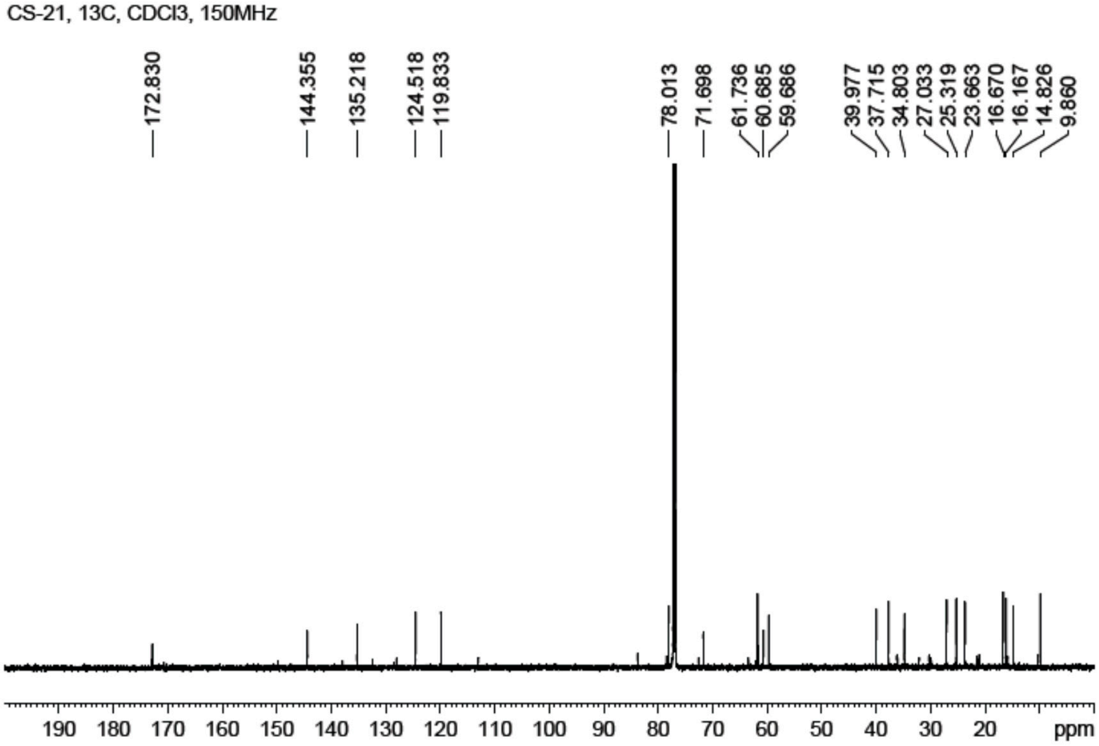

Figure S37. ^{13}C NMR spectrum (150 MHz) of compound **6** in $CDCl_3$.

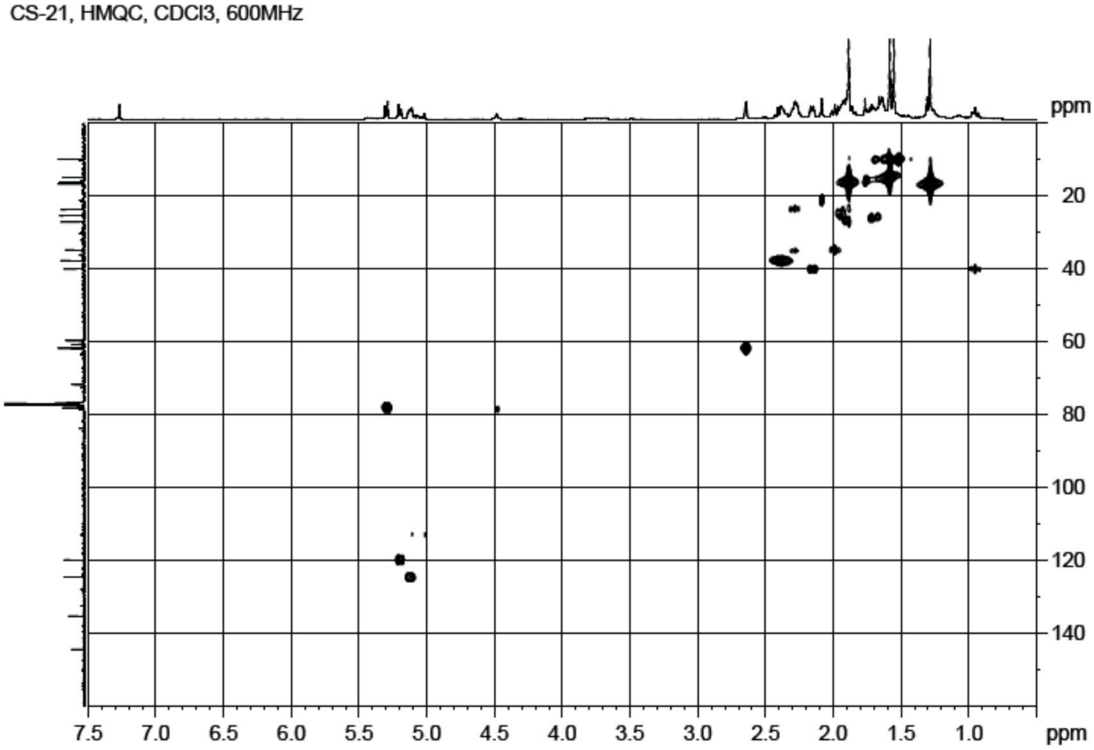

Figure S38. HMQC spectrum of compound **6** in $CDCl_3$.

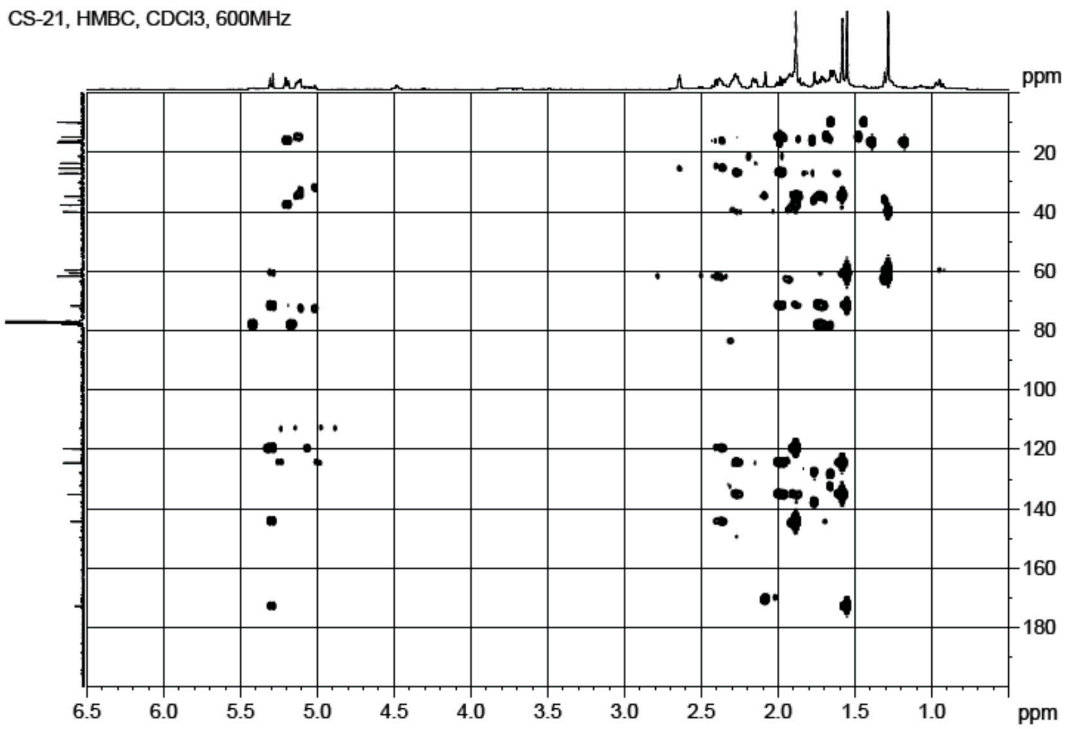

Figure S39. HMBC spectrum of compound **6** in $CDCl_3$.

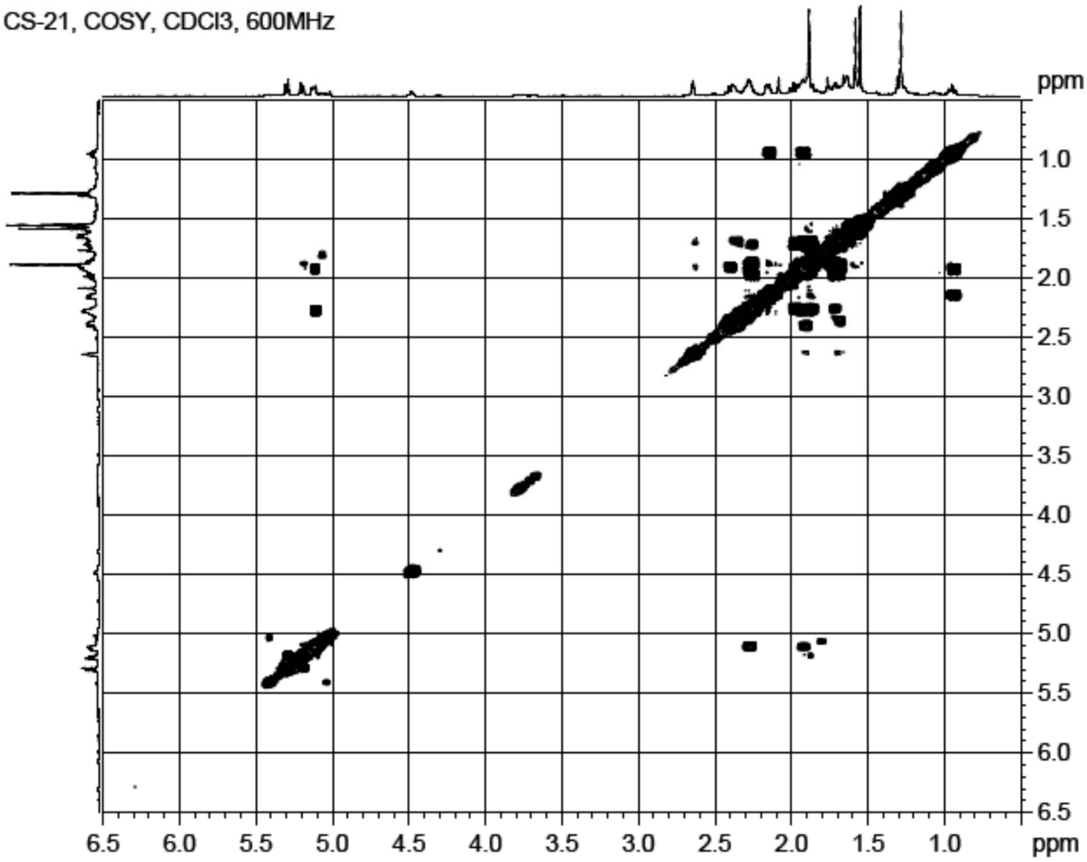

Figure S40. COSY spectrum of compound **6** in $CDCl_3$.

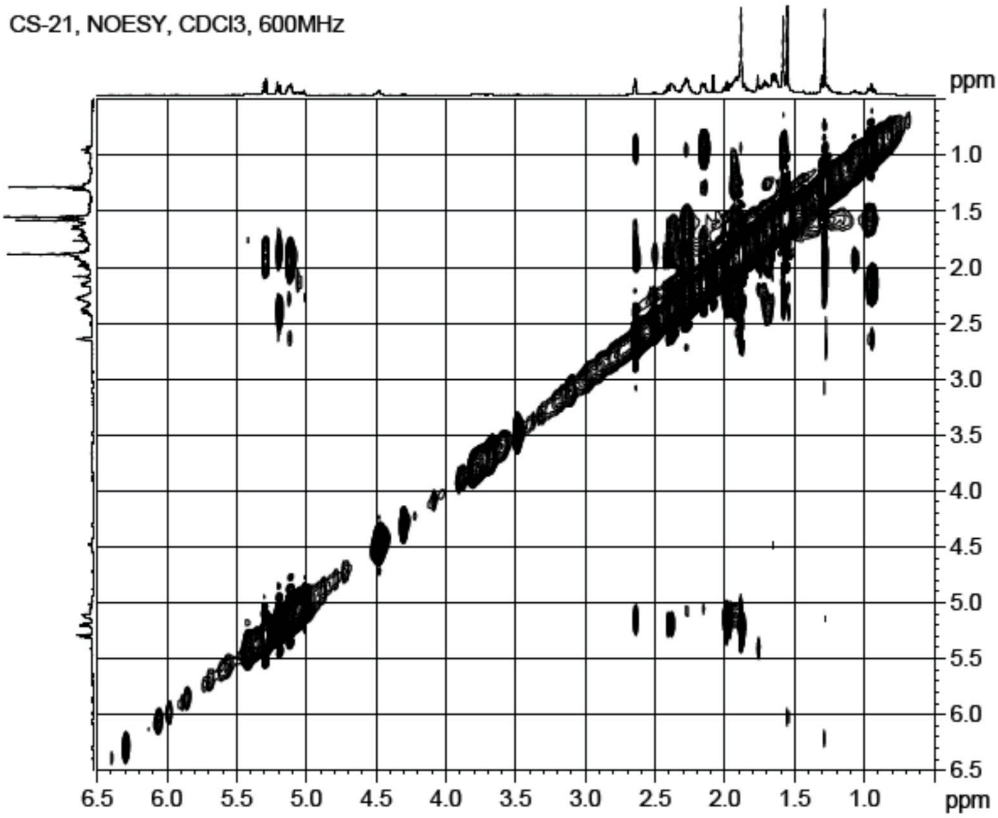

Figure S41. NOESY spectrum of compound **6** in $CDCl_3$.

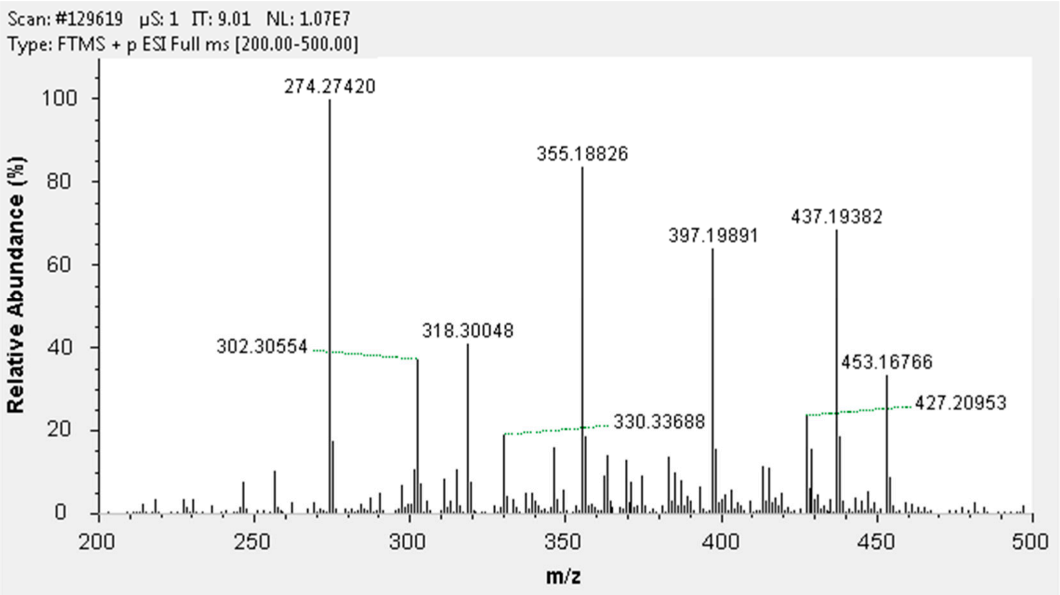

Figure S42. HRESIMS spectrum of compound **6**.

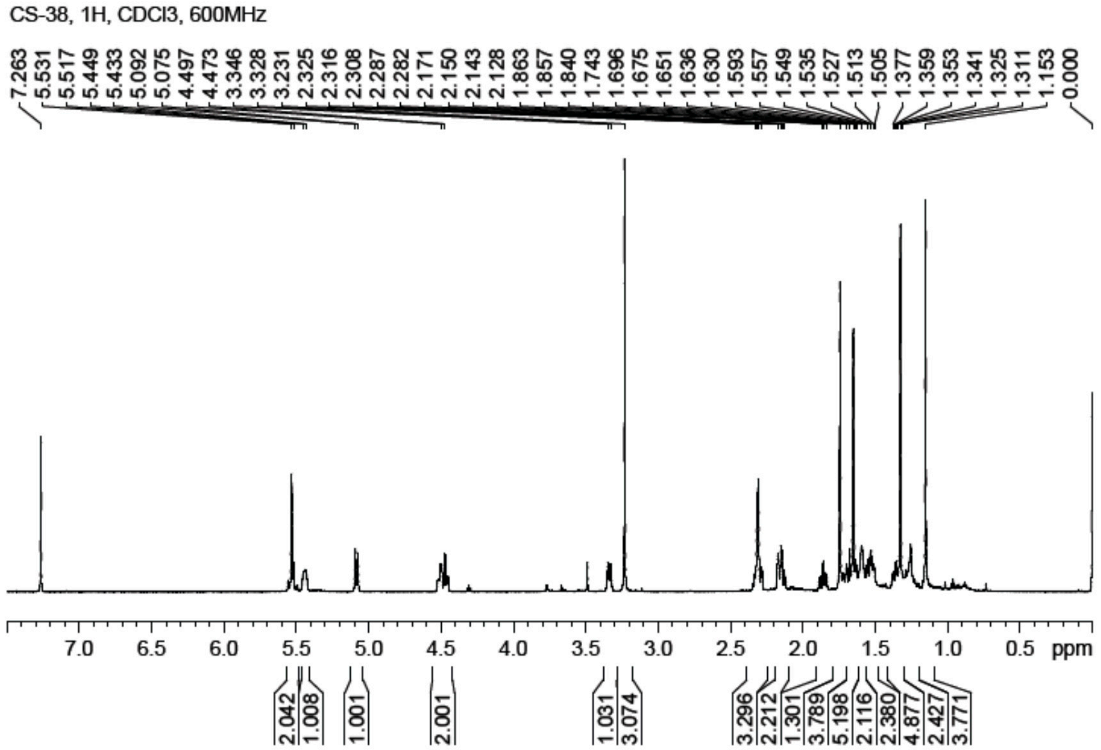

Figure S43. ^{1}H NMR spectrum (600 MHz) of compound **7** in $CDCl_3$.

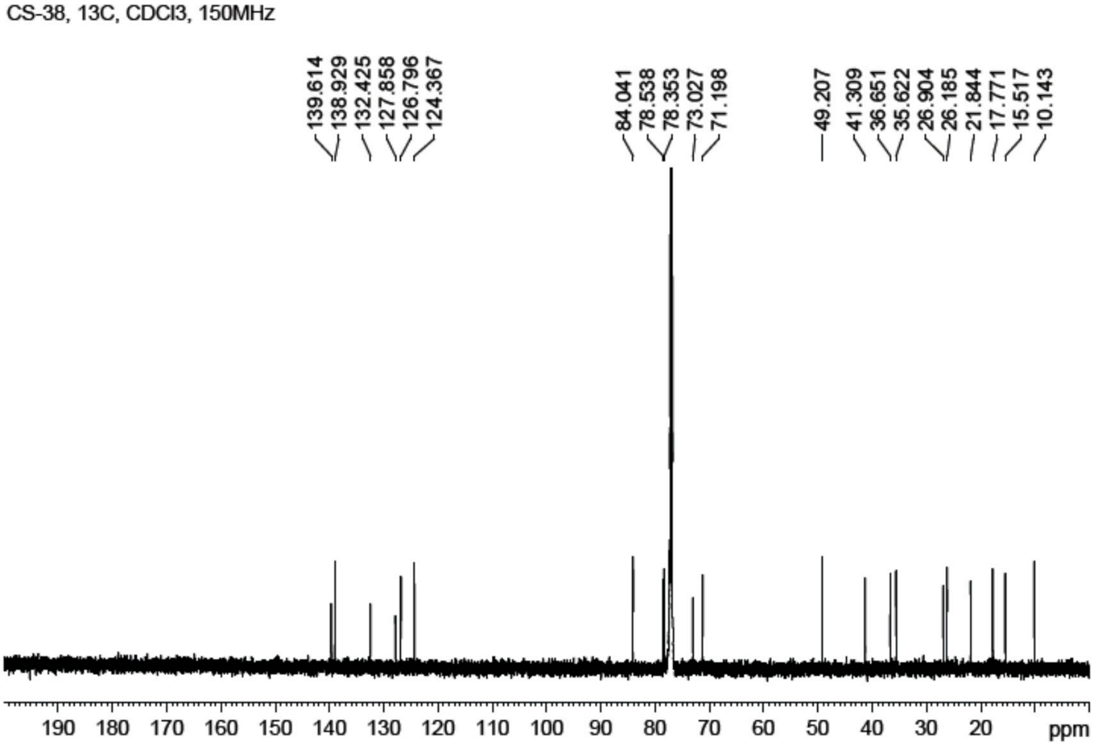

Figure S44. ^{13}C NMR spectrum (150 MHz) of compound **7** in $CDCl_3$.

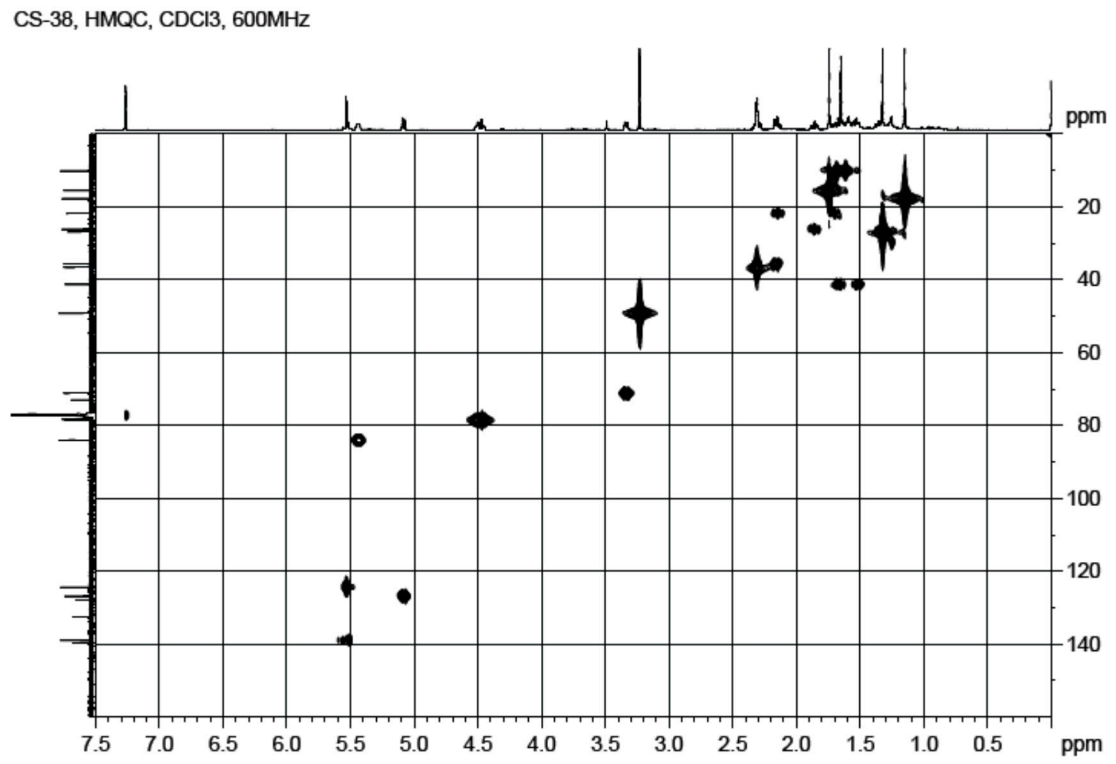

Figure S45. HMQC spectrum of compound **7** in $CDCl_3$.

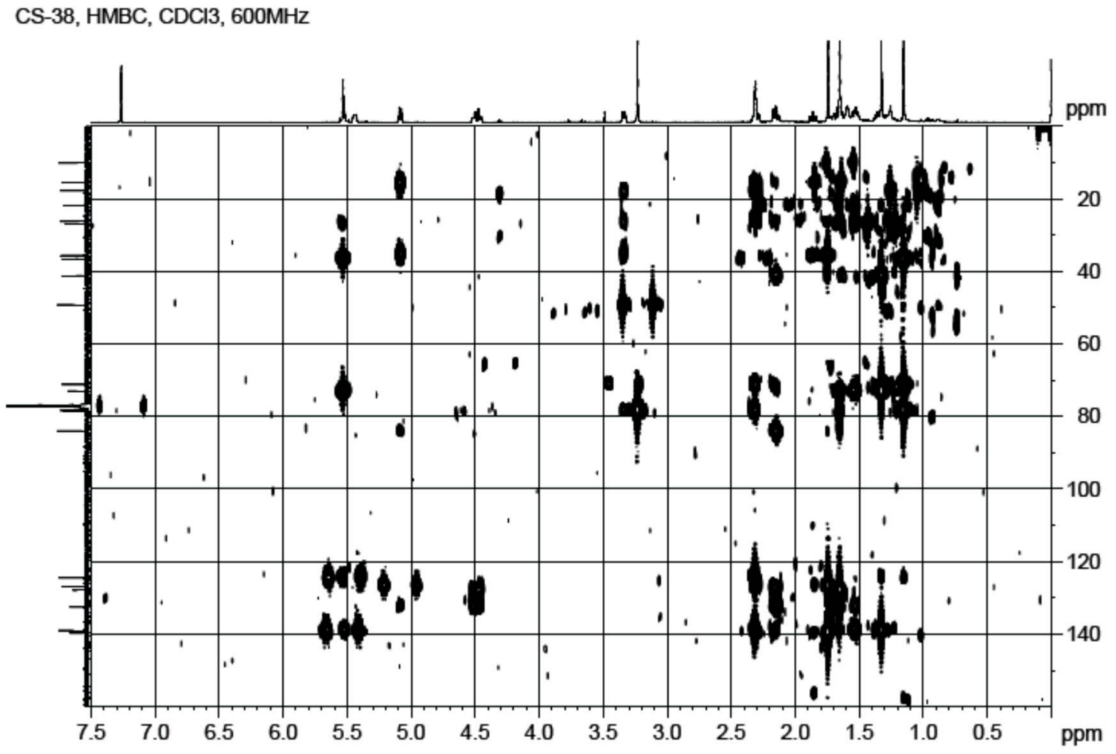

Figure S46. HMBC spectrum of compound **7** in $CDCl_3$.

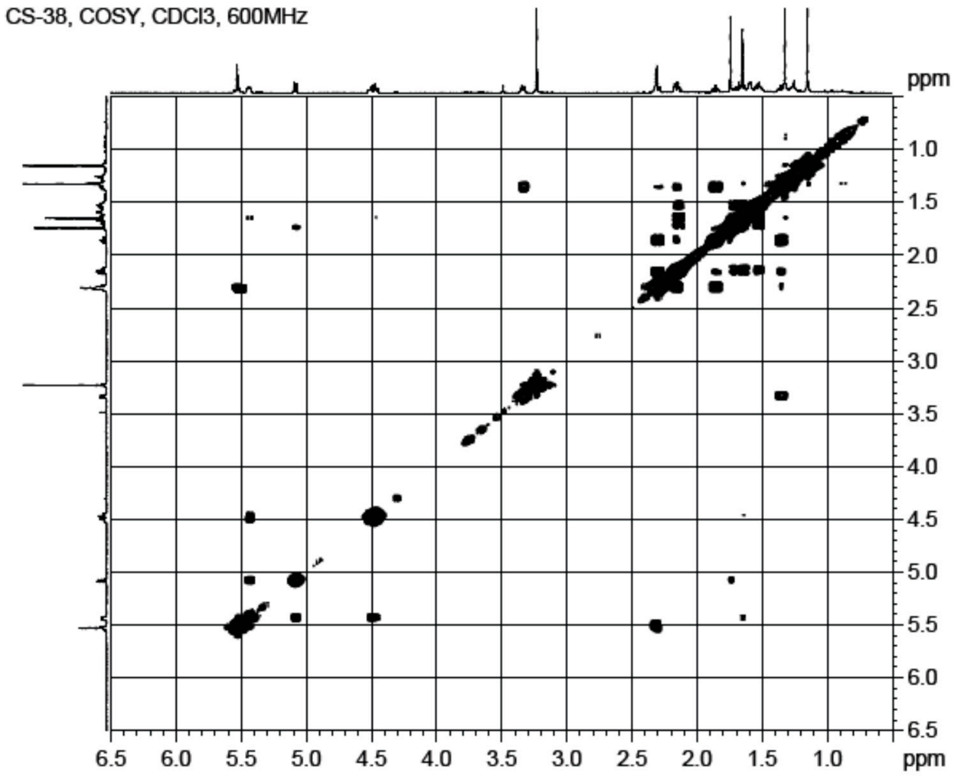

Figure S47. COSY spectrum of compound **7** in $CDCl_3$.

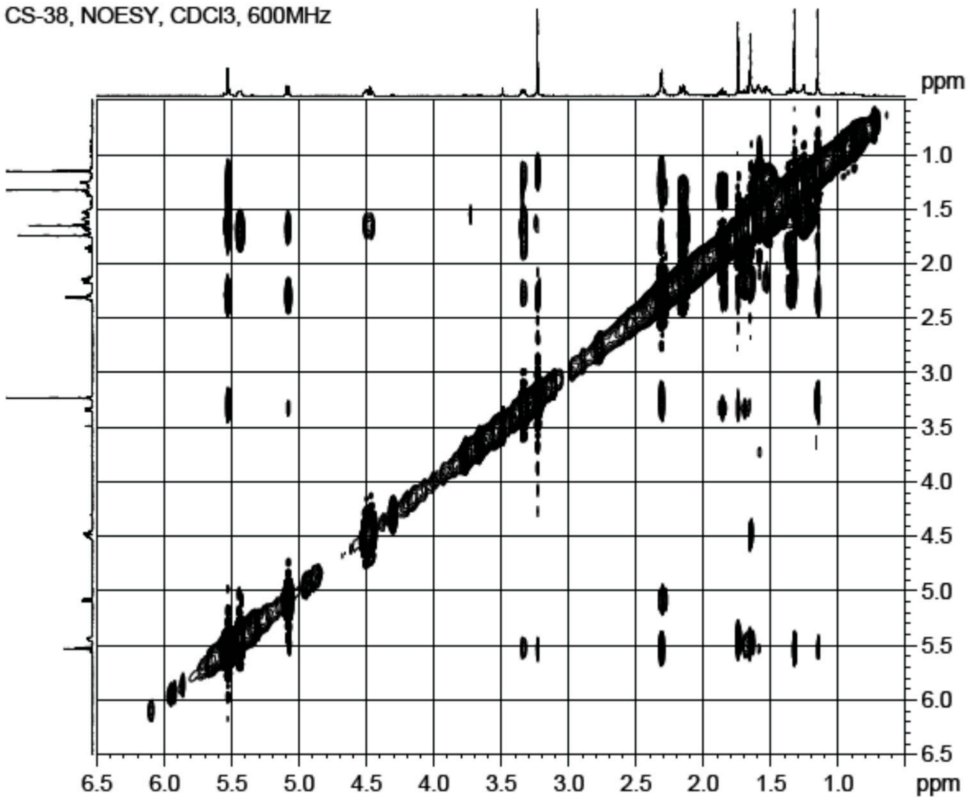

Figure S48. NOESY spectrum of compound **7** in $CDCl_3$.

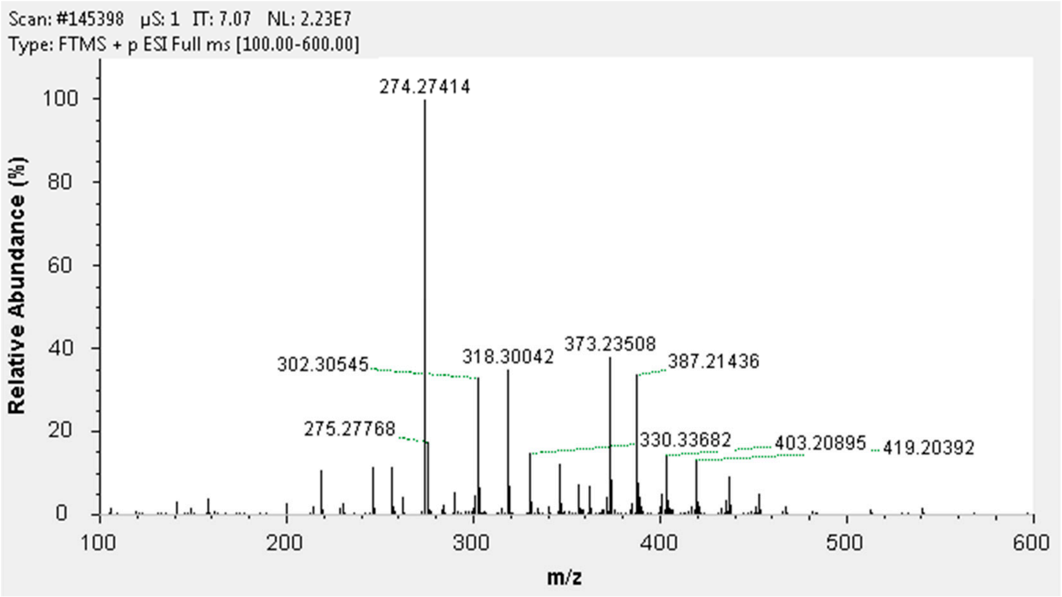

Figure S49. HRESIMS spectrum of compound **7**.

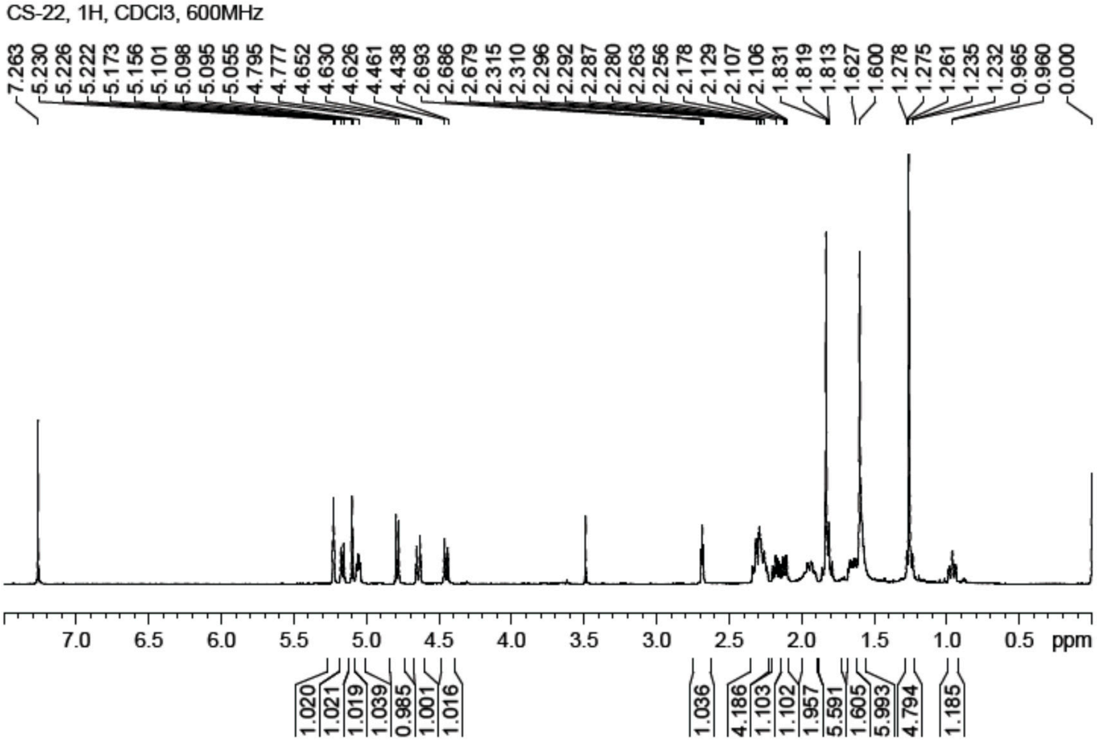

Figure S50. ^{1}H NMR spectrum (600 MHz) of compound **8** in $CDCl_3$.

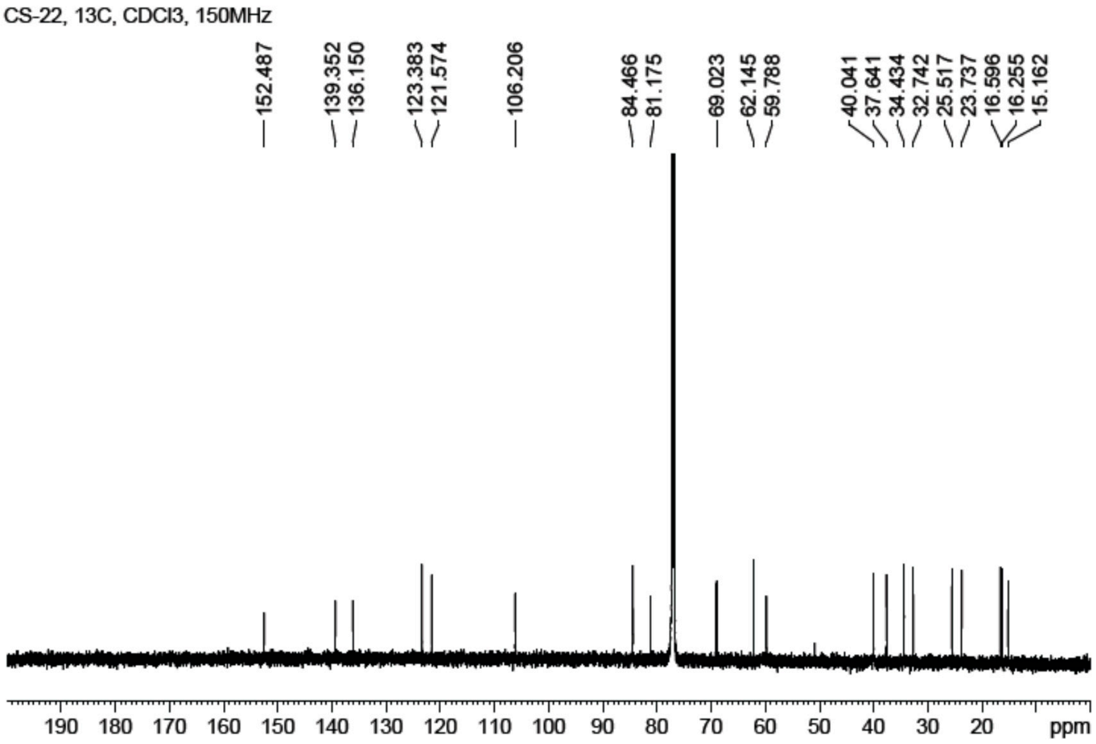

Figure S51. ^{13}C NMR spectrum (150 MHz) of compound **8** in $CDCl_3$.

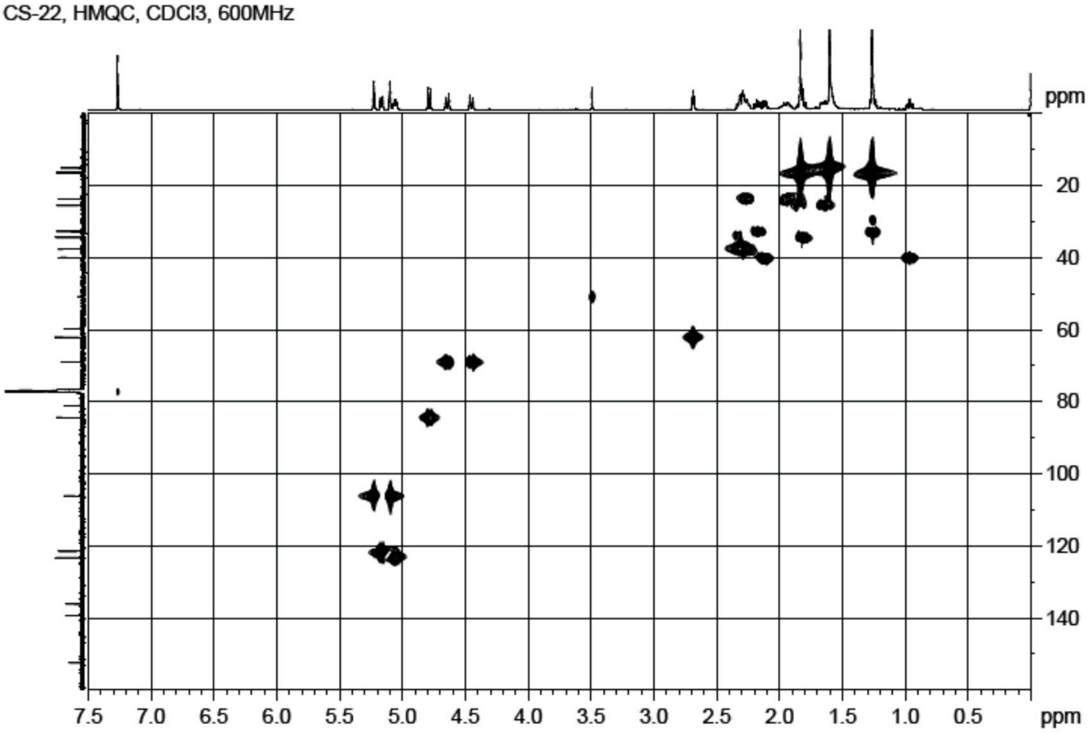

Figure S52. HMQC spectrum of compound **8** in $CDCl_3$.

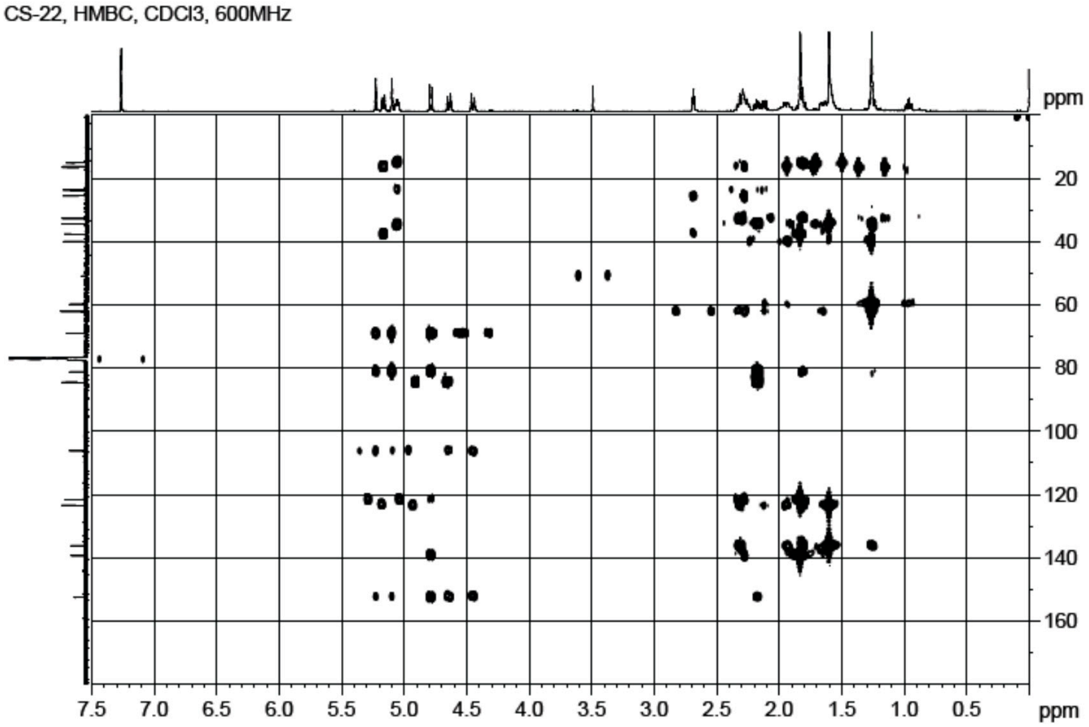

Figure S53. HMBC spectrum of compound **8** in $CDCl_3$.

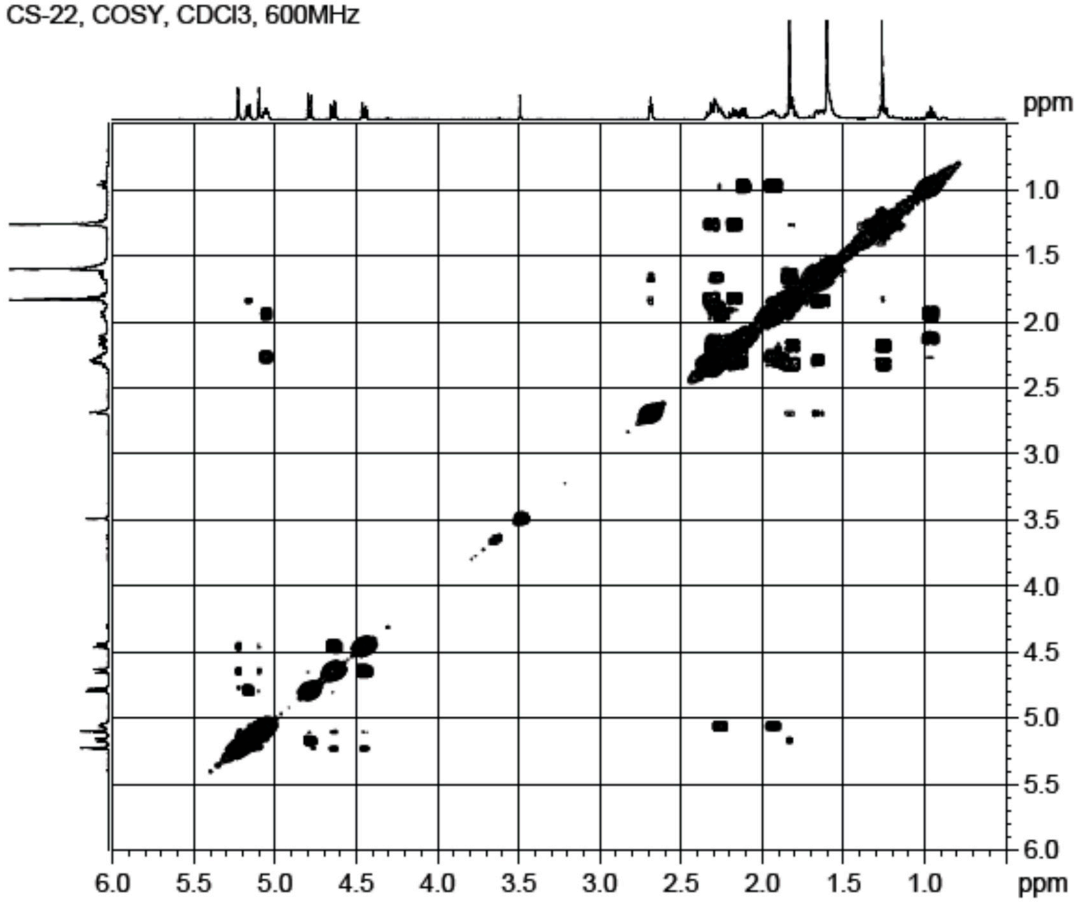

Figure S54. COSY spectrum of compound **8** in $CDCl_3$.

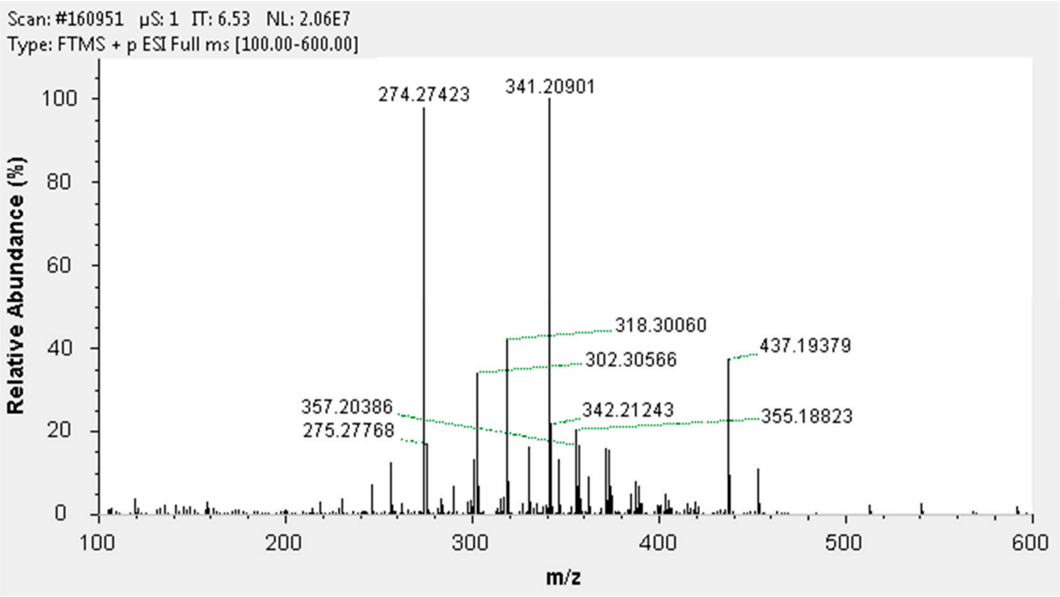

Figure S55. HRESIMS spectrum of compound **8**.

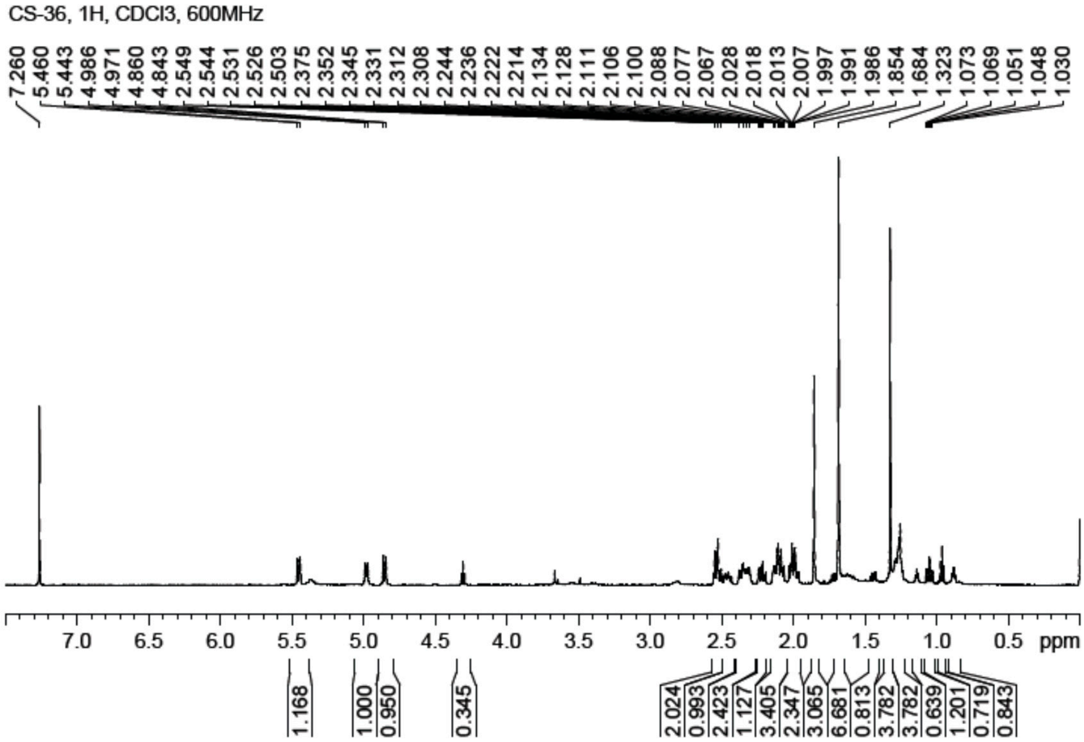

Figure S56. ^{1}H NMR spectrum (600 MHz) of compound **9** in $CDCl_3$.

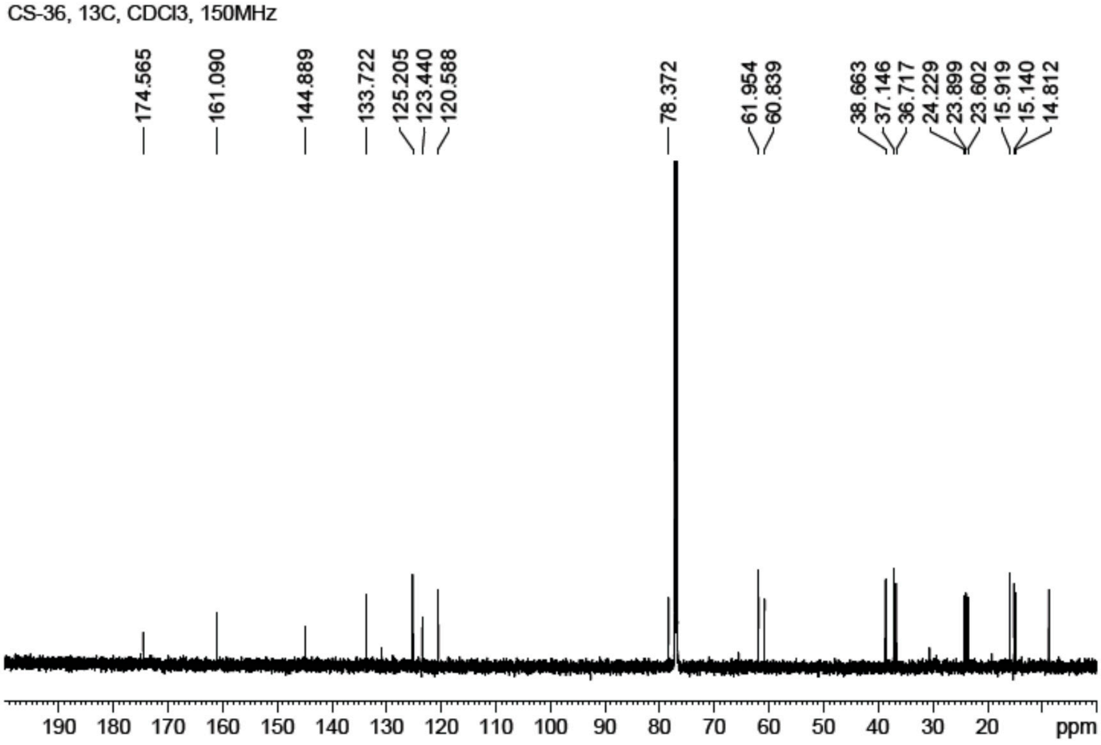

Figure S57. ^{13}C NMR spectrum (150 MHz) of compound **9** in $CDCl_3$.

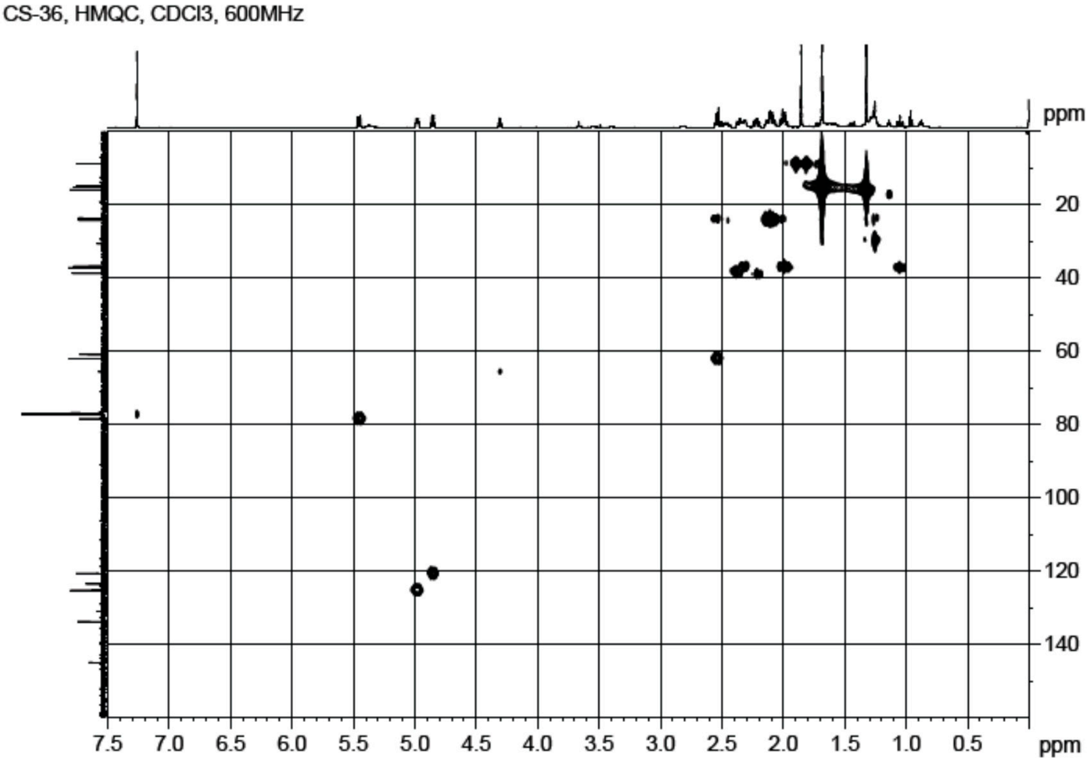

Figure S58. HMQC spectrum of compound **9** in $CDCl_3$.

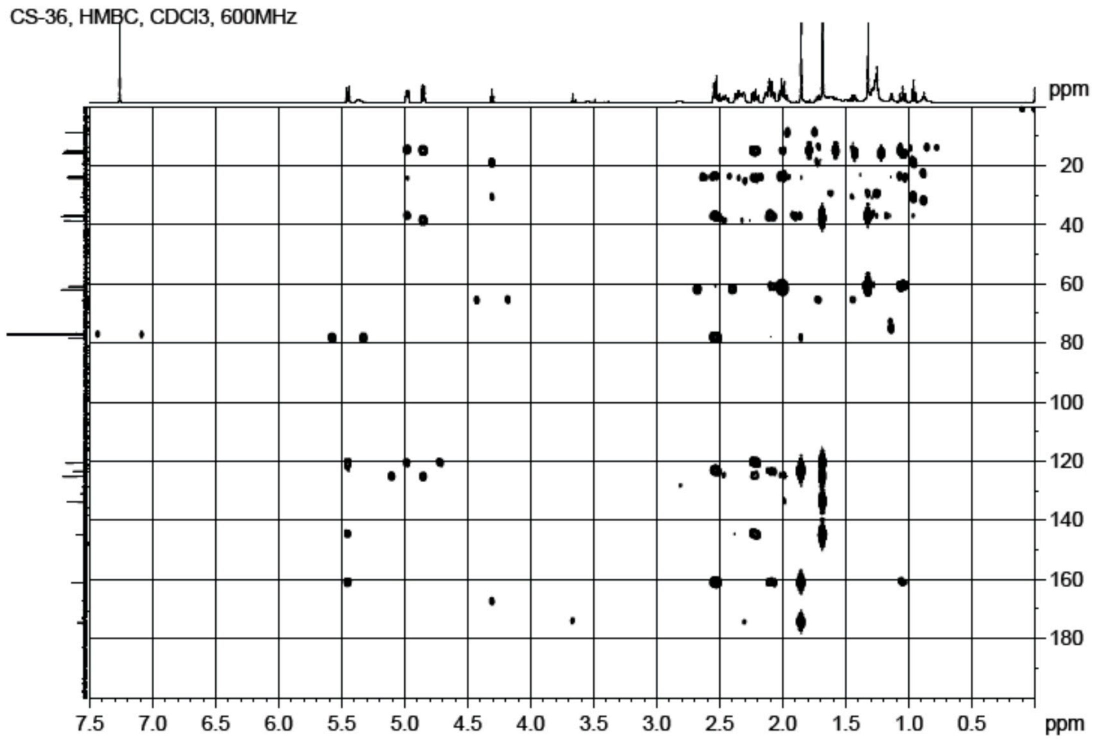

Figure S59. HMBC spectrum of compound **9** in $CDCl_3$.

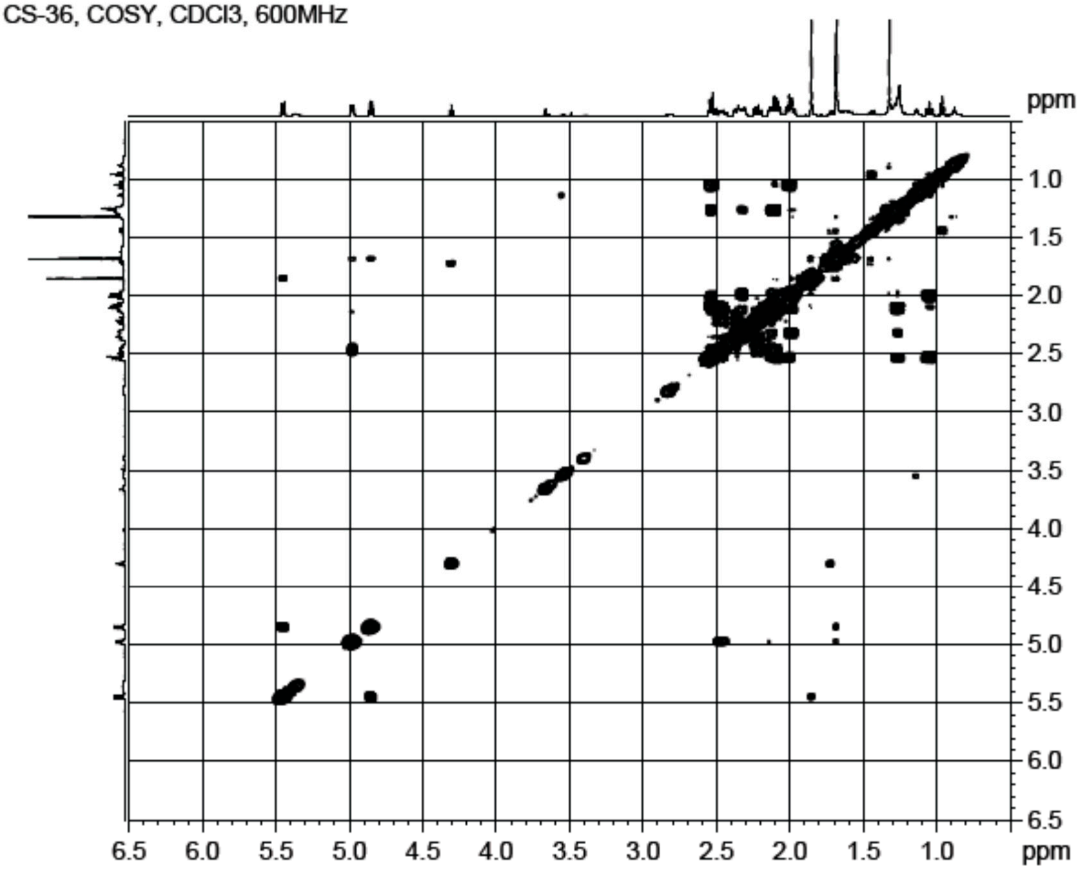

Figure S60. COSY spectrum of compound **9** in $CDCl_3$.

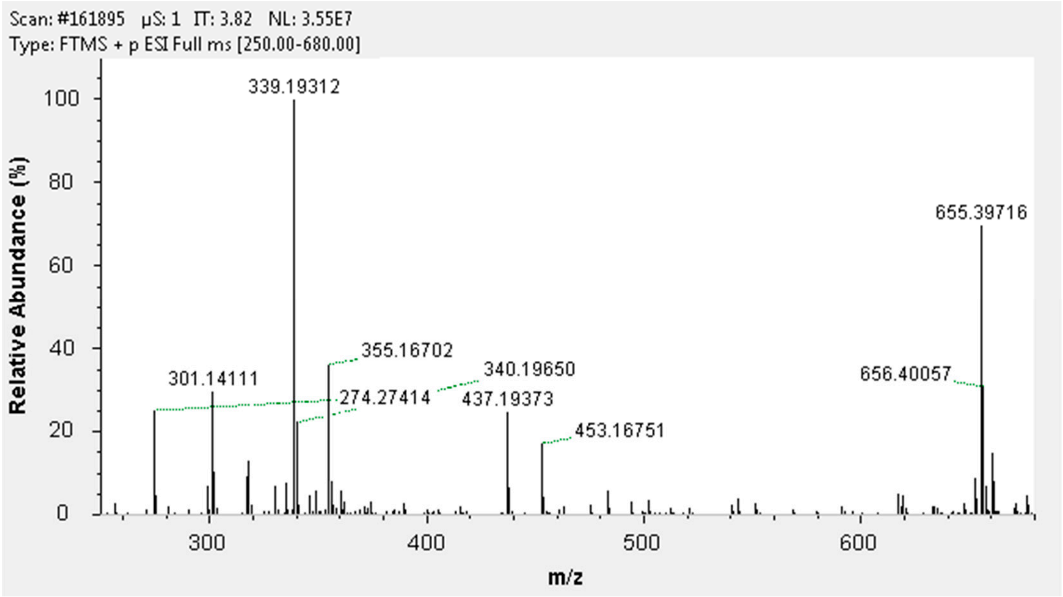

Figure S61. HRESIMS spectrum of compound **9**.

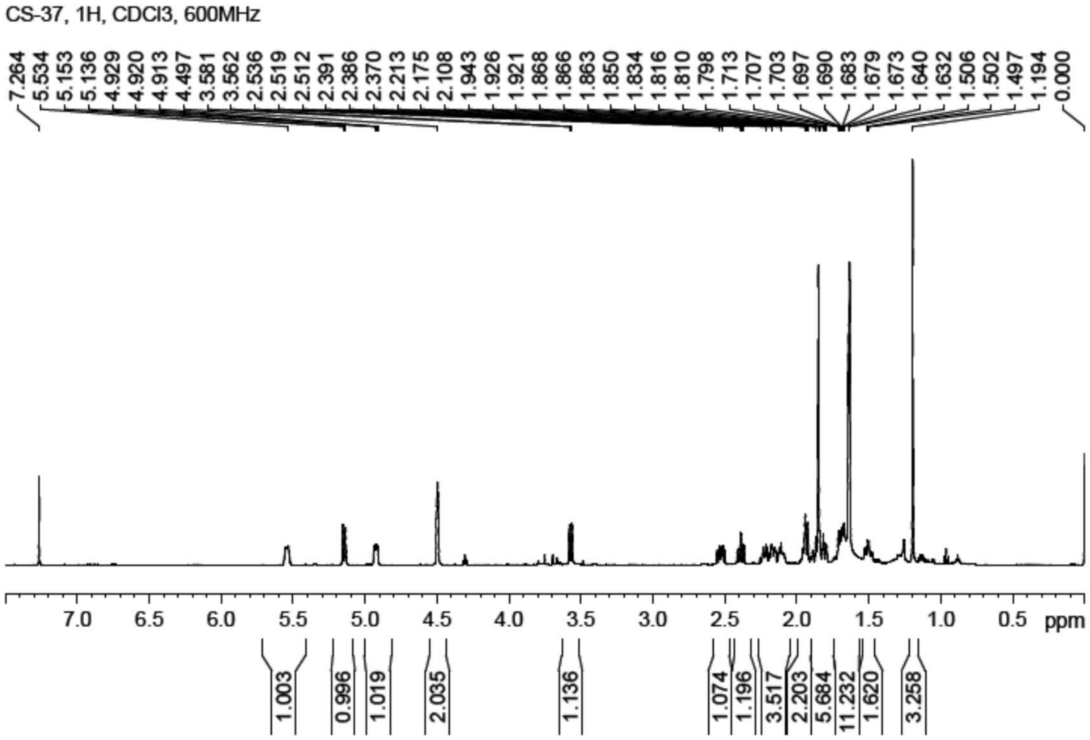

Figure S62. ^{1}H NMR spectrum (600 MHz) of compound **10** in $CDCl_3$.

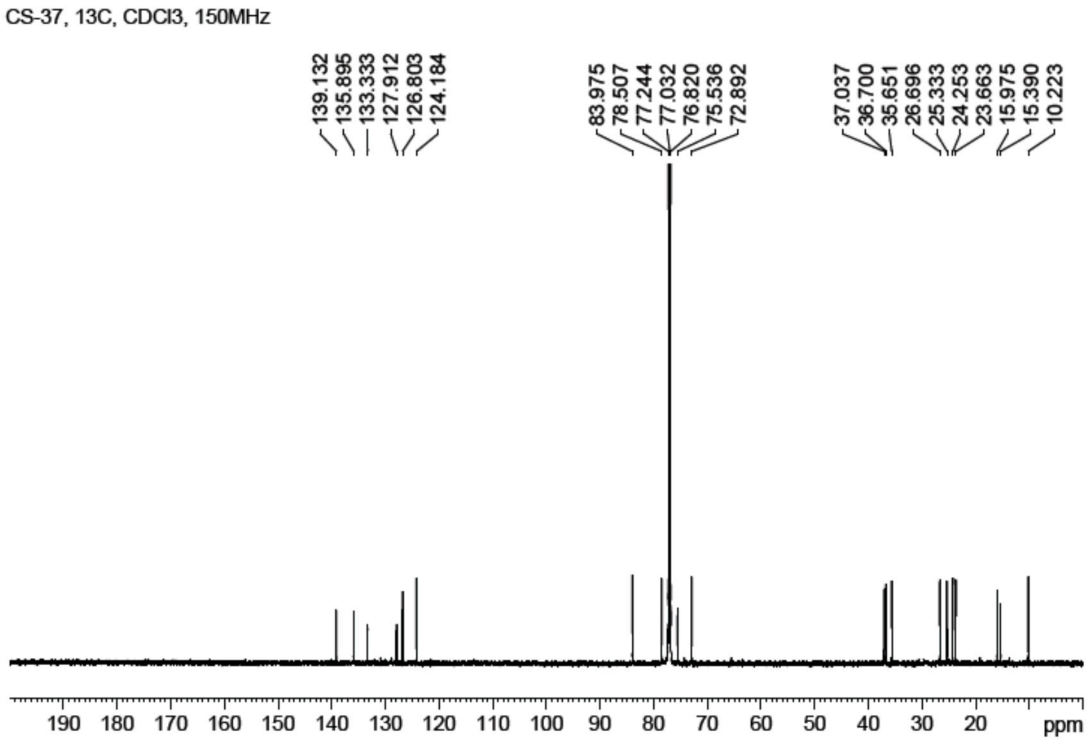

Figure S63. ^{13}C NMR spectrum (150 MHz) of compound **10** in $CDCl_3$.

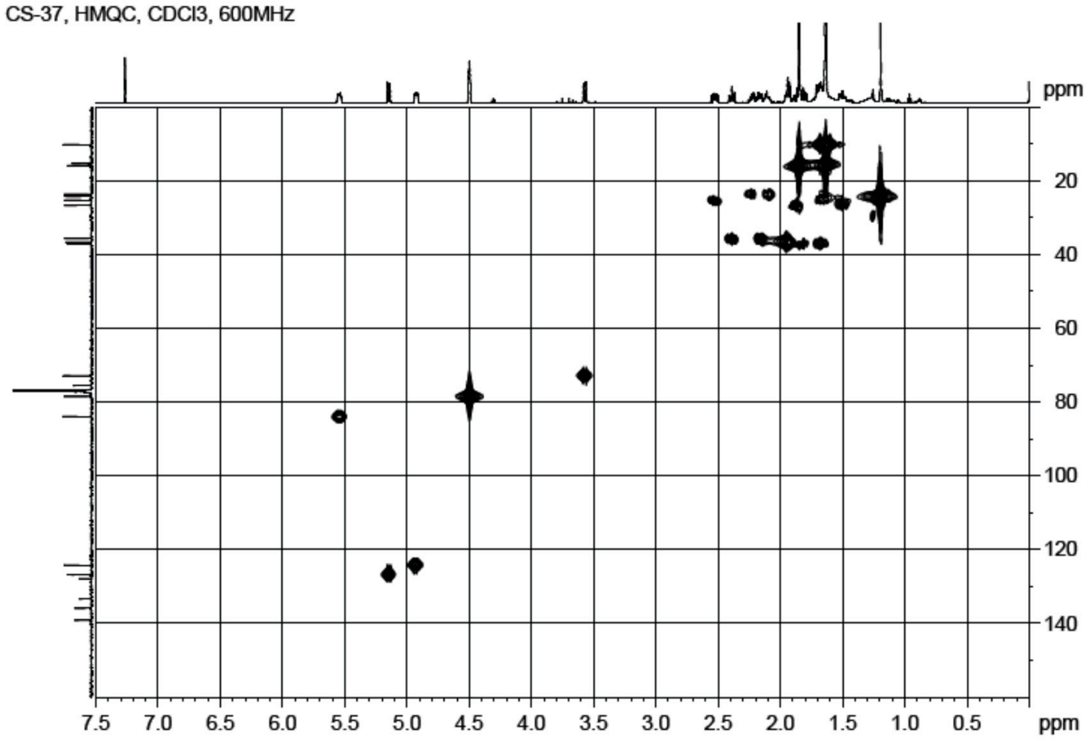

Figure S64. HMQC spectrum of compound **10** in $CDCl_3$.

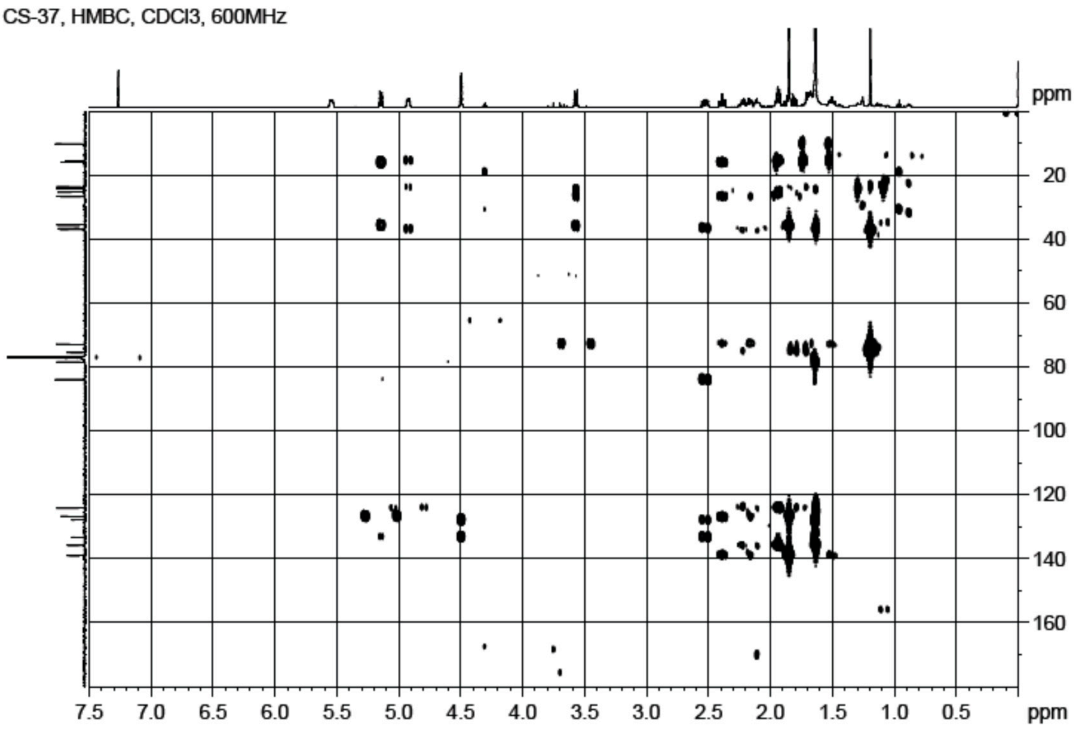

Figure S65. HMBC spectrum of compound **10** in $CDCl_3$.

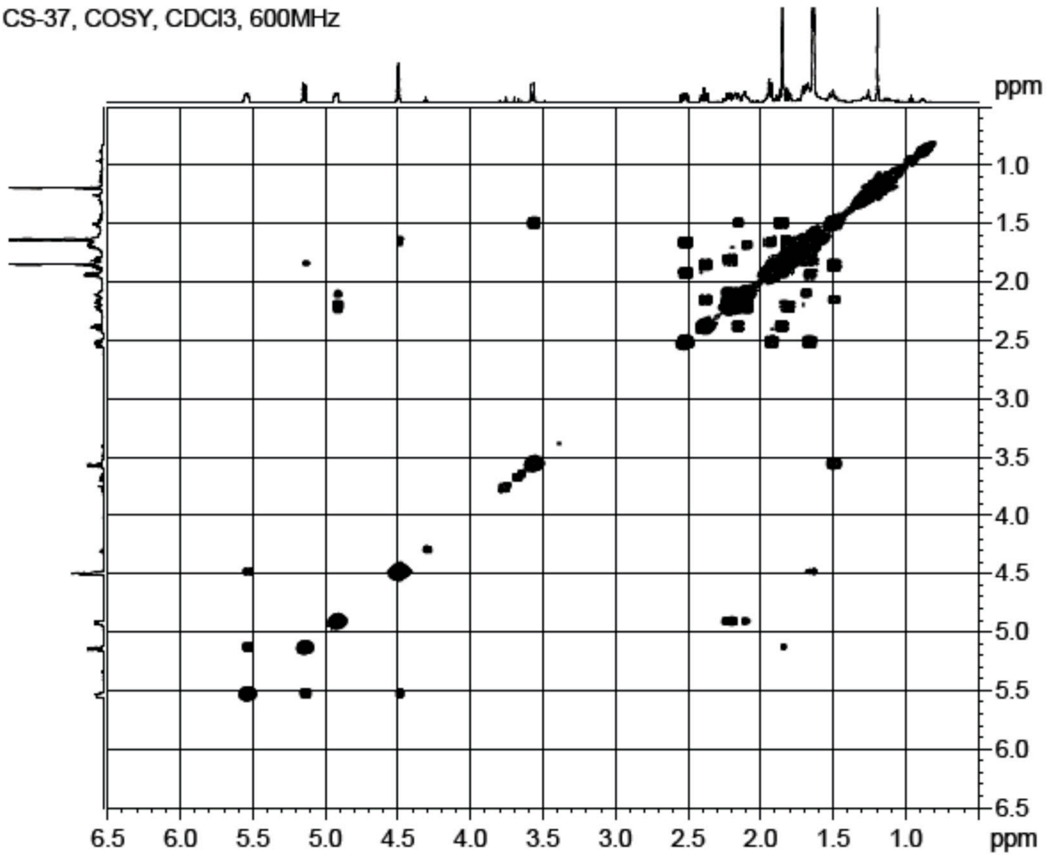

Figure S66. COSY spectrum of compound **10** in $CDCl_3$.

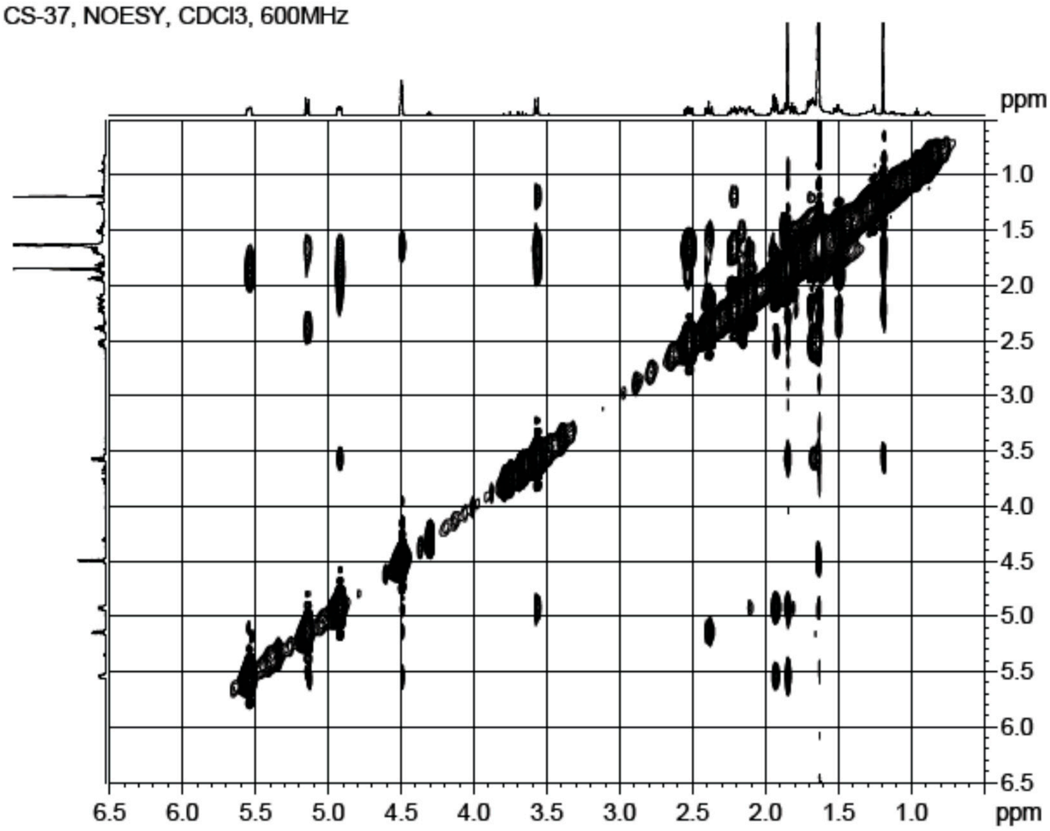

Figure S67. NOESY spectrum of compound **10** in $CDCl_3$.

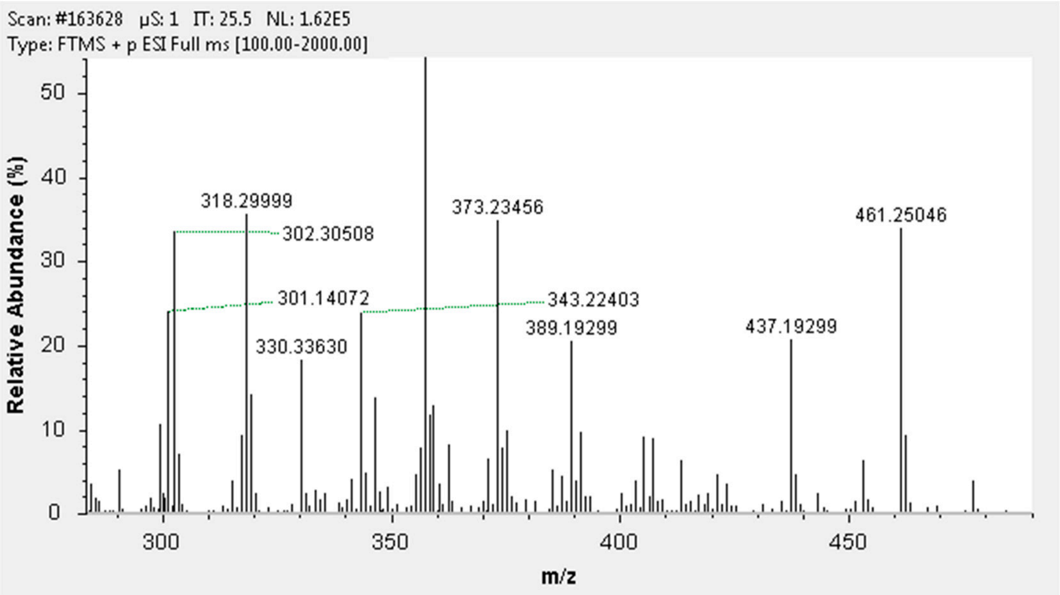

Figure S68. HRESIMS spectrum of compound **10**.
